# Origin of the structure-directing effect resulting in identical topological open-framework materials

**DOI:** 10.1038/srep14940

**Published:** 2015-10-08

**Authors:** Liang Xin, Huai Sun, Ruren Xu, Wenfu Yan

**Affiliations:** 1 State Key Laboratory of Inorganic Synthesis and Preparative Chemistry, College of Chemistry, Jilin University, 2699 Qianjin Street, Changchun 130012, PR China; 2 College of Chemistry and Chemical Engineering, Shanghai Jiaotong University, Shanghai 200240, PR China

## Abstract

In the synthesis of zeolites and related crystalline materials with open-frameworks, a single structure is obtained in the presence of many different templates, known as the “one-structure/multiple-templates” phenomenon. However, the reasons behind this phenomenon have yet to be elucidated. By analyzing the possible starting point of crystallization in several “one-structure/multiple-templates” systems and applying the molecular dynamics simulation to such systems, we found that the template-framework binding free energy level or charge transfer (exchange) degree was the key to the structure-directing effect of a template. This discovery explains why the structure-directing effect of a template can be affected by many variables, such as the nature of the source materials, molar composition of the initial reaction mixture (recipe), mineralizers, type of solvent, and heating temperature. In the synthesis of zeolites and related crystalline materials with open-frameworks, the template or organic additive played a topological structure-directing role instead of a structure-directing role.

Zeolites and related crystalline materials with open-frameworks, which have periodic three-dimensional (3D) frameworks, well-defined pore structures, and accessible voids, have widespread applications in catalysis, ion exchange, separation, and adsorption^1^,^2^,^3^. In addition to mining, hydro/solvothermal synthesis is an alternative way to obtain such materials. The synthesis of such materials typically involves mixing inorganic ion sources to provide the atoms for the framework, a solvent, and a third component or additive, typically an organic species, in an appropriate molar ratio, and heating the resulting mixture in an autoclave at elevated temperatures for a time ranging from a few hours to weeks. The third component or additive is critical for obtaining a specific structure. Without this additive, the specific structure cannot be obtained, and it is denoted as the “structure-directing agent (SDA)” or “template”^4^,^5^. If a specific structure can be obtained in the presence of an additive, we say that this additive is a structure-directing agent or template for this specific structure, and this additive has a specific structure-directing ability for this structure. Because the structure-directing agent is critical for the formation of a specific structure, understanding the structure-directing effect is then important in the rational synthesis of crystalline materials with desired open-frameworks. Although the exact role of the structure-directing agents is still far from being properly understood, it is believed that the charge distribution and the size and geometric shape of a template are the causes for structure-directing^4^.

In the synthesis of zeolites and related crystalline materials with open-frameworks, unique “one-structure/multiple-templates” phenomena are often observed. For example, AlPO_4_-5, a well-known aluminophosphate molecular sieve with a one dimensional (1D) 7.3 × 7.3 Å channel, can form in the presence of any one of more than 85 organic species, the smallest being isopropylamine (3 carbons) and the largest, hexabutyl-l,6-hexanediammonium (30 carbons)^6^. These AlPO_4_-5 structures give almost identical XRD patterns. Although the exact role that these organic species play in the synthesis is not been well understood, it is known that they have the same structure-directing ability as AlPO_4_-5.

Similar to other crystalline materials, the formation process of zeolites and related crystalline materials with open-frameworks is composed of a nucleation stage and nuclei growth stage^5^. The nuclei contain the basic building units of the resulting open framework. In the nucleation stage, the structure-directing agent organizes oxide tetrahedra or small structural building units (fragments) into a particular geometric topology around itself and thus provides the initial starting points for a particular structure type. The type of structure ultimately formed is determined by this step, and the structure-directing effect is realized in this stage. However, the concept and structure of the nucleus has never been clearly defined or exactly determined, and the nuclei have never been separated during the synthesis of such materials.

Recently, we reviewed the crystallization processes for zeolites and related crystalline materials with open-frameworks and found out that the formation or crystallization of such materials from the isolated molecular precursor species with the aid of solvent molecules is a temporally evolving process. This fundamental property can be used to study the events in the early stage of the crystallization process^7^. A reversed temporal evolution can be applied to the structures to obtain structural information for the species formed in the very early stages of the crystallization process, which are ultimately included in the structure of the resulting open-framework. In the crystallization process of aluminophosphates with open-frameworks for example, the condensation reaction between the Al and P sources immediately starts to form small inorganic fragments once all of the necessary source materials are mixed, which are then further assembled around the structure-directing agent to form an inorganic-organic composite with a specific configuration (denoted as the starting point of crystallization or the core unit)^7^,^8^. The core unit will further capture the new structure-directing agents and other small inorganic fragments to complete the growth of the core unit towards the crystal. The crystallization process can be treated as a successive assembly process of the small inorganic fragments and the structure-directing agents around the core unit, which plays a similar role in the nuclei. Thus, the formation of core units occurring in the early stages of crystallization would be the most important events for the formation of a specific structure, which highlights the structure-directing effect of the additives. With the reversed temporal evolution process, a core unit for each structure can be obtained. Analyzing the core units will allow us to obtain new knowledge of the nature or origin of the structure-directing effect and the crystallization pathway of specific structures.

Here, we analyzed the core units and repeat units of several “one-structure/multiple-templates” systems, including aluminophosphate molecular sieves with a chabazite (CHA) topology (denoted as topology-I)^9^,^10^,^11^, layered aluminophosphates with a 4 × 6 × 8 network topology (denoted as topology-II)^12^,^13^,^14^,^15^,^16^, microporous gallophosphates with a ULM-3 topology (denoted as topology-III)^17^,^18^, and layered zinc phosphates (denoted as topology-IV)^19^,^20^. The repeat unit of each structure contains all of the structural information of the framework and the non-framework species. Structures directed by different organic additives but possessing the same topology were actually different from each other. In the crystallization of these compounds, the organic additives played a topological structure-directing role instead of a structure-directing role. Two structures can have both the same topology and significantly different crystallization pathways. This new discovery represents a step forward in understanding the roles that structure-directing agents play in the formation of crystalline materials with open-frameworks.

## Results and Discussion

### Core units and repeat units analyses

In this study, four typical “one-structure/multiple-templates” systems were selected: aluminophosphate molecular sieves with a CHA topology^9^,^10^,^11^, layered aluminophosphates with a 4 × 6 × 8 network topology^12^,^13^,^14^,^15^,^16^, microporous gallophosphates with a ULM-3 topology^17^,^18^, and layered zinc phosphates^19^,^20^. Aluminophosphate molecular sieves with a CHA topology were obtained in the presence of morpholine (denoted as AlPO_4_-CHA-morpholine)^9^, pyridine (denoted as AlPO_4_-CHA-pyridine)^10^, piperidine (denoted as AlPO_4_-CHA-piperidine)^11^, isopropylamine (denoted as AlPO_4_-CHA-isopropylamine)^11^, and diethylamine (denoted as AlPO_4_-CHA-diethylamine)^11^, and layered aluminophosphates with a 4 × 6 × 8 network topology were obtained under solvothermal conditions in the presence of tetramethylethylenediamine^12^, cyclobutylamine and piperidine^13^, ethylamine^14^, 1,5-diaminopentane^15^, and *n*-propylamine^16^. The organic amines that can direct the microporous gallophosphates with ULM-3 topology are 1,3-diaminopropane^17^, 1,4-diaminobutane^18^, and 1,5-diaminopentane^18^. For two-layered zinc phosphates with the same topology, the organic additives were 3-methylaminopropylamine^19^ and N,N’-dimethylethylenediamine^20^, respectively. Except for AlPO_4_-CHA-morpholine, the structures of all other CHA-type compounds were clearly determined through single-crystal X-ray diffraction structural analyses. The crystallographic data for these four typical “one-structure/multiple-templates” systems are listed in Table 1, and the corresponding synthesis conditions are listed in Supplementary Table S1. The crystallographic and structural refinement data, atomic coordinates, and bond lengths and angles for the unpublished AlPO_4_-CHA-piperidine, AlPO_4_-CHA-iso-propylamine, and AlPO_4_-CHA-diethylamine are listed in Supplementary Tables S2-S10.

Molecular sieve chabazite is a natural zeolite occurring in basaltic rocks and crystallizes in an R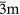 space group^21^,^22^. The structure of chabazite consists of double six-rings forming a ***cha*** cage^23^. Its silicoaluminophosphate analogue (denoted as SAPO-34) is obtained under hydrothermal conditions in the presence of tetraethylammonium ion^24^, which is an important catalyst for the methanol-to-olefins (MTO) process. The aluminophosphate analogue of chabazite (denoted as AlPO_4_-CHA) is first hydrothermally synthesized in the presence of morpholine and crystallizes in the 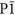 space group^9^. Pyridine, piperidine, isopropylamine, and diethylamine can also direct the crystallization of AlPO_4_-CHA under hydro/solvothermal conditions. All AlPO_4_-CHAs crystallize in the same 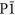 space group with similar cell parameters. Each ***cha*** cage contains two protonated amine molecules. According to the present understanding of the structure-directing effect, these organic species have the same structure-directing ability for AlPO_4_-CHA. The very early stages of crystallization should be the same or very similar. To investigate this, we analyzed the core units of these five AlPO_4_-CHAs.

**Figure 1:**
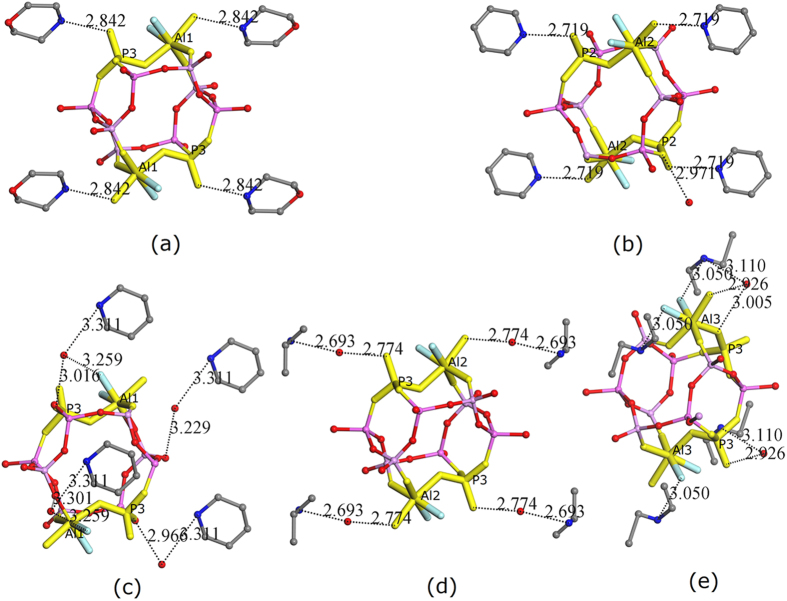
The repeat units of double six-ring and near non-framework species, as well as the highlighted core units with a close contact of 3.0 Å for AlPO_4_-CHAs. The double six-rings were placed with very similar orientations. The structure-directing agents were (**a**) morpholine, (**b**) pyridine, (**c**) piperidine, (**d**) isopropylamine, and (**e**) diethylamine. Key Al and P atoms are labeled with their names. Oxygen, nitrogen, fluorine, and carbon atoms are labeled with red, blue, cyan, and grey colors, respectively.

Figure 1 shows the repeat unit of the double six-ring and the near non-framework species, as well as the highlighted core unit of AlPO_4_-CHAs. The double six-rings are placed in very similar orientations. In the framework of AlPO_4_-CHA-morpholine (Fig. 1(a)), each double six-ring is surrounded by four near protonated morpholine molecules. Each morpholine has one strong hydrogen-bond with the oxygen atom of either a P- or Al-centered tetrahedron. The P- and Al-centered tetrahedra form a dimer, suggesting that this dimer might be pre-formed and subsequently meets the morpholine molecules. The protonated morpholine and the dimer form the core unit of AlPO_4_-CHA-morpholine, as highlighted in Fig. 1(a). Although there are two such dimers in the double six-ring of AlPO_4_-CHA-morpholine, it is unlikely that they are formed simultaneously and then react with other parts of the double six-ring to complete it. More likely, the core unit containing the dimer and two protonated morpholine molecules is first formed and then reacts with other parts of the double six-ring to form an incomplete double six-ring, which will be completed upon capture of the other dimer. Once the double six-ring is completed, the latter joined dimer can capture two protonated morpholine molecules. However, it is also possible that the double six-ring is completed by capturing a pre-formed core unit (i.e. a dimer and two protonated morpholine). In AlPO_4_-CHA-pyridine, the core unit is similar to that of AlPO_4_-CHA-morpholine, except for a stronger H-bonding interaction and an extra water molecule (Fig. 1(b)). Although the shape and size of piperidine is very similar to that of morpholine and pyridine, the core unit of AlPO_4_-CHA-piperidine (Fig. 1(c)) is significantly different than those of AlPO_4_-CHA-morpholine and AlPO_4_-CHA-pyridine. The positions of the piperidine around the double six-ring are significantly different than those of morpholine and pyridine. No H-bonding between the protonated piperidine and the inorganic part was observed. Water molecules acted as bridges for piperidine and the inorganic part. Therefore, the way that piperidine directs the framework of AlPO_4_-CHA should be different from that of morpholine and pyridine, as well as their crystallization pathways. A new scheme was observed when isopropylamine was used as the organic additive (Fig. 1(d)). Water molecules were located at the middle position of the protonated isopropylamine and the inorganic part and had strong H-bonding interactions with both of them. The topological position of the protonated isopropylamine and neutral water molecule is similar to that of morpholine and pyridine in the corresponding structures. Thus, the framework of AlPO_4_-CHA-isopropylamine is perhaps directed by the composite of the protonated isopropylamine and the neutral water molecule. The way in which isopropylamine directs AlPO_4_-CHA should be different from that of the above three amines. In addition to the above four amines, diethylamine can also direct the framework of AlPO_4_-CHA under solvothermal conditions (Fig. 1(e)). Surprisingly, diethylamine has strong H-bonding interactions with fluorine instead of the oxygen atom of the inorganic part, indicating a completely different structure-directing mechanism. The detailed structures of the AlPO_4_-CHAs are different even though they have the same framework-topology, and the way amines direct the topology and crystallization pathways of the AlPO_4_-CHAs are also different. These amines have the same topological structure-directing ability (directing the same topological structure), rather than the same structure-directing ability (directing the same structure).

Layered aluminophosphates with 4 × 6 × 8 network topology can also be solvothermally synthesized in the presence of tetramethylethylenediamine, cyclobutylamine and piperidine, ethylamine, 1,5-diaminopentane, and *n*-propylamine (Table 1 and Supplementary Table S1). Their repeating capped six-ring units and near non-framework species, as well as the highlighted core unit are shown in Supplementary Fig. S1. In these layered aluminophosphates, the protonated amines reside in the interlayer region and interact with the layers above and below through H-bonding and other non-bonding interactions such as Coulomb and van der Waals (VDW) interactions. For clarity, the core units of the layers above and below are shown separately. Unlike AlPO_4_-CHAs, these layered aluminophosphates crystallize in different space groups. In the core units of these layered aluminophosphates, the number of H-bonds between the amine and the inorganic part, the distance between the nitrogen of the protonated amine and the oxygen of the inorganic part, and the topological position of the protonated amines around the caped-six-rings are different from each other. However, these layered aluminophosphates ultimately possess the same topology, indicating that the way the amines direct these layered aluminophosphates and the crystallization pathways of these layered aluminophosphates are different. These amines should also have the same topological structure-directing ability, rather than the same structure-directing ability.

Gallophosphate and zinc phosphate are another two large families of crystalline materials with open-frameworks^25^. In the gallophosphate family, the topology of microporous ULM-3 can be obtained under hydrothermal conditions in the presence of 1,3-diaminopropane, 1,4-diaminobutane, and 1,5-diaminopentane (Table 1 and Supplementary Table S1). Their repeat units, near non-framework species, and the highlighted core unit are shown in Supplementary Fig. S2. They crystallize in the same space group as Pbca with very similar unit cell parameters. However, their core units and the topological position of the diamines are completely different, suggesting that these diamines have the same topological structure-directing ability, rather than the same structure-directing ability.

**Figure 2:**
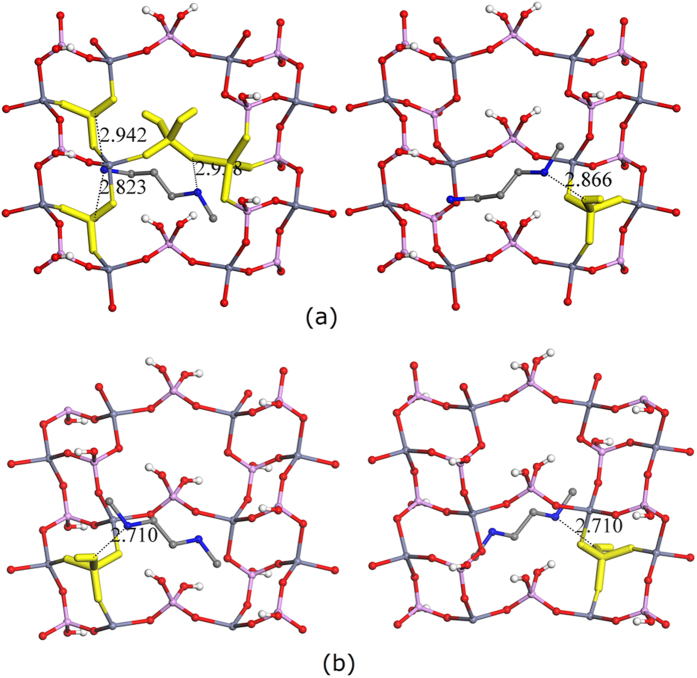
The repeat units and near non-framework species, as well as the highlighted core units of layered zinc phosphates with a close contact of 3.0 Å. The repeat units were placed in very similar orientations. The structure-directing agents were (**a**) 3-methylaminopropylamine (Left: above layer; Right: below layer) and (**b**) N,N’-dimethylethylenediamine (Left: above layer; Right: below layer). Phosphorus, zinc, oxygen, nitrogen, hydrogen, and carbon atoms are labeled with pink, dark cyan, red, blue, white, and grey colors, respectively.

Similar to other metal phosphates with open-frameworks, the synthesis of crystalline zinc phosphates with open-frameworks is typically performed in the presence of organic amines. In the presence of 3-methylaminopropylamine and N,N’-dimethylethylenediamine, two zinc phosphates with the same topology were obtained under hydrothermal conditions (Table 1 and Supplementary Table S1). Their repeat units, near non-framework species, and the highlighted core units are shown in Fig. 2. In these layered zinc phosphates, the protonated amines reside in the interlayer region and interact with the layers above and below through H-bonding and other non-bonding interactions. For clarity, the core units of the layers above and below are shown separately. Unlike AlPO_4_-CHAs and microporous gallophosphates with ULM-3 topology, these layered zinc phosphates crystallize in different space groups, but with similar unit cell parameters. In the core unit of the zinc phosphate directed by 3-methylaminopropylamine, two N atoms have strong H-bonding interaction with the P-centered tetrahedra located in the above layer (Fig. 2(a) left), while only one N atom has strong H-bonding to the P-centered tetrahedron located in the layer below (Fig. 2(a) right). However, in the core unit of another layered zinc phosphate directed by N,N’-dimethylethylenediamine, one N atom has strong H-bonding interaction with the P-centered tetrahedron located in the above layer (Fig. 2(b) left), while another N atom of this amine has the same strong H-bonding interaction with the P-centered tetrahedron located in the layer below (Fig. 2(b) right). Therefore, the core units of these two layered zinc phosphates are different even though they have the same topology (repeat unit). Again, these data suggest that the way amines direct these two zinc phosphates with same topology is distinct, and these amines have the same topological structure-directing ability instead of the same structure-directing ability.

After analyzing the core units of the above four typical “one-structure/multiple-templates” systems, we found out that the organic additives actually played a topological structure-directing role instead of a structure-directing role. Although two structures may have the same topology, their crystallization pathways may be significantly different from each other. This new discovery may help to understand the real roles of structure-directing agents in the formation of crystalline materials with open-frameworks.

### Molecular dynamics simulation

To reveal the origin of the structure-directing effect resulting in the crystalline open-framework materials with the same compositions and topologies, the early stages of the crystallization process need to be observed in real time both experimentally and theoretically. However, this is not currently possible given the present characterization and simulation techniques. In fact, the stability of the core unit or the onset of the crystallization (nucleus) determines the type of structure formed. Thus far, the estimation of the stability of the core unit or the onset of crystallization cannot be achieved by the present simulation techniques. Therefore, we applied molecular dynamics simulations to the above four “one-structure/multiple-templates” systems.

Based on the calculations, the same topology exhibits similar volumetric and energetic features. Table 2 lists the framework cell volumes (*V*_FM_), framework energies (*E*_FM_), Helmholtz binding free energies (*F*_B_), total charges of the SDA molecules (*Q*_SDA_), and formal charges on the SDA molecules (*FC*_SDA_), grouped by the topology type defined in Table 1. To compare different structures, the framework cell volume and framework energy were normalized by the number of tetrahedral centers, and the formal charge and total atomic charge of SDA were normalized by the number of SDAs. The Helmholtz binding free energy (*F*_B_) is defined as the free energy change of bringing the SDA and the framework together from infinite separation, a negative value indicates that the complex is more stable than the separated state. The binding free energy contains the energetic contribution from the non-bonding (electrostatic and VDW) interactions between the framework and SDA molecules, and the entropic contribution from the conformational flexibility of the framework and SDA molecules, which carries more information in comparison with previous calculations that only considered the energetic contributions^26^,^27^,^28^,^29^. For each topology, the average value and standard deviation (STD) of the average value are given. A common feature of the data in Table 2 is that the compounds with the same topology but different SDAs had similar energetic and electrostatic data. The frameworks are flexible to accommodate different SDAs, which is represented by the STD in volume and energies. The two-dimensional (2-D) layered structures were much more flexible than the three-dimensional (3-D) structures. The flexibility of frameworks originates from the wide (120°–170°) T-O-T angles and the low energy cost of bending the T-O-T angle.

The electrostatic energies were ignored in previous calculations^26^,^27^,^28^,^29^ because otherwise unrealistically large electrostatic energies would be obtained. This is presumably due to the inconsistency between the charge parameters and the VDW parameters in the underlying force fields. Using the QEq method, the atomic charges were not fixed by the force field charge parameters, but dynamically adjusted in response to the atom-atom distances, which were influenced by the VDW parameters. Therefore, despite the QEq method may not be accurate, there is an intrinsic consistency between the electrostatic and VDW interactions. The data in Table 2 shows that the Helmholtz binding free energy is roughly correlated to the charge transfer between SDA and zeolite framework, defined as the ratio of the calculated total charge (*Q*_SDA_) and formal charge (*FC*_SDA_) of SDA. When two oppositely charged species are close enough (e.g., less 5.0 Å) to each other, the charge transfer (or exchange) due to the electron cloud overlap will occur, which leads to the deviation of the total charge on the SDA from its formal charge. The data in Table 2 can be used to calculate the average ratios of *Q*_SDA_/*FC*_SDA_, which are 0.24, 0.44, 0.16, and 0.49 from topology I through IV. The order was roughly correlated with the average Helmholtz binding free energies (*F*_B_): −4.8, −7.0, −3.4, and −12.9 kcal/mol in the corresponding topology. Therefore, the charge transfer or exchange from the inorganic framework to the SDAs or *vice versa* plays an important role in stabilizing such composites by reaching an appropriate interaction strength, which is sensitive to the structures of the inorganic and the SDA and the distance between them.

**Figure 3:**
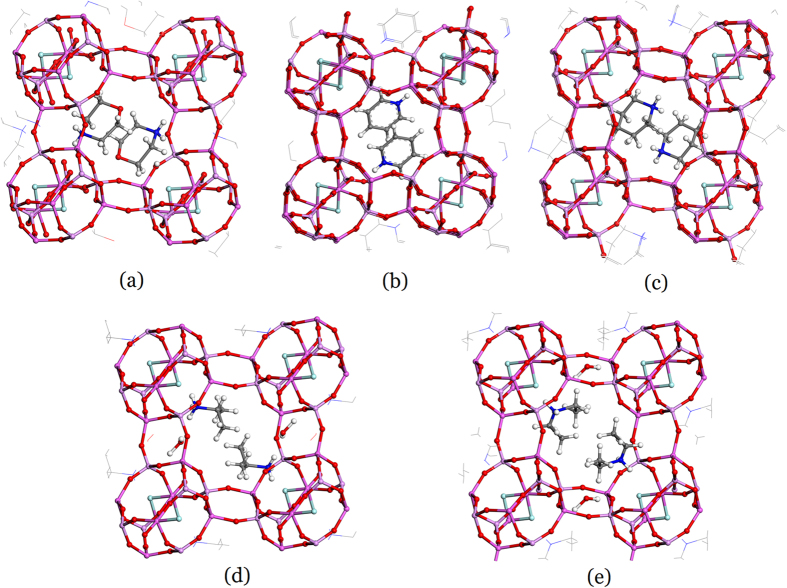
Similarity of aluminophosphate CHA topologies. The structure-directing agents were (**a**) morpholine, (**b**) pyridine, (**c**) piperidine, (**d**) isopropylamine, and (**e**) diethylamine. Oxygen, nitrogen, fluorine, and carbon atoms are labeled with red, blue, cyan, and grey colors, respectively.

Given the framework flexibility, the SDAs were similar in function with the same topology. Figure 3 shows the symmetrically independent SDAs in the frameworks of CHA, which uniquely represents the interaction pattern between the SDAs and the framework. The structures were taken from the experimental data. To compare differences in the structures, the figures were prepared with a fixed framework orientation. For the same topology, different SDAs are oriented differently because the negatively charged oxygen sites are more than the positive charged sites on the SDAs. However, the positively and negatively charged sites were matched similarly to lower the Helmholtz binding free energies. The SDA profiles of the 2-D structures are less clear than that of the 3-D structures, which is consistent with the large fluctuations in energies and charges in the 2-D structures. However, even with different SDAs, the combined SDAs had the same pattern. Overall, the profiles were not identical, and differences result from the flexible framework, producing different structures with the same topology.

The above discussion suggests that the formation of a stable core unit requires the appropriate inorganic fragments, SDA with appropriate topological position, and other factors, such as the ratio of the source species (i.e., recipe), solvent molecules (solvent type), extra additives (i.e., mineralizers), and heating temperature. Changing the topological position of the SDAs around the inorganic part (making a specific configuration) may result in core units with different binding free energy levels, which leads to the unique “one template/multiple structures” phenomenon in the synthesis of microporous crystals. However, combination of the appropriate inorganic part with different SDAs can also result in core units with the same Helmholtz binding free energy or interaction strength, which results in another unique “one structure/multiple templates” phenomenon in the synthesis of microporous crystals, as discussed in this work.

In conclusion, the crystalline compounds with the same composition and topological open-framework might be crystallized from different core units. These core units have a similar SDA-framework Helmholtz binding free energy level (i.e., interaction strength) and charge transfer or exchange degree. The structure-directing effect of a template can only be realized when a core unit with an appropriate Helmholtz binding free energy level is formed. The variables for the formation of a specific core unit include the inorganic portion (fragment), organic portion (type and number of the SDA), their topological position (distance and configuration), and other factors. Thus, the structure-directing effect of a template can be affected by many factors, such as the nature of the source materials, molar composition of the initial reaction mixture (recipe), mineralizers, type of solvent, and heating temperature. However, the key to the structure-directing effect of a template is the formation of a stable core unit with a specific Helmholtz binding free energy level and charge transfer or exchange degree.

## Methods

### Core unit

For a given crystalline structure with an open-framework, the unit cell was first multiplied by three or more times along the *x*, *y*, and *z* axes, forming a super cell. The repeat unit of the framework and the nearby framework species were reserved, and all other atoms were deleted to form a composite. The close contact of less than 3.0 Å between the oxygen atoms in water or the nitrogen and the oxygen atoms of the repeat unit were searched, which can create strong H-bonding interactions within the composite. The core unit, highlighted in the composite, was composed of the non-framework species and the strongly H-bonded inorganic species.

### Simulation

Calculations were carried out using the Forcite module in Materials Studio 7.0. For each structure, a simulation box was constructed from a unit cell with an initial configuration taken from the experimental CIF data. The periodic boundary condition was applied in the calculations. The UFF force field^30^ was applied to describe the bonding and non-bonding interactions, including long-range electrostatic and van der Waals (VDW) forces. The atomic charges were calculated using the charge equilibrium (QEq) method^31^, which adjusts atomic charges dynamically to represent the polarization effects. The combination of UFF and QEq has been used successfully to describe many different inorganic materials^32^. The long-range electrostatic energy was calculated using the Ewald summation, and the VDW interaction was calculated using a 12.5 Å cutoff with a tail correction. The time step of the simulations was 1.0 fs. For each simulation box, a 50 ps NPT simulation was carried out for equilibrium, and a 50 ps NVT simulation was used for data collection. The Nose-Hoover-Langevin thermostat^33^,^34^ and Parrinello barostat^35^ were used to control temperature (298 K) and pressure (1 atm), respectively. The Helmholtz binding free energy was calculated using the two-step perturbation method^36^ by eliminating the interaction between the SDA and framework. The VDW and electrostatic interactions were decoupled sequentially, therefore, the entire calculation took 4 steps. Each step ran for 50 ps equilibration and 50 ps data collection.

## Additional Information

**How to cite this article**: Xin, L. *et al.* Origin of the structure-directing effect resulting in identical topological open-framework materials. *Sci. Rep.* **5**, 14940; doi: 10.1038/srep14940 (2015).

## Supplementary Material

Supplementary Information

Supplementary Material

Supplementary Data

## Figures and Tables

**Table 1:** Crystallographic data for “one-structure/multiple-template” systems.

**Structural formula**	**Structure-directing agents**	**Space group**	**a (Å)**	**b (Å)**	**c (Å)**	**α (°)**	**β (°)**	**γ (°)**
**I**—**Aluminophosphates with chabazite (CHA) topology.**								
Al_3_P_3_O_12_·F·C_4_H_10_NO	morpholine^9^	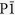	9.333	9.183	9.162	88.45	102.57	93.76
Al_3_P_3_O_12_·F·C_5_H_5_NH·0.15H_2_O	pyridine^10^	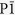	9.118	9.161	9.335	85.98	77.45	89.01
Al_3_P_3_O_12_·F·C_5_H_10_NH_2_·0.25H_2_O	piperidine^11^	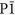	9.1800	9.1957	9.3606	85.532	78.192	87.739
Al_3_P_3_O_12_·F·C_3_H_7_NH_3_·H_2_O	isopropylamine^11^	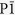	9.1231	9.2411	9.3426	86.769	79.946	87.846
Al_3_P_3_O_12_·F·(C_2_H_5_)_2_NH_2_·0.5H_2_O	diethylamine^11^	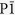	9.199	9.202	9.295	87.525	79.027	87.884
**II**—**Layered aluminophosphates with 4** × **6** × **8 network topology.**								
Al_3_P_4_O_16_·(CH_3_)_2_NH(CH_2_)_2_NH(CH_3_)_2_·H_3_O	tetramethylethylenediamine^12^	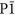	8.9907	9.8359	14.5566	75.872	88.616	63.404
Al_3_P_4_O_16_·(C_4_H_7_NH_3_)_2_·C_5_H_10_NH_2_	cyclobutylamine, piperidine^13^	P2_1_	8.993	14.884	9.799	90	103.52	90
Al_3_P_4_O_16_·(CH_3_CH_2_NH_3_)_3_	ethylamine^14^	P2_1_/m	8.920	14.896	9.363	90	106.07	90
Al_3_P_4_O_16_·H_3_N(CH_2_)_5_NH_3_·C_5_H_10_NH_2_	1,5-diaminopentane^15^	P2_1_/c	9.801	14.837	17.815	90	105.65	90
Al_3_P_4_O_16_·(CH_3_CH_2_CH_2_NH_3_)_3_	*n*-propylamine^16^	P2_1_/n	11.310	14.854	14.796	90	93.64	90
**III**—**Microporous gallophosphates with ULM-3 topology.**								
Ga_3_P_3_O_12_·F_2_·H_3_N(CH_2_)_3_NH_3_·H_2_O	1,3-diaminopropane^17^	Pbca	10.154	18.393	15.773	90	90	90
Ga_3_P_3_O_12_·F_2_·H_3_N(CH_2_)_4_NH_3_	1,4-diaminobutane^18^	Pbca	10.075	18.506	16.060	90	90	90
Ga_3_P_3_O_12_·F_2_·H_3_N(CH_2_)_5_NH_3_	1,5-diaminopentane^18^	Pbca	10.156	18.672	16.367	90	90	90
**IV**—**Layered zinc phosphates.**								
Zn_2_(PO_4_)(HPO_4_)(H_2_PO_4_)·C_4_H_14_N_2_	3-methylaminopropylamine^19^	Pn	11.8920	5.1318	12.3063	90	98.125	90
Zn_2_(H_0.5_PO_4_)_2_(H_2_PO_4_)·C_4_N_2_H_14_	N,N’-dimethylethylenediamine^20^	P2/n	11.7877	5.2093	12.2031	90	98.198	90

**Table 2:** Unit cell (framework) volume (*V*_FM_), framework energy (*E*_FM_), binding free energy calculated through free energy perturbation (*F*_B_), total atomic charge on SDA (*Q*_SDA_) and total formal charge on SDA (*FC*_SDA_).

**Topology**	**Structure-directing agents**	***V***_**FM**_	***E***_**FM**_	***F***_**B**_	***Q***_**SDA**_	***FC***_**SDA**_
I	morpholine	55.9	−137.7	−4.8	0.490	2
	pyridine	53.2	−139.3	−3.2	0.538	2
	piperidine	57.1	−135.1	−3.8	0.698	2
	isopropylamine	58.5	−138.9	−3.0	0.388	2
	diethylamine	59.9	−136.2	−5.0	0.315	2
	Average	56.9	−137.4	−4.0	0.485	
	(STD)	(2.6)	(1.8)	(0.9)	(0.14)	
II	tetramethylethylenediamine	84.7	−126.0	−6.7	1.079	3
	cyclobutylamine, piperidine	113.6	−118.5	−10.0	1.609	3
	ethylamine	82.0	−123.6	−6.5	1.059	3
	1,5-diaminopentane	100.0	−121.7	−7.4	1.282	3
	*n*-propylamine	102.0	−114.5	−4.5	1.647	3
	Average	96.5	−120.9	−7.0	1.335	
	(STD)	(13.1)	(4.5)	(2.0)	(0.281)	
III	1,3-diaminopropane	51.5	−383.6	−3.7	0.670	2
	1,4-diaminobutane	53.2	−379.1	−3.0	0.604	2
	1,5-diaminopentane	55.9	−347.3	−3.6	0.656	2
	Average	53.5	−370.0	−3.4	0.644	
	(STD)	(2.2)	(19.7)	(0.4)	(0.034)	
IV	3-methylaminopropylamine	79.4	−57.3	−12.2	0.904	2
	N,N’-dimethylethylenediamine	80.6	−52.3	−13.6	1.055	2
	Average	80.0	−54.8	−12.9	0.980	
	(STD)	(0.8)	(3.5)	(1.0)	(0.107)	

The volumes and energies were normalized by the number of tetrahedral centers. The volume is in Å^3^; energies are in kcal/mol; charges are in electrons. Topologies were denoted by Roman numerals as in Table 1.
