# Supplementary material for: Origin of the structure-directing effect resulting in identical topological open-framework materials
Source: Sci Rep. 2015 Oct 8;5:14940. doi: 10.1038/srep14940 (PMC4597409; doi:10.1038/srep14940)
Supplement: Supplementary Information [file srep14940-s1.pdf]

Supplementary Information

Origin of the structure-directing effect resulting in identical topological open-framework materials

Liang Xin[1,2], Huai Sun[2], Ruren Xu[1] & Wenfu Yan[1]*

[1] State Key Laboratory of Inorganic Synthesis and Preparative Chemistry, College of Chemistry, Jilin University, 2699 Qianjin Street, Changchun 130012, PR China. [2] College of Chemistry and Chemical Engineering, Shanghai Jiaotong University, Shanghai 200240, PR China

Correspondence and requests for materials should be addressed to W.Y. (email: yanw@jlu.edu.cn).

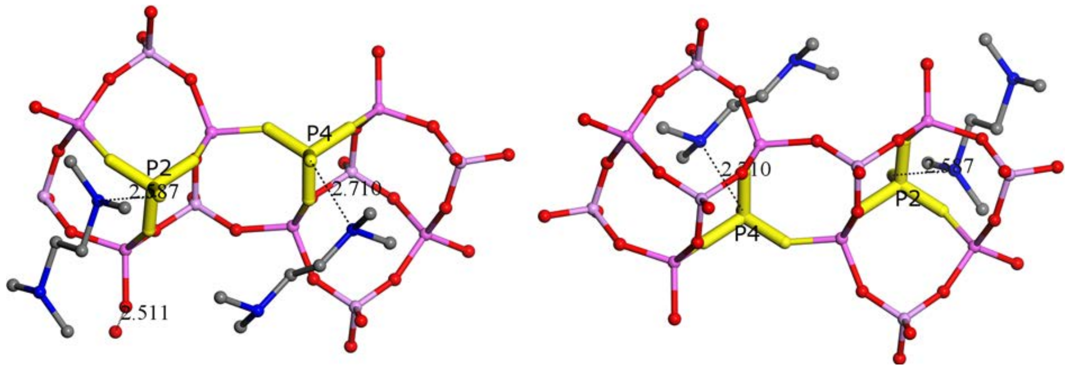
P2
P4
2.587
2.710
2.511
P2
P4

(a)

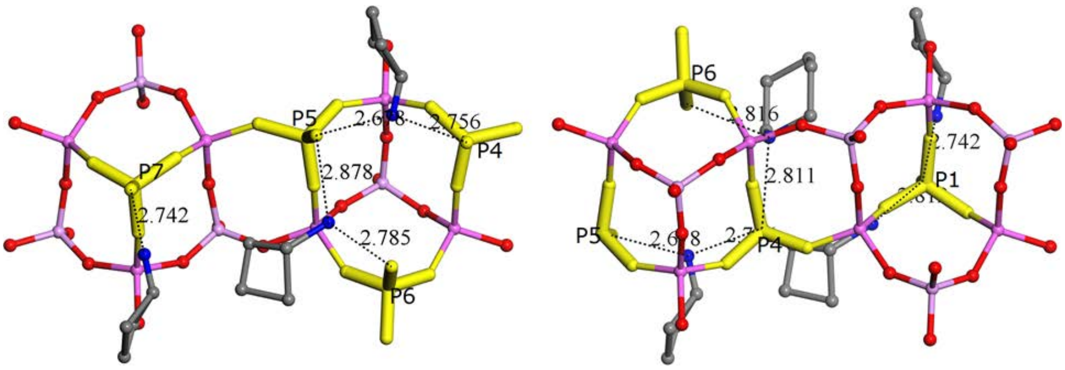
P5
P4
P7
P6
2.756
2.878
2.742
2.785
P6
P1
P5
P4
2.816
2.811
2.742

(b)

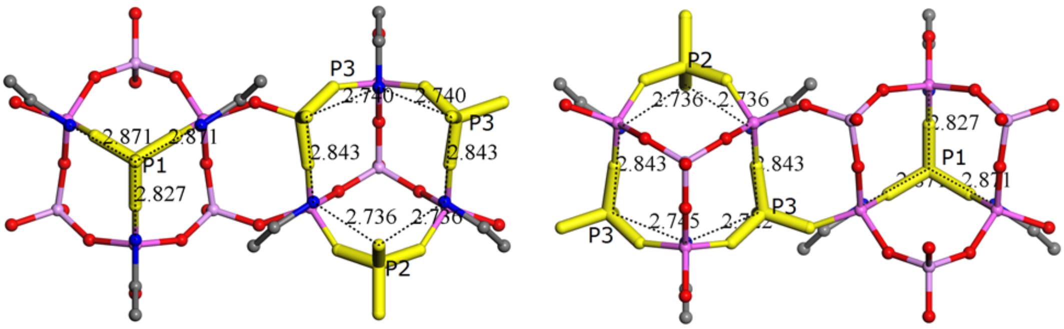
P3
P1
P3
P2
2.871
2.827
2.740
2.843
2.736
P2
P1
P3
P3
2.736
2.827
2.843
2.745

(c)

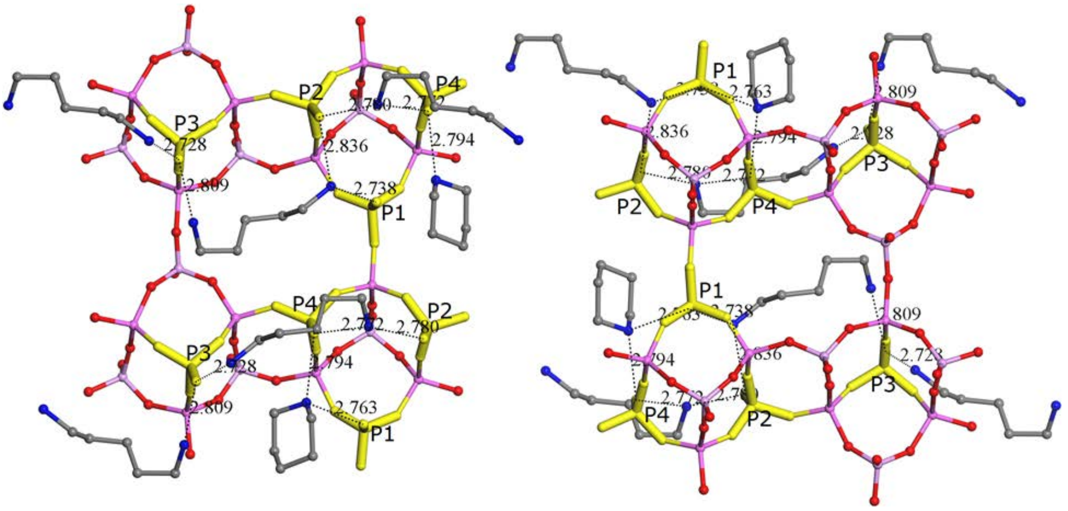
P3
P2
P4
P1
2.728
2.809
2.836
2.794
2.738
P4
P2
P3
P1
2.780
2.763
P1
P2
P3
P4
2.763
2.836
2.794
2.809
2.738
2.723

(d)

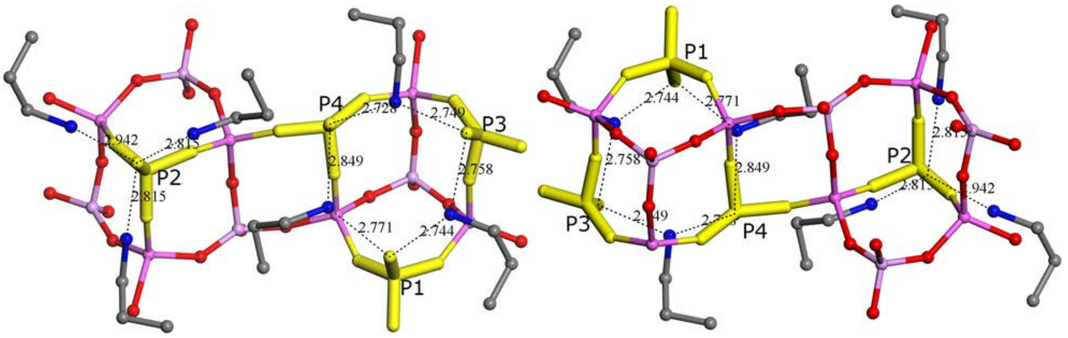

(e)

Supplementary Figure S1 | The repeating capped six-ring units and near non-framework species, as well as the highlighted core units with a close contact of 3.0 Å for layered aluminophosphates with 4×6×8 network topology. The repeat units were placed with very similar orientations. The structure-directing agents were (**a**) tetramethylethylenediamine (Left: above layer; Right: below layer), (**b**) cyclobutylamine and piperidine (Left: abo ve layer; Right: below layer), (**c**) ethylamine (Left: above layer; Right: below layer), (**d**) 1,5-diaminopentane (Left: above layer; Right: below layer), and (**e**) *n*-propylamine (Left: above layer; Right: below layer). Key Al and P atoms are labeled with their names. Oxygen, nitrogen, and carbon atoms are labeled with red, blue, and grey colors, respectively.

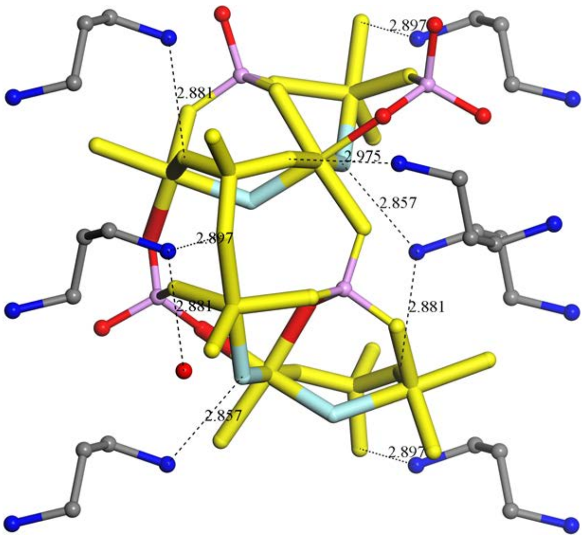

(a)

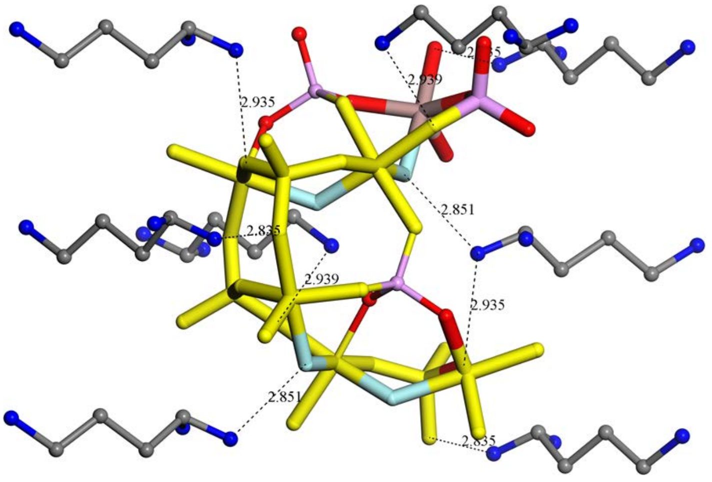

(b)

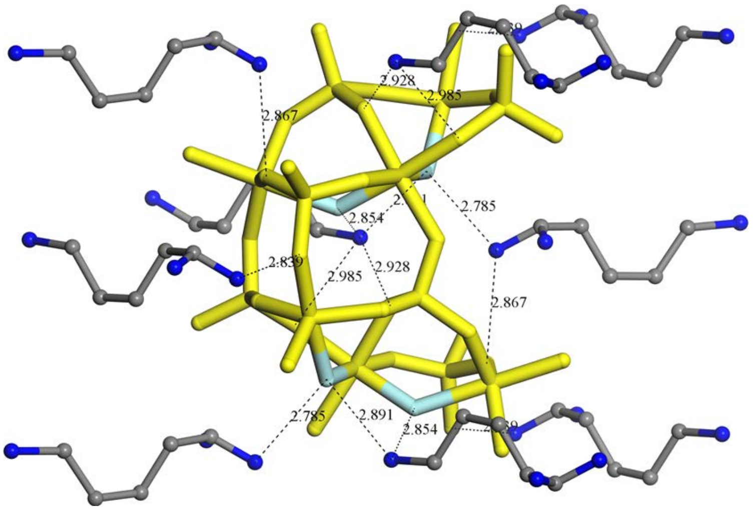

(c)

Supplementary Figure S2 | The repeat units and near non-framework species, as well as the highlighted core units with a close contact of 3.0 Å for microporous gallophosphates with

ULM-3 topology. The repeat units were placed with very similar orientations. The structure-directing agents were (**a**) 1,3-diaminopropane, (**b**) 1,4-diaminobutane, and (**c**) 1,5-diaminopentane. Center atoms of the tetrahedron and octahedron were P and Ga, respectively. Oxygen, nitrogen, fluorine, and carbon atoms are labeled with red, blue, cyan, and grey colors, respectively.

Supplementary Table S1 | Synthesis conditions for “one-structure/multiple-template” systems

I -Aluminophosphates with chabasite (CHA) topology.

Structural formula	Structure-directing agents	Gel composition (mol)	Temp. (°C)	Time (day)
$Al_3P_3O_{12}\cdot F\cdot C_4H_{10}NO$	morpholine	$A1_2O_3:P_2O_5:1.5C_4H_{10}NO:HF:100H_2O$	200	10
$Al_3P_3O_{12}\cdot F\cdot C_5H_5NH\cdot 0.15H_2O$	pyridine	$0.9Al_2O_3\cdot 2.5H_2O:1.8P_2O_5$:8.0 Py:1.0HF-Py:14TEG	180	2-6
$Al_3P_3O_{12}\cdot F\cdot C_5H_{10}NH_2\cdot 0.25H_2O$	piperidine	$Al(OPr^i)_3:2.6H_3PO_4:6.5C_5H_{10}NH$:18 tEG:0.1HF	180	12
$Al_3P_3O_{12}\cdot F\cdot C_3H_7NH_3\cdot H_2O$	iso-propylamine	$Al(OPr^i)_3:2.9H_3PO_4$:(5.0~6.0)i-PrNH_2:50 EG:0.1HF	180	10
$Al_3P_3O_{12}\cdot F\cdot (C_2H_5)_2NH_2\cdot 0.5H_2O$	diethylamine	$Al(OPr^i)_3:7.0H_3PO_4:6.0(C_2H_5)_2NH$:15tEG: 0.1HF	180	10

II -Layered aluminophosphates with 4×6×8 network topology.

Structural formula	Structure-directing agents	Gel composition (mol)	Temp. (°C)	Time (day)
$Al_3P_4O_{16}\cdot (CH_3)_2NH(CH_2)_2NH(CH_3)_2\cdot H_3O$	tetramethylethylenediamine	$Al(OPr^i)_3:(2.8\sim3.3)H_3PO_4:(3.8\sim5.0)$TMEDA:15tEG	180	8
$Al_3P_4O_{16}\cdot (C_4H_7NH_3)_2\cdot C_5H_{10}NH_2$	cyclobutylamine, piperidine	$0.9Al_2O_3\cdot nH_2O:1.8P_2O_5$:2.9cyclobutylamine:2.9piperidine:14TEG	180	6
$Al_3P_4O_{16}\cdot (CH_3CH_2NH_3)_3$	ethylamine	$Al(OPr^i)_3:3.0H_3PO_4:14.7EtNH_2$:34.5BuOH:27.4EG	180	13
$Al_3P_4O_{16}\cdot H_3N(CH_2)_5NH_3\cdot C_5H_{10}NH_2$	1,5-diaminopentane	$Al_2O_3:P_2O_5:1.2\ H_2N(CH_2)_5NH_2$:40tEG:0.3HF	190	6
$Al_3P_4O_{16}\cdot (CH_3CH_2CH_2NH_3)_3$	*n*-propylamine	$Al(OPr^i)_3:2.4H_3PO_4:5.0n$-Pr$NH_2$:50Bu^{s}OH	180	9

III - Microporous gallophosphates with ULM-3 topology.

Structural formula	Structure-directing agents	Gel composition (mol)	Temp. (°C)	Time (day)
$Ga_3P_3O_{12}\cdot F_2\cdot H_3N(CH_2)_3NH_3\cdot H_2O$	1,3-diaminopropane	$Ga_2O_3:P_2O_5:2.0HF:1.3H_2N(CH_2)_3NH_2:80H_2O$	180	1
$Ga_3P_3O_{12}\cdot F_2\cdot H_3N(CH_2)_4NH_3$	1,4-diaminobutane	$Ga_2O_3:P_2O_5:2.0HF:H_2N(CH_2)_4NH_2:80H_2O$	180	1
$Ga_3P_3O_{12}\cdot F_2\cdot H_3N(CH_2)_5NH_3$	1,5-diaminopentane	$Ga_2O_3:2.0P_2O_5:2.0HF:1.2H_2N(CH_2)_5NH_2:80H_2O$	180	1

IV -Layered zinc phosphates.

Structural formula	Structure-directing agents	Gel composition (mol)	Temp. (°C)	Time (day)
$Zn_2(PO_4)(HPO_4)(H_2PO_4)\cdot C_4H_{14}N_2$	3-methylaminopropylamine	$Zn(OAc)_2:5.5H_3PO_4$:2MPA:100H_2O	164	2
$Zn_2(H_{0.5}PO_4)_2(H_2PO_4)\cdot C_4N_2H_{14}$	N,N’-dimethylethylenediamine	$Zn(OAc)_2:3.05H_3PO_4$:2.25dmen:138H_2O	60	7

Note: $Al(OPr^i)_3$= aluminum triisopropoxide; tEG= triethylene glycol; Py=pyridine; TEG= tetraethyleneglycol; EG=ethylene glycol; TMEDA= tetramethylethylenediamine; BuOH= *n*-butanol; Bu^{s}OH= butan-2-ol; MPA=3-methylaminopropylamine; dmen= N,N’-dimethylethylenediamine

Supplementary Table S2 | Crystallographic data and structure refinement for $AlPO_4$-CHA-piperidine.

Identification code	$AlPO_4$-CHA-piperidine
Empirical formula	C5H12.50Al3FNO12.25P3
Formula weight	475.51
Temperature	293(2) K
Wavelength	0.71073 Å
Crystal system, space group	Triclinic, $P\bar{1}$
Unit cell dimensions	a = 9.1800(8) Å α = 86.532(2) º
	b = 9.1957(8) Å β = 78.192(2) º
	c = 9.3606(8) Å γ = 87.739(2) º
Volume	771.76(12) $Å^3$
Z, Calculated density	2, 2.046 Mg/m^3
Absorption coefficient	0.635 mm^{-1}
F(000)	481
Crystal size	0.5 × 0.3 × 0.1 mm^3
Theta range for data collection	2.22 to 23.25 º
Limiting indices	$-10\leq h\leq 9$, $-9\leq k\leq 10$, $-10\leq l\leq 10$
Reflections collected / unique	3821 / 2206 [R(int) = 0.1145]
Completeness to theta = 23.25	99.6 %
Refinement method	Full-matrix least-squares on F^2
Data / restraints / parameters	2206 / 0 / 235
Goodness-of-fit on F^2	1.037
Final R indices [I>2σ(I)]*	R1 = 0.0493, wR2 = 0.1152
R indices (all data)	R1 = 0.0675, wR2 = 0.1232
Largest diff. peak and hole	0.641 and -0.704 $eÅ^{-3}$

* $R1=\sum(\Delta F/\sum(F_o))$; $wR2=(\sum[w(F_o^2-F_c^2)])/\sum[w(F_o^2)^2]^{1/2}$, $w=1/\sigma^2(F_o^2)$

Supplementary Table S3 | Crystallographic data and structure refinement for $AlPO_4$-CHA-iso-propylamine.

Identification code	$AlPO_4$-CHA-iso-propylamine	
Empirical formula	C3H12Al3FNO13P3	
Formula weight	462.99	
Temperature	293(2) K	
Wavelength	0.71073 Å	
Crystal system, space group	Triclinic, $P\bar{1}$	
Unit cell dimensions	a = 9.1231(14) Å	α= 86.769(4) º
	b = 9.2411(14) Å	β= 79.946(4) º
	c = 9.3426(15) Å	γ = 87.846(4) º
Volume	774.0(2) $Å^3$	
Z, Calculated density	2, 1.987 Mg/m^3	
Absorption coefficient	0.634 mm^{-1}	
F(000)	468	
Crystal size	0.08 × 0.08 × 0.08 mm	
Theta range for data collection	2.21 to 23.23 º	
Limiting indices	-10≤h≤10, -10≤k≤8, -10≤l≤10	
Reflections collected / unique	3826 / 2207 [R(int) = 0.1002]	
Completeness to theta=23.23	99.6 %	
Absorption correction	Empirical	
Max. and min. transmission	0.326 and 0.228	
Refinement method	Full-matrix least-squares on F^2	
Data / restraints / parameters	2207 / 0 / 217	
Goodness-of-fit on F^2	0.960	
Final R indices [I>2σ(I)]*	R_1 = 0.0560, wR_2 = 0.1298	
R indices (all data)	R_1= 0.0933, wR_2= 0.1453	
Largest diff. peak and hole	0.606 and -0.628 e. $Å^{-3}$	

* $R1=\sum(\Delta F/\sum(F_o))$; $wR2=(\sum[w(F_o^2-F_c^2)])/\sum[w(F_o^2)^2]^{1/2}$, $w=1/\sigma^2(F_o^2)$

Supplementary Table S4 | Crystallographic data and structure refinement for $AlPO_4$-CHA-diethylamine.

Identification code	$AlPO_4$-CHA-diethylamine	
Empirical formula	C4 H12 Al3 F N O12.50 P3	
Formula weight	467.00	
Temperature	293(2) K	
Wavelength	0.71073 Å	
Crystal system, space group	Triclinic, $P\bar{1}$	
Unit cell dimensions	a = 9.199(4) Å	α = 87.525(8) º
	b = 9.202(4) Å	β = 79.027(8) º
	c = 9.295(4) Å	γ = 87.884(7) º
Volume	771.4(6) $Å^3$	
Z, Calculated density	2, 2.011 Mg/m^3	
Absorption coefficient	0.635 mm^{-1}	
F(000)	472	
Crystal size	0.2×0.4×0.6 mm	
Theta range for data collection	2.22 to 27.52 º	
Limiting indices	$-11\leq h\leq 11$, $-11\leq k\leq 11$, $-12\leq l\leq 11$	
Reflections collected / unique	4615 / 3256 [R(int) = 0.0484]	
Completeness to theta = 27.52	91.6 %	
Refinement method	Full-matrix least-squares on F^2	
Data / restraints / parameters	3256 / 0 / 226	
Goodness-of-fit on F^2	1.116	
Final R indices [I>2σ(I)]*	R_1 = 0.0537, wR_2 = 0.1595	
R indices (all data)	R_1 = 0.0641, wR_2 = 0.1853	
Largest diff. peak and hole	1.618 and -1.275 e. $Å^{-3}$	

* $R1=\sum(\Delta F/\sum(F_o))$; $wR2=(\sum[w(F_o^2-F_c^2)])/\sum[w(F_o^2)^2]^{1/2}$, $w=1/\sigma^2(F_o^2)$

Supplementary Table S5 | Atomic coordinates ($\times10^4$) and equivalent isotropic displacement parameters ($Å^2\times10^3$) for $AlPO_4$-CHA-piperidine. U(eq) is defined as one third of the trace of the orthogonalized U_{ij} tensor.

	x	y	z	U(eq)
P(1)	1151(1)	3138(1)	6029(1)	12(1)
P(2)	1321(1)	-1184(1)	8218(1)	12(1)
P(3)	3317(1)	-1478(2)	3596(1)	13(1)
Al(1)	3665(2)	613(2)	5887(2)	12(1)
Al(2)	-1032(2)	1297(2)	8422(2)	13(1)
Al(3)	1351(2)	-3410(2)	5757(2)	13(1)
F(1)	5077(3)	1070(3)	4195(3)	16(1)
O(1)	1426(4)	4738(4)	6176(4)	21(1)
O(2)	2827(4)	-2983(4)	4322(4)	18(1)
O(3)	-219(4)	-459(4)	8307(4)	21(1)
O(4)	1472(4)	-1726(4)	9761(4)	19(1)
O(5)	2682(4)	-1274(4)	2194(4)	20(1)
O(6)	351(4)	2989(4)	4755(4)	23(1)
O(7)	4995(4)	-1450(4)	3125(3)	16(1)
O(8)	144(4)	2587(4)	7461(4)	21(1)
O(9)	2608(4)	2320(4)	5750(3)	16(1)
O(10)	2662(3)	-312(4)	4645(4)	17(1)
O(11)	1402(4)	-2530(4)	7332(3)	18(1)
O(12)	2513(4)	-120(4)	7604(4)	17(1)
N(1)	2819(11)	4510(11)	1681(10)	108(3)
C(3)	3553(12)	1997(10)	19(13)	82(3)
C(4)	2824(10)	3219(15)	-540(10)	93(4)
C(1)	3518(11)	3060(20)	2225(9)	125(6)
C(5)	2147(16)	4285(10)	434(14)	127(5)
C(2)	4285(17)	2248(14)	1070(20)	149(7)
O(1W)	4890(20)	-4620(20)	6020(30)	75(7)

Supplementary Table S6 | Atomic coordinates ($\times 10^4$) and equivalent isotropic displacement parameters ($Å^2 \times 10^3$) for $AlPO_4$-CHA-iso-propylamine. U(eq) is defined as one third of the trace of the orthogonalized U_{ij} tensor.

	x	y	z	U(eq)
Al(1)	3574(2)	3472(2)	9242(2)	14(1)
Al(2)	1334(2)	-578(2)	9095(2)	14(1)
Al(3)	5989(2)	-1335(2)	6575(2)	16(1)
P(1)	3680(2)	1182(2)	6811(2)	14(1)
P(2)	3841(2)	-3142(2)	8904(2)	14(1)
P(3)	-1670(2)	-1559(2)	8624(2)	14(1)
O(1)	3639(5)	2535(5)	7679(5)	20(1)
O(2)	2441(5)	167(5)	7404(5)	19(1)
O(3)	3560(5)	1694(5)	5255(4)	18(1)
O(4)	5197(5)	403(5)	6806(5)	23(1)
O(5)	3454(5)	-4713(5)	8750(5)	21(1)
O(6)	4766(5)	-3103(5)	10115(5)	26(1)
O(7)	4774(5)	-2615(5)	7462(5)	22(1)
O(8)	2442(5)	-2245(5)	9297(5)	18(1)
O(9)	-2086(5)	-3048(5)	9404(5)	21(1)
O(10)	-2367(5)	-1418(5)	7248(5)	22(1)
O(11)	-2338(5)	-389(5)	9652(5)	18(1)
O(12)	8(5)	-1480(5)	8134(5)	17(1)
OW1	848(18)	8778(16)	5133(12)	211(7)
F(1)	-21(4)	-1056(4)	10820(4)	17(1)
C(1)	420(20)	5740(19)	2920(30)	226(12)
C(2)	2016(18)	6060(14)	2891(16)	112(5)
C(3)	2729(17)	5645(18)	4211(19)	151(8)
N(1)	2177(9)	7662(9)	2615(8)	54(2)

Supplementary Table S7 | Atomic coordinates (×10^4) and equivalent isotropic displacement parameters (Å^2×10^3) for $AlPO_4$-CHA-diethylamine. U(eq) is defined as one third of the trace of the orthogonalized U_{ij} tensor.

	x	y	z	U(eq)
Al(1)	5996(1)	-1332(1)	1599(1)	12(1)
Al(2)	6371(1)	-3431(1)	5735(1)	11(1)
Al(3)	8667(1)	617(1)	5915(1)	11(1)
P(1)	6341(1)	-1153(1)	8185(1)	11(1)
P(2)	6155(1)	3155(1)	6065(1)	11(1)
P(3)	8320(1)	-1525(1)	3601(1)	11(1)
F(1)	9961(2)	-1056(2)	5819(2)	14(1)
O(1)	4839(3)	-389(3)	8183(4)	21(1)
O(2)	6488(3)	4740(3)	6209(3)	19(1)
O(3)	6415(4)	-1680(3)	9748(3)	19(1)
O(4)	5146(3)	2658(3)	7486(3)	21(1)
O(5)	7658(3)	-1330(3)	2195(3)	19(1)
O(6)	5328(3)	3046(3)	4786(3)	21(1)
O(7)	7837(3)	-3021(3)	4312(3)	17(1)
O(8)	6415(3)	-2513(3)	7300(3)	16(1)
O(9)	9991(3)	-1492(3)	3126(3)	14(1)
O(10)	7582(3)	-143(3)	7629(3)	18(1)
O(11)	7677(3)	-350(3)	4665(3)	16(1)
O(12)	7580(3)	2284(3)	5765(3)	18(1)
N(1)	8233(14)	2521(11)	2064(13)	126(4)
C(1)	8890(40)	2155(17)	1060(30)	300(20)
C(2)	7491(19)	3100(20)	23(15)	146(7)
C(3)	8800(40)	4030(20)	2576(16)	229(15)
C(4)	10193(13)	4080(30)	2220(40)	310(20)
O(1W)	9750(20)	-150(30)	-327(19)	141(8)

Supplementary Table S8 | Bond lengths [Å] and angles [º] for $AlPO_4$-CHA-piperidine.

P(1)-O(9)	1.491(4)	P(1)-O(1)	1.522(4)
P(1)-O(8)	1.535(4)	P(1)-O(6)	1.539(3)
P(2)-O(12)	1.498(4)	P(2)-O(11)	1.522(4)
P(2)-O(3)	1.528(4)	P(2)-O(4)	1.531(3)
P(3)-O(7)	1.514(3)	P(3)-O(10)	1.520(4)
P(3)-O(5)	1.540(3)	P(3)-O(2)	1.546(4)
Al(1)-O(9)	1.825(4)	Al(1)-O(12)	1.840(4)
Al(1)-F(1)	1.868(3)	Al(1)-O(10)	1.883(3)
Al(1)-F(1)#1	1.889(3)	Al(1)-O(7)#1	1.893(3)
Al(1)-Al(1)#1	2.894(3)	Al(2)-O(8)	1.719(4)
Al(2)-O(5)#2	1.729(3)	Al(2)-O(4)#3	1.732(3)
Al(2)-O(3)	1.751(4)	Al(3)-O(1)#4	1.726(4)
Al(3)-O(11)	1.734(3)	Al(3)-O(2)	1.740(4)
Al(3)-O(6)#2	1.748(4)	F(1)-Al(1)#1	1.889(3)
O(1)-Al(3)#5	1.726(4)	O(4)-Al(2)#3	1.732(3)
O(5)-Al(2)#2	1.729(3)	O(6)-Al(3)#2	1.748(4)
O(7)-Al(1)#1	1.893(3)	N(1)-C(5)	1.456(12)
N(1)-C(1)	1.560(16)	C(3)-C(2)	1.334(16)
C(3)-C(4)	1.414(15)	C(4)-C(5)	1.416(15)
C(1)-C(2)	1.404(19)	O(9)-P(1)-O(1)	109.0(2)
O(9)-P(1)-O(8)	111.6(2)	O(1)-P(1)-O(8)	107.3(2)
O(9)-P(1)-O(6)	110.0(2)	O(1)-P(1)-O(6)	109.6(2)
O(8)-P(1)-O(6)	109.31(19)	O(12)-P(2)-O(11)	113.38(19)
O(12)-P(2)-O(3)	110.5(2)	O(11)-P(2)-O(3)	108.24(19)
O(12)-P(2)-O(4)	109.68(19)	O(11)-P(2)-O(4)	106.6(2)
O(3)-P(2)-O(4)	108.3(2)	O(7)-P(3)-O(10)	113.86(19)
O(7)-P(3)-O(5)	106.76(19)	O(10)-P(3)-O(5)	109.9(2)
O(7)-P(3)-O(2)	110.51(19)	O(10)-P(3)-O(2)	108.5(2)
O(5)-P(3)-O(2)	107.18(19)	O(9)-Al(1)-O(12)	97.02(16)
O(9)-Al(1)-F(1)	93.45(15)	O(12)-Al(1)-F(1)	169.37(17)
O(9)-Al(1)-O(10)	92.74(15)	O(12)-Al(1)-O(10)	95.75(15)
F(1)-Al(1)-O(10)	85.51(14)	O(9)-Al(1)-F(1)#1	172.68(16)
O(12)-Al(1)-F(1)#1	90.30(15)	F(1)-Al(1)-F(1)#1	79.23(14)
O(10)-Al(1)-F(1)#1	86.59(14)	O(9)-Al(1)-O(7)#1	93.19(16)
O(12)-Al(1)-O(7)#1	92.59(15)	F(1)-Al(1)-O(7)#1	85.02(14)
O(10)-Al(1)-O(7)#1	169.10(17)	F(1)#1-Al(1)-O(7)#1	86.36(14)

Supplementary Table S8 | Bond lengths [Å] and angles [º] for $AlPO_4$-CHA-piperidine. (continued)

O(9)-Al(1)-Al(1)#1	133.34(14)	O(12)-Al(1)-Al(1)#1	129.61(14)
F(1)-Al(1)-Al(1)#1	39.89(9)	O(10)-Al(1)-Al(1)#1	84.87(12)
F(1)#1-Al(1)-Al(1)#1	39.34(9)	O(7)#1-Al(1)-Al(1)#1	84.41(12)
O(8)-Al(2)-O(5)#2	111.78(18)	O(8)-Al(2)-O(4)#3	108.56(18)
O(5)#2-Al(2)-O(4)#3	107.39(17)	O(8)-Al(2)-O(3)	112.01(19)
O(5)#2-Al(2)-O(3)	108.03(17)	O(4)#3-Al(2)-O(3)	108.92(18)
O(1)#4-Al(3)-O(11)	107.62(17)	O(1)#4-Al(3)-O(2)	108.56(18)
O(11)-Al(3)-O(2)	113.20(17)	O(1)#4-Al(3)-O(6)#2	107.83(18)
O(11)-Al(3)-O(6)#2	108.85(18)	O(2)-Al(3)-O(6)#2	110.60(18)
Al(1)-F(1)-Al(1)#1	100.77(14)	P(1)-O(1)-Al(3)#5	156.3(2)
P(3)-O(2)-Al(3)	129.4(2)	P(2)-O(3)-Al(2)	138.4(2)
P(2)-O(4)-Al(2)#3	143.3(2)	P(3)-O(5)-Al(2)#2	141.9(2)
P(1)-O(6)-Al(3)#2	141.1(3)	P(3)-O(7)-Al(1)#1	126.8(2)
P(1)-O(8)-Al(2)	149.7(2)	P(1)-O(9)-Al(1)	149.2(2)
P(3)-O(10)-Al(1)	126.1(2)	P(2)-O(11)-Al(3)	153.3(2)
P(2)-O(12)-Al(1)	143.2(2)	C(5)-N(1)-C(1)	111.1(8)
C(2)-C(3)-C(4)	116.0(9)	C(3)-C(4)-C(5)	118.6(8)
C(2)-C(1)-N(1)	112.4(8)	C(4)-C(5)-N(1)	117.4(9)
C(3)-C(2)-C(1)	116.8(12)		

Symmetry transformations used to generate equivalent atoms:
#1 -x+1,-y,-z+1 #2 -x,-y,-z+1 #3 -x,-y,-z+2
#4 x,y-1,z #5 x,y+1,z

Supplementary Table S9 | Bond lengths [Å] and angles [º] for $AlPO_4$-CHA-iso-propylamine.

Al(1)-O(5)#1	1.719(5)	Al(1)-O(9)#2	1.727(5)
Al(1)-O(1)	1.730(5)	Al(1)-O(6)#3	1.739(5)
Al(2)-O(8)	1.829(5)	Al(2)-O(2)	1.834(5)
Al(2)-O(12)	1.873(5)	Al(2)-O(11)#2	1.887(5)
Al(2)-F(1)#2	1.889(4)	Al(2)-F(1)	1.894(4)
Al(3)-O(10)#4	1.722(5)	Al(3)-O(7)	1.727(5)
Al(3)-O(3)#5	1.735(5)	Al(3)-O(4)	1.745(5)
P(1)-O(2)	1.505(5)	P(1)-O(1)	1.524(5)
P(1)-O(3)	1.524(4)	P(1)-O(4)	1.536(5)
P(2)-O(8)	1.498(5)	P(2)-O(5)	1.527(5)
P(2)-O(7)	1.528(5)	P(2)-O(6)	1.528(5)
P(3)-O(12)	1.523(5)	P(3)-O(11)	1.525(5)
P(3)-O(10)	1.528(4)	P(3)-O(9)	1.549(5)
O(3)-Al(3)#5	1.735(5)	O(5)-Al(1)#6	1.719(5)
O(6)-Al(1)#3	1.739(5)	O(9)-Al(1)#2	1.727(5)
O(10)-Al(3)#7	1.722(5)	O(11)-Al(2)#2	1.887(5)
F(1)-Al(2)#2	1.889(4)	C(1)-C(2)	1.49(2)
C(2)-N(1)	1.497(14)	C(2)-C(3)	1.52(2)
O(5)#1-Al(1)-O(9)#2	108.9(2)	O(5)#1-Al(1)-O(1)	107.1(2)
O(9)#2-Al(1)-O(1)	114.0(2)	O(5)#1-Al(1)-O(6)#3	109.2(2)
O(9)#2-Al(1)-O(6)#3	109.7(2)	O(1)-Al(1)-O(6)#3	107.9(2)
O(8)-Al(2)-O(2)	98.1(2)	O(8)-Al(2)-O(12)	93.4(2)
O(2)-Al(2)-O(12)	93.8(2)	O(8)-Al(2)-O(11)#2	91.7(2)
O(2)-Al(2)-O(11)#2	95.5(2)	O(12)-Al(2)-O(11)#2	168.7(2)
O(8)-Al(2)-F(1)#2	170.9(2)	O(2)-Al(2)-F(1)#2	90.95(19)
O(12)-Al(2)-F(1)#2	87.08(19)	O(11)#2-Al(2)-F(1)#2	86.32(19)
O(8)-Al(2)-F(1)	91.82(19)	O(2)-Al(2)-F(1)	170.1(2)
O(12)-Al(2)-F(1)	85.53(19)	O(11)#2-Al(2)-F(1)	84.20(19)
F(1)#2-Al(2)-F(1)	79.15(18)	O(8)-Al(2)-Al(2)#2	131.33(19)
O(2)-Al(2)-Al(2)#2	130.59(19)	O(12)-Al(2)-Al(2)#2	85.20(16)
O(11)#2-Al(2)-Al(2)#2	83.84(17)	F(1)#2-Al(2)-Al(2)#2	39.64(12)
F(1)-Al(2)-Al(2)#2	39.51(12)	O(10)#4-Al(3)-O(7)	111.0(2)
O(10)#4-Al(3)-O(3)#5	106.7(2)	O(7)-Al(3)-O(3)#5	109.4(2)
O(10)#4-Al(3)-O(4)	108.3(2)	O(7)-Al(3)-O(4)	110.2(3)
O(3)#5-Al(3)-O(4)	111.2(2)	O(2)-P(1)-O(1)	113.1(3)
O(2)-P(1)-O(3)	109.7(2)	O(1)-P(1)-O(3)	106.9(3)

Supplementary Table S9 | Bond lengths [Å] and angles [º] for $AlPO_4$-CHA-iso-propylamine. (continued)

O(2)-P(1)-O(4)	110.2(3)	O(1)-P(1)-O(4)	108.0(3)
O(3)-P(1)-O(4)	108.7(3)	O(8)-P(2)-O(5)	109.8(3)
O(8)-P(2)-O(7)	112.4(3)	O(5)-P(2)-O(7)	107.4(3)
O(8)-P(2)-O(6)	109.1(3)	O(5)-P(2)-O(6)	108.6(3)
O(7)-P(2)-O(6)	109.4(3)	O(12)-P(3)-O(11)	114.2(3)
O(12)-P(3)-O(10)	106.8(3)	O(11)-P(3)-O(10)	109.5(3)
O(12)-P(3)-O(9)	110.6(3)	O(11)-P(3)-O(9)	107.5(3)
O(10)-P(3)-O(9)	108.1(3)	P(1)-O(1)-Al(1)	155.0(3)
P(1)-O(2)-Al(2)	143.3(3)	P(1)-O(3)-Al(3)#5	146.3(3)
P(1)-O(4)-Al(3)	138.6(3)	P(2)-O(5)-Al(1)#6	150.8(3)
P(2)-O(6)-Al(1)#3	150.1(4)	P(2)-O(7)-Al(3)	146.4(3)
P(2)-O(8)-Al(2)	150.9(3)	P(3)-O(9)-Al(1)#2	128.9(3)
P(3)-O(10)-Al(3)#7	145.2(3)	P(3)-O(11)-Al(2)#2	126.2(3)
P(3)-O(12)-Al(2)	126.1(3)	Al(2)#2-F(1)-Al(2)	100.85(18)
C(1)-C(2)-N(1)	107.4(13)	C(1)-C(2)-C(3)	119.7(14)
N(1)-C(2)-C(3)	106.5(13)		

Symmetry transformations used to generate equivalent atoms:
#1 x,y+1,z #2 -x,-y,-z+2 #3 -x+1,-y,-z+2
#4 x+1,y,z #5 -x+1,-y,-z+1 #6 x,y-1,z
#7 x-1,y,z

Supplementary Table S10 | Bond lengths [Å] and angles [°] for $AlPO_4$-CHA-diethylamine.

Al(1)-O(5)	1.723(3)	Al(1)-O(4)#1	1.726(3)
Al(1)-O(3)#2	1.731(3)	Al(1)-O(1)#1	1.737(3)
Al(2)-O(8)	1.720(3)	Al(2)-O(2)#3	1.725(3)
Al(2)-O(7)	1.740(3)	Al(2)-O(6)#1	1.742(3)
Al(3)-O(12)	1.815(3)	Al(3)-O(10)	1.837(3)
Al(3)-O(11)	1.876(3)	Al(3)-O(9)#4	1.877(3)
Al(3)-F(1)#4	1.887(3)	Al(3)-F(1)	1.906(3)
P(1)-O(10)	1.499(3)	P(1)-O(8)	1.520(3)
P(1)-O(3)	1.525(3)	P(1)-O(1)	1.527(3)
P(2)-O(12)	1.497(3)	P(2)-O(2)	1.518(3)
P(2)-O(4)	1.526(3)	P(2)-O(6)	1.537(3)
P(3)-O(9)	1.518(3)	P(3)-O(11)	1.523(3)
P(3)-O(5)	1.544(3)	P(3)-O(7)	1.544(3)
F(1)-Al(3)#4	1.887(3)	O(1)-Al(1)#1	1.737(3)
O(2)-Al(2)#5	1.725(3)	O(3)-Al(1)#6	1.731(3)
O(4)-Al(1)#1	1.726(3)	O(6)-Al(2)#1	1.742(3)
O(9)-Al(3)#4	1.877(3)	N(1)-C(3)	1.62(3)
O(5)-Al(1)-O(4)#1	111.69(16)	O(5)-Al(1)-O(3)#2	106.55(15)
O(4)#1-Al(1)-O(3)#2	109.49(16)	O(5)-Al(1)-O(1)#1	108.56(16)
O(4)#1-Al(1)-O(1)#1	111.48(16)	O(3)#2-Al(1)-O(1)#1	108.91(16)
O(8)-Al(2)-O(2)#3	106.27(15)	O(8)-Al(2)-O(7)	112.99(15)
O(2)#3-Al(2)-O(7)	108.77(15)	O(8)-Al(2)-O(6)#1	108.43(15)
O(2)#3-Al(2)-O(6)#1	109.02(16)	O(7)-Al(2)-O(6)#1	111.18(16)
O(12)-Al(3)-O(10)	98.50(15)	O(12)-Al(3)-O(11)	92.33(14)
O(10)-Al(3)-O(11)	95.78(14)	O(12)-Al(3)-O(9)#4	93.89(14)
O(10)-Al(3)-O(9)#4	93.36(13)	O(11)-Al(3)-O(9)#4	168.07(14)
O(12)-Al(3)-F(1)#4	93.31(13)	O(10)-Al(3)-F(1)#4	168.18(13)
O(11)-Al(3)-F(1)#4	84.34(13)	O(9)#4-Al(3)-F(1)#4	85.15(12)
O(12)-Al(3)-F(1)	172.24(14)	O(10)-Al(3)-F(1)	89.24(13)
O(11)-Al(3)-F(1)	86.25(12)	O(9)#4-Al(3)-F(1)	86.22(12)
F(1)#4-Al(3)-F(1)	78.97(11)	O(12)-Al(3)-Al(3)#4	133.02(12)
O(10)-Al(3)-Al(3)#4	128.49(12)	O(11)-Al(3)-Al(3)#4	83.90(11)
O(9)#4-Al(3)-Al(3)#4	84.41(10)	F(1)#4-Al(3)-Al(3)#4	39.72(7)
F(1)-Al(3)-Al(3)#4	39.25(7)	O(10)-P(1)-O(8)	113.32(17)
O(10)-P(1)-O(3)	109.52(17)	O(8)-P(1)-O(3)	106.08(17)
O(10)-P(1)-O(1)	110.99(18)	O(8)-P(1)-O(1)	107.95(17)

Supplementary Table S10 | Bond lengths [Å] and angles [º] for $AlPO_4$-CHA-diethylamine. (continued)

O(3)-P(1)-O(1)	108.77(19)	O(12)-P(2)-O(2)	109.20(17)
O(12)-P(2)-O(4)	112.46(17)	O(2)-P(2)-O(4)	107.49(17)
O(12)-P(2)-O(6)	109.86(17)	O(2)-P(2)-O(6)	108.66(18)
O(4)-P(2)-O(6)	109.07(18)	O(9)-P(3)-O(11)	113.76(16)
O(9)-P(3)-O(5)	106.90(16)	O(11)-P(3)-O(5)	109.58(17)
O(9)-P(3)-O(7)	110.93(16)	O(11)-P(3)-O(7)	108.47(17)
O(5)-P(3)-O(7)	106.98(17)	Al(3)#4-F(1)-Al(3)	101.03(11)
P(1)-O(1)-Al(1)#1	139.8(2)	P(2)-O(2)-Al(2)#5	152.7(2)
P(1)-O(3)-Al(1)#6	147.5(2)	P(2)-O(4)-Al(1)#1	147.3(2)
P(3)-O(5)-Al(1)	141.6(2)	P(2)-O(6)-Al(2)#1	142.8(2)
P(3)-O(7)-Al(2)	129.21(19)	P(1)-O(8)-Al(2)	153.8(2)
P(3)-O(9)-Al(3)#4	127.15(17)	P(1)-O(10)-Al(3)	141.41(19)
P(3)-O(11)-Al(3)	127.08(18)	P(2)-O(12)-Al(3)	151.8(2)
C(1)-N(1)-C(3)	112.6(19)	N(1)-C(1)-C(2)	89(2)
C(4)-C(3)-N(1)	110(2)		

Symmetry transformations used to generate equivalent atoms:
#1 -x+1,-y,-z+1 #2 x,y,z-1 #3 x,y-1,z
#4 -x+2,-y,-z+1 #5 x,y+1,z #6 x,y,z+1
#7 -x+2,-y,-z
