# Supplementary material for: Origin of the structure-directing effect resulting in identical topological open-framework materials
Source: Sci Rep. 2015 Oct 8;5:14940. doi: 10.1038/srep14940 (PMC4597409; doi:10.1038/srep14940)

checkCIF/PLATON report

You have not supplied any structure factors. As a result the full set of tests cannot be run.

THIS REPORT IS FOR GUIDANCE ONLY. IF USED AS PART OF A REVIEW PROCEDURE FOR PUBLICATION, IT SHOULD NOT REPLACE THE EXPERTISE OF AN EXPERIENCED CRYSTALLOGRAPHIC REFEREE.

No syntax errors found. CIF dictionary Interpreting this report

Datablock: yp-1

Bond precision: C-C = 0.0400 A Wavelength=0.71073

Cell:	a=9.199(4)	b=9.202(4)	c=9.295(4)
	alpha=87.525(8)	beta=79.027(8)	gamma=87.884(7)
Temperature:	293 K		

	Calculated	Reported
Volume	771.4(6)	771.4(6)
Space group	P -1	P-1
Hall group	-P 1	?
Moiety formula	2(Al3 F O12 P3), 2(C3 H9 N), 2(O0.50), 2(C H3)	?
Sum formula	C8 H24 Al6 F2 N2 O25 P6	C4 H12 Al3 F N O12.50 P3
Mr	933.99	467.00
Dx,g cm-3	2.011	2.011
Z	1	2
Mu (mm-1)	0.635	0.635
F000	472.0	472.0
F000’	473.26	
h,k,lmax	11,11,12	11,11,12
Nref	3555	3256
Tmin,Tmax		
Tmin’		

Correction method= Not given

Data completeness= 0.916 Theta(max)= 27.520

R(reflections)= 0.0537(2796) wR2(reflections)= 0.1853(3256)

S = 1.116 Npar= 226

The following ALERTS were generated. Each ALERT has the format
test-name_ALERT_alert-type_alert-level.
Click on the hyperlinks for more details of the test.

Alert level A

```
EXPT005_ALERT_1_A  _exptl_crystal_description is missing
            Crystal habit description.
            The following tests will not be performed.
            CRYSR_01
DIFF003_ALERT_1_A  _diffrn_measurement_device_type is missing
            Diffractometer make and type. Replaces _diffrn_measurement_type.
PLAT029_ALERT_3_A _diffrn_measured_fraction_theta_full Low .......      0.916 Note
PLAT245_ALERT_2_A U(iso) H1C     Smaller than U(eq) C1      by ...      0.220 AngSq
PLAT245_ALERT_2_A U(iso) H1D     Smaller than U(eq) C1      by ...      0.220 AngSq
PLAT245_ALERT_2_A U(iso) H3A     Smaller than U(eq) C3      by ...      0.150 AngSq
PLAT245_ALERT_2_A U(iso) H3B     Smaller than U(eq) C3      by ...      0.150 AngSq
PLAT245_ALERT_2_A U(iso) H4A     Smaller than U(eq) C4      by ...      0.231 AngSq
PLAT245_ALERT_2_A U(iso) H4B     Smaller than U(eq) C4      by ...      0.231 AngSq
PLAT245_ALERT_2_A U(iso) H4C     Smaller than U(eq) C4      by ...      0.231 AngSq
PLAT412_ALERT_2_A Short Intra XH3 .. XHn     H1D    ..  H4B     ..       1.59 Ang.
PLAT415_ALERT_2_A Short Inter D-H..H-X       H1B    ..  H2C     ..       1.73 Ang.
PLAT430_ALERT_2_A Short Inter D...A Contact  O1W    ..  O1W     ..       0.89 Ang.
```

Alert level B

```
DIFMX01_ALERT_2_B  The maximum difference density is > 0.1*ZMAX*1.00
            _refine_diff_density_max given =      1.618
            Test value =      1.500
PLAT097_ALERT_2_B Large Reported Max.  (Positive) Residual Density       1.62 eA-3
PLAT214_ALERT_2_B Atom C4      (Anion/Solvent) ADP max/min Ratio          5.1 prolat
PLAT245_ALERT_2_B U(iso) H2A     Smaller than U(eq) C2      by ...      0.066 AngSq
PLAT245_ALERT_2_B U(iso) H2B     Smaller than U(eq) C2      by ...      0.066 AngSq
PLAT245_ALERT_2_B U(iso) H2C     Smaller than U(eq) C2      by ...      0.066 AngSq
PLAT340_ALERT_3_B Low Bond Precision on  C-C Bonds ..............     0.0400 Ang.
PLAT360_ALERT_2_B Short  C(sp3)-C(sp3) Bond   C3     -   C4     ..       1.26 Ang.
PLAT414_ALERT_2_B Short Intra D-H..H-X       H1A    ..  H1C     ..       1.81 Ang.
PLAT415_ALERT_2_B Short Inter D-H..H-X       H1B    ..  H2B     ..       2.07 Ang.
```

Alert level C

```
DIFMN02_ALERT_2_C  The minimum difference density is < -0.1*ZMAX*0.75
            _refine_diff_density_min given =     -1.275
            Test value =     -1.125
DIFMN03_ALERT_1_C  The minimum difference density is < -0.1*ZMAX*0.75
            The relevant atom site should be identified.
DIFMX02_ALERT_1_C  The maximum difference density is > 0.1*ZMAX*0.75
            The relevant atom site should be identified.
PLAT052_ALERT_1_C Info on Absorption Correction Method Not Given .     Please Do !
PLAT053_ALERT_1_C Minimum Crystal Dimension Missing (or Error) ...     Please Check
PLAT054_ALERT_1_C Medium  Crystal Dimension Missing (or Error) ...     Please Check
PLAT055_ALERT_1_C Maximum Crystal Dimension Missing (or Error) ...     Please Check
PLAT098_ALERT_2_C Large Reported Min.  (Negative) Residual Density      -1.27 eA-3
PLAT244_ALERT_4_C Low   'Solvent' Ueq as Compared to Neighbors of         N1 Check
PLAT245_ALERT_2_C U(iso) H1A     Smaller than U(eq) N1      by ...      0.046 AngSq
PLAT245_ALERT_2_C U(iso) H1B     Smaller than U(eq) N1      by ...      0.046 AngSq
PLAT420_ALERT_2_C D-H Without Acceptor        N1     -   H1B    ..     Please Check
```

Alert level G

```
PLAT004_ALERT_5_G Polymeric Structure Found with Maximum Dimension          3 Info
PLAT005_ALERT_5_G No _iucr_refine_instructions_details  in the CIF     Please Do !
PLAT007_ALERT_5_G Number of Unrefined Donor-H Atoms ..............          2 Report
PLAT045_ALERT_1_G Calculated and Reported Z Differ by ............       0.50 Ratio
PLAT072_ALERT_2_G SHELXL First  Parameter in WGHT Unusually Large.       0.11 Report
PLAT093_ALERT_1_G No su's on H-positions, refinement reported as .      mixed Check
```

```
PLAT199_ALERT_1_G Reported _cell_measurement_temperature ..... (K)        293 Check
PLAT200_ALERT_1_G Reported   _diffrn_ambient_temperature ..... (K)        293 Check
PLAT300_ALERT_4_G Atom Site Occupancy of *O1W    is Constrained at      0.500 Check
PLAT302_ALERT_4_G Anion/Solvent Disorder ............ Percentage =          9 Note
PLAT311_ALERT_2_G Isolated Disordered Oxygen Atom (No H's ?) .....        O1W Check
PLAT344_ALERT_2_G Unusual sp?     Angle Range in Solvent/Ion for .     C1     Check
PLAT344_ALERT_2_G Unusual sp?     Angle Range in Solvent/Ion for .     C2     Check
PLAT432_ALERT_2_G Short Inter X...Y Contact  O1W    ..  C1      ..       2.25 Ang.
PLAT432_ALERT_2_G Short Inter X...Y Contact  O1W    ..  C1      ..       2.55 Ang.
PLAT432_ALERT_2_G Short Inter X...Y Contact  N1     ..  C2      ..       2.17 Ang.
PLAT432_ALERT_2_G Short Inter X...Y Contact  C1     ..  C2      ..       1.91 Ang.
PLAT432_ALERT_2_G Short Inter X...Y Contact  C2     ..  C3      ..       3.03 Ang.
PLAT764_ALERT_4_G Overcomplete CIF Bond List Detected (Rep/Expd) .       1.34 Ratio
PLAT773_ALERT_2_G Check long C-C Bond in CIF: C1     --  C2      .       1.92 Ang.
PLAT793_ALERT_4_G The Model has Chirality at P1      (Centro SPGR)          S Verify
PLAT793_ALERT_4_G The Model has Chirality at P2      (Centro SPGR)          R Verify
PLAT793_ALERT_4_G The Model has Chirality at P3      (Centro SPGR)          R Verify
PLAT794_ALERT_5_G Tentative Bond Valency for Al1     (III)   .....       2.98 Note
PLAT794_ALERT_5_G Tentative Bond Valency for Al2     (III)   .....       3.09 Note
PLAT899_ALERT_4_G SHELXL97   is Deprecated and Succeeded by SHELXL       2014 Note
```

---

13 **ALERT level A** = Most likely a serious problem - resolve or explain
10 **ALERT level B** = A potentially serious problem, consider carefully
12 **ALERT level C** = Check. Ensure it is not caused by an omission or oversight
26 **ALERT level G** = General information/check it is not something unexpected

12 ALERT type 1 CIF construction/syntax error, inconsistent or missing data
34 ALERT type 2 Indicator that the structure model may be wrong or deficient
2 ALERT type 3 Indicator that the structure quality may be low
8 ALERT type 4 Improvement, methodology, query or suggestion
5 ALERT type 5 Informative message, check

It is advisable to attempt to resolve as many as possible of the alerts in all categories. Often the minor alerts point to easily fixed oversights, errors and omissions in your CIF or refinement strategy, so attention to these fine details can be worthwhile. In order to resolve some of the more serious problems it may be necessary to carry out additional measurements or structure refinements. However, the purpose of your study may justify the reported deviations and the more serious of these should normally be commented upon in the discussion or experimental section of a paper or in the "special_details" fields of the CIF. checkCIF was carefully designed to identify outliers and unusual parameters, but every test has its limitations and alerts that are not important in a particular case may appear. Conversely, the absence of alerts does not guarantee there are no aspects of the results needing attention. It is up to the individual to critically assess their own results and, if necessary, seek expert advice.

Publication of your CIF in IUCr journals

A basic structural check has been run on your CIF. These basic checks will be run on all CIFs submitted for publication in IUCr journals (*Acta Crystallographica*, *Journal of Applied Crystallography*, *Journal of Synchrotron Radiation*); however, if you intend to submit to *Acta Crystallographica Section C* or *E*, you should make sure that full publication checks are run on the final version of your CIF prior to submission.

Publication of your CIF in other journals

Please refer to the *Notes for Authors* of the relevant journal for any special instructions relating to CIF submission.

PLATON version of 21/06/2015; check.def file version of 21/06/2015

Datablock yp-1 - ellipsoid plot

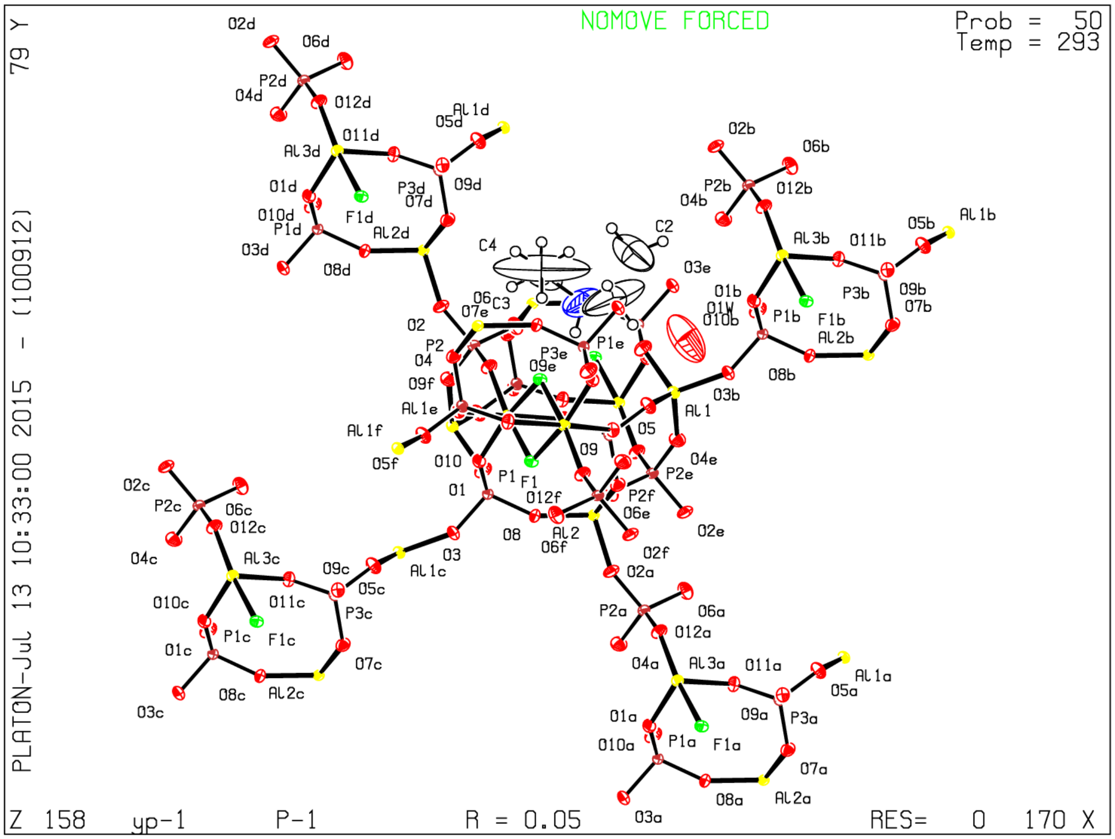

checkCIF/PLATON report

You have not supplied any structure factors. As a result the full set of tests cannot be run.

THIS REPORT IS FOR GUIDANCE ONLY. IF USED AS PART OF A REVIEW PROCEDURE FOR PUBLICATION, IT SHOULD NOT REPLACE THE EXPERTISE OF AN EXPERIENCED CRYSTALLOGRAPHIC REFEREE.

No syntax errors found. CIF dictionary Interpreting this report

Datablock: ywfp-1

```
Bond precision:  C-C = 0.0250 A         Wavelength=0.71073

Cell:          a=9.1231(14)      b=9.2411(14)     c=9.3426(15)
               alpha=86.769(4)   beta=79.946(4)   gamma=87.846(4)
Temperature:   293 K

                Calculated                 Reported
Volume          774.0(2)                   774.0(2)
Space group     P -1                       Triclinic
Hall group      -P 1                       ?
Moiety formula  Al3 F O12 P3, C3 H10 N, O  ?
Sum formula     C3 H10 Al3 F N O13 P3      C3 H12 Al3 F N O13 P3
Mr              460.97                     462.99
Dx,g cm-3       1.978                      1.987
Z               2                          2
Mu (mm-1)       0.633                      0.634
F000            464.0                      468.0
F000'           465.27
h,k,lmax        10,10,10                   10,10,10
Nref            2215                       2207
Tmin,Tmax       0.951,0.951                0.228,0.326
Tmin'           0.951

Correction method= # Reported T Limits: Tmin=0.228 Tmax=0.326
AbsCorr = EMPIRICAL

Data completeness= 0.996          Theta(max)= 23.230

R(reflections)= 0.0560( 1455)     wR2(reflections)= 0.1453( 2207)

S = 0.960                 Npar= 217
```

The following ALERTS were generated. Each ALERT has the format
test-name_ALERT_alert-type_alert-level.
Click on the hyperlinks for more details of the test.

Alert level A

```
EXPT005_ALERT_1_A  _exptl_crystal_description is missing
            Crystal habit description.
            The following tests will not be performed.
            CRYSR_01
DIFF003_ALERT_1_A  _diffrn_measurement_device_type is missing
            Diffractometer make and type. Replaces _diffrn_measurement_type.
SYMMG01_ALERT_1_A          Unrecognised _symmetry_space_group_name_H-M
            International Tables space group number is not in the CIF
           From the CIF: _symmetry_space_group_name_H-M      triclinic
           Int. Tables space group number for triclinic is   0
SYMMG02_ALERT_1_A        Supplied _symmetry_space_group_name_H-M not recognised
           From the CIF: _symmetry_equiv_pos_as_xyz
                                            x, y, z
                                            -x, -y, -z
           These symops generate the Hall space group symbol   -p_1
            which is equivalent to the H-M space group symbol  p_-1
PLAT129_ALERT_4_A Unusual Space group Specified .................    TRICLIN Check
PLAT245_ALERT_2_A U(iso) H1A     Smaller than U(eq) C1      by ...      0.148 AngSq
PLAT245_ALERT_2_A U(iso) H1B     Smaller than U(eq) C1      by ...      0.148 AngSq
PLAT245_ALERT_2_A U(iso) H1C     Smaller than U(eq) C1      by ...      0.148 AngSq
```

Alert level B

```
SYMMS01_ALERT_1_B  The cell setting should be one of the following
            *  triclinic
            *  monoclinic
            *  orthorhombic
            *  tetragonal
            *  rhombohedral
            *  trigonal
            *  hexagonal
            *  cubic
            Cell setting given = P-1
THETM01_ALERT_3_B  The value of sine(theta_max)/wavelength is less than 0.575
            Calculated sin(theta_max)/wavelength =    0.5550
PLAT245_ALERT_2_B U(iso) H3A     Smaller than U(eq) C3      by ...      0.071 AngSq
PLAT245_ALERT_2_B U(iso) H3B     Smaller than U(eq) C3      by ...      0.071 AngSq
PLAT245_ALERT_2_B U(iso) H3C     Smaller than U(eq) C3      by ...      0.071 AngSq
PLAT306_ALERT_2_B Isolated Oxygen Atom (H-atoms Missing ?) .......        Ow1 Check
PLAT340_ALERT_3_B Low Bond Precision on  C-C Bonds ...............     0.0250 Ang.
PLAT430_ALERT_2_B Short Inter D...A Contact  Ow1    ..  Ow1     ..       2.71 Ang.
PLAT430_ALERT_2_B Short Inter D...A Contact  Ow1    ..  O12     ..       2.77 Ang.
```

Alert level C

```
ABSTY02_ALERT_1_C  An _exptl_absorpt_correction_type has been given without
            a literature citation. This should be contained in the
            _exptl_absorpt_process_details field.
            Absorption correction given as empirical
PLAT041_ALERT_1_C Calc. and Reported SumFormula    Strings  Differ     Please Check
PLAT043_ALERT_1_C Calculated and Reported Mol. Weight Differ by ..       2.02 Check
PLAT068_ALERT_1_C Reported F000 Differs from Calcd (or Missing)...     Please Check
PLAT120_ALERT_1_C Reported SPGR Triclin Inconsistent with Explicit      P-1     Chec
PLAT244_ALERT_4_C Low   'Solvent' Ueq as Compared to Neighbors of         C2 Check
PLAT245_ALERT_2_C U(iso) H2      Smaller than U(eq) C2      by ...      0.032 AngSq
```

Alert level G

```
FORMU01_ALERT_2_G  There is a discrepancy between the atom counts in the
            _chemical_formula_sum and the formula from the _atom_site* data.
```

```
            Atom count from _chemical_formula_sum:C3 H12 Al3 F1 N1 O13 P3
            Atom count from the _atom_site data:  C3 H10 Al3 F1 N1 O13 P3
CELLZ01_ALERT_1_G Difference between formula and atom_site contents detected.
CELLZ01_ALERT_1_G WARNING: H atoms missing from atom site list. Is this intentional?
           From the CIF: _cell_formula_units_Z    2
           From the CIF: _chemical_formula_sum  C3 H12 Al3 F N O13 P3
           TEST: Compare cell contents of formula and atom_site data

           atom    Z*formula  cif sites diff
           C          6.00      6.00    0.00
           H         24.00     20.00    4.00
           Al         6.00      6.00    0.00
           F          2.00      2.00    0.00
           N          2.00      2.00    0.00
           O         26.00     26.00    0.00
           P          6.00      6.00    0.00
PLAT004_ALERT_5_G Polymeric Structure Found with Maximum Dimension          3 Info
PLAT005_ALERT_5_G No _iucr_refine_instructions_details  in the CIF     Please Do !
PLAT007_ALERT_5_G Number of Unrefined Donor-H Atoms ..............          3 Report
PLAT093_ALERT_1_G No su’s on H-positions, refinement reported as .      mixed Check
PLAT104_ALERT_1_G The Reported Crystal System is Inconsistent with       P-1 Check
PLAT154_ALERT_1_G The su’s on the Cell Angles are Equal ..........    0.00400 Degree
PLAT199_ALERT_1_G Reported _cell_measurement_temperature ..... (K)        293 Check
PLAT200_ALERT_1_G Reported   _diffrn_ambient_temperature ..... (K)        293 Check
PLAT720_ALERT_4_G Number of Unusual/Non-Standard Labels ..........          1 Note
PLAT764_ALERT_4_G Overcomplete CIF Bond List Detected (Rep/Expd) .       1.28 Ratio
PLAT793_ALERT_4_G The Model has Chirality at P1      (Centro SPGR)          R Verify
PLAT793_ALERT_4_G The Model has Chirality at P2      (Centro SPGR)          R Verify
PLAT793_ALERT_4_G The Model has Chirality at P3      (Centro SPGR)          S Verify
PLAT794_ALERT_5_G Tentative Bond Valency for Al1     (III)   .....       3.12 Note
PLAT794_ALERT_5_G Tentative Bond Valency for Al3     (III)   .....       2.95 Note
PLAT899_ALERT_4_G SHELXL97   is Deprecated and Succeeded by SHELXL       2014 Note
```

---

   8 **ALERT level A** = Most likely a serious problem - resolve or explain
   9 **ALERT level B** = A potentially serious problem, consider carefully
   7 **ALERT level C** = Check. Ensure it is not caused by an omission or oversight
  19 **ALERT level G** = General information/check it is not something unexpected

  17 ALERT type 1 CIF construction/syntax error, inconsistent or missing data
  11 ALERT type 2 Indicator that the structure model may be wrong or deficient
   2 ALERT type 3 Indicator that the structure quality may be low
   8 ALERT type 4 Improvement, methodology, query or suggestion
   5 ALERT type 5 Informative message, check

It is advisable to attempt to resolve as many as possible of the alerts in all categories. Often the minor alerts point to easily fixed oversights, errors and omissions in your CIF or refinement strategy, so attention to these fine details can be worthwhile. In order to resolve some of the more serious problems it may be necessary to carry out additional measurements or structure refinements. However, the purpose of your study may justify the reported deviations and the more serious of these should normally be commented upon in the discussion or experimental section of a paper or in the "special_details" fields of the CIF. checkCIF was carefully designed to identify outliers and unusual parameters, but every test has its limitations and alerts that are not important in a particular case may appear. Conversely, the absence of alerts does not guarantee there are no aspects of the results needing attention. It is up to the individual to critically assess their own results and, if necessary, seek expert advice.

Publication of your CIF in IUCr journals

A basic structural check has been run on your CIF. These basic checks will be run on all CIFs submitted for publication in IUCr journals (*Acta Crystallographica*, *Journal of Applied Crystallography*, *Journal of Synchrotron Radiation*); however, if you intend to submit to *Acta Crystallographica Section C* or *E*, you should make sure that full publication checks are run on the final version of your CIF prior to submission.

Publication of your CIF in other journals

Please refer to the *Notes for Authors* of the relevant journal for any special instructions relating to CIF submission.

PLATON version of 21/06/2015; check.def file version of 21/06/2015

Datablock ywfp-1 - ellipsoid plot

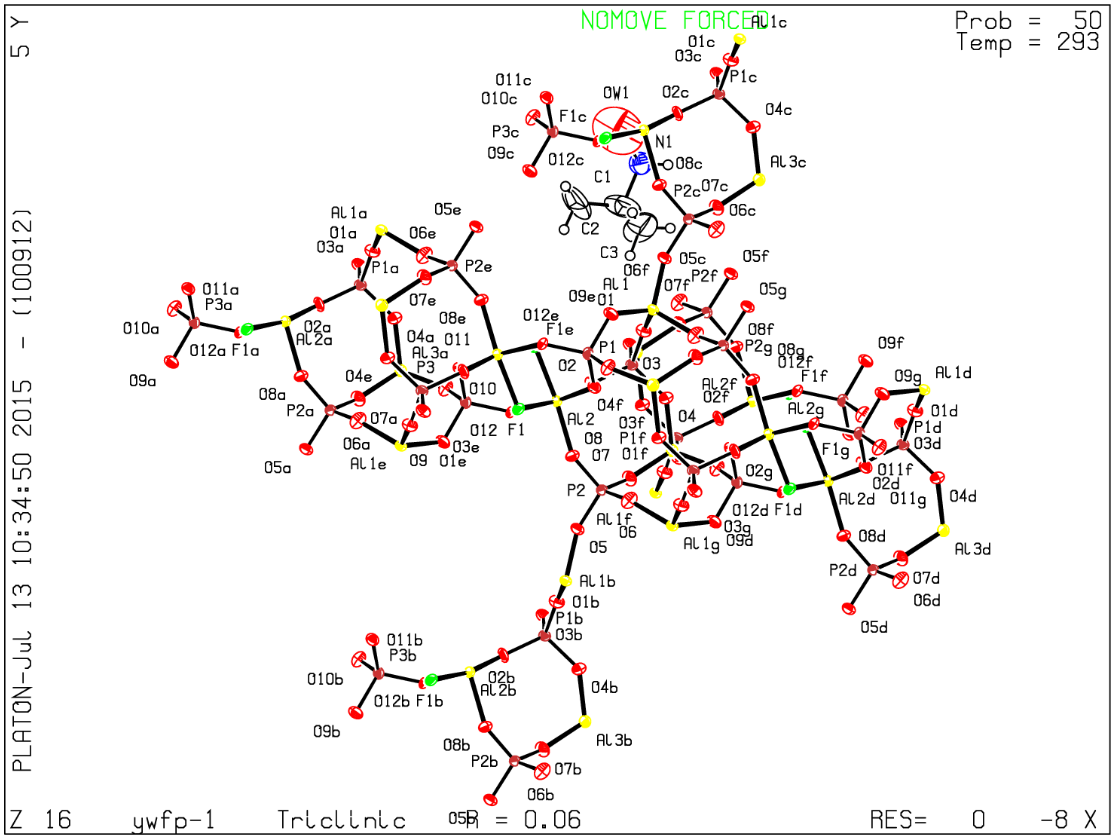

checkCIF/PLATON report

You have not supplied any structure factors. As a result the full set of tests cannot be run.

THIS REPORT IS FOR GUIDANCE ONLY. IF USED AS PART OF A REVIEW PROCEDURE FOR PUBLICATION, IT SHOULD NOT REPLACE THE EXPERTISE OF AN EXPERIENCED CRYSTALLOGRAPHIC REFEREE.

No syntax errors found. CIF dictionary Interpreting this report

Datablock: 3.1.1.2.004_2

```
Bond precision:  = 0.0000 A             Wavelength=0.71073

Cell:            a=9.33300        b=9.18300        c=9.16200
                 alpha=88.4500    beta=102.5700    gamma=93.7600
Temperature:     0 K

                Calculated                 Reported
Volume          764.692                    0
Space group     P -1                       P-1
Hall group      -P 1                       ?
Moiety formula  Al3 F O12 P3, C4 H10 N O   ?
Sum formula     C4 H10 Al3 F N O13 P3      ?
Mr              472.98                     0.00
Dx,g cm-3       2.054                      0.000
Z               2                          0
Mu (mm-1)       0.644                      0.000
F000            476.0                      0.0
F000'           477.27
h,k,lmax
Nref
Tmin,Tmax
Tmin'

Correction method= Not given

Data completeness=                Theta(max)=

R(reflections)=                   wR2(reflections)=

S =                          Npar=
```

The following ALERTS were generated. Each ALERT has the format
test-name_ALERT_alert-type_alert-level.
Click on the hyperlinks for more details of the test.

Alert level A

```
EXPT005_ALERT_1_A  _exptl_crystal_description is missing
            Crystal habit description.
            The following tests will not be performed.
            CRYSR_01
DIFF003_ALERT_1_A  _diffrn_measurement_device_type is missing
            Diffractometer make and type. Replaces _diffrn_measurement_type.
ATOM007_ALERT_1_A  _atom_site_aniso_label is missing
            Unique label identifying the atom site.
GEOM006_ALERT_1_A  _geom_angle_atom_site_label_2 is missing
            Label identifying the atom site 2.
GEOM007_ALERT_1_A  _geom_angle_atom_site_label_3 is missing
            Label identifying the atom site 3.
PLAT029_ALERT_3_A _diffrn_measured_fraction_theta_full Low .......      0.000 Note
PLAT043_ALERT_1_A Calculated and Reported Mol. Weight Differ by ..     472.98 Check
PLAT091_ALERT_1_A No Wavelength found in CIF - 0.71073 Ang Assumed     Please Check
PLAT197_ALERT_1_A Missing            _cell_measurement_temperature     Please Suppl
PLAT198_ALERT_1_A Missing              _diffrn_ambient_temperature     Please Suppl
```

Alert level C

```
PLAT141_ALERT_4_C su on a - Axis Small or Missing ...............    0.00000 Ang.
PLAT142_ALERT_4_C su on b - Axis Small or Missing ...............    0.00000 Ang.
PLAT143_ALERT_4_C su on c - Axis Small or Missing ...............    0.00000 Ang.
PLAT144_ALERT_4_C su on alpha    Small or Missing ...............     0.0000 Degree
PLAT145_ALERT_4_C su on beta     Small or Missing ...............     0.0000 Degree
PLAT146_ALERT_4_C su on gamma    Small or Missing ...............     0.0000 Degree
PLAT155_ALERT_4_C The Triclinic Unitcell is NOT Reduced ..........     Please Do !
PLAT161_ALERT_4_C Missing or Zero su (esd) on x-coordinate for ...        AL1
PLAT161_ALERT_4_C Missing or Zero su (esd) on x-coordinate for ...        AL2
PLAT161_ALERT_4_C Missing or Zero su (esd) on x-coordinate for ...        AL3
PLAT161_ALERT_4_C Missing or Zero su (esd) on x-coordinate for ...         P1
PLAT161_ALERT_4_C Missing or Zero su (esd) on x-coordinate for ...         P2
PLAT161_ALERT_4_C Missing or Zero su (esd) on x-coordinate for ...         P3
PLAT161_ALERT_4_C Missing or Zero su (esd) on x-coordinate for ...         O1
PLAT161_ALERT_4_C Missing or Zero su (esd) on x-coordinate for ...         O2
PLAT161_ALERT_4_C Missing or Zero su (esd) on x-coordinate for ...         O3
PLAT161_ALERT_4_C Missing or Zero su (esd) on x-coordinate for ...         O4
PLAT161_ALERT_4_C Missing or Zero su (esd) on x-coordinate for ...         O5
PLAT161_ALERT_4_C Missing or Zero su (esd) on x-coordinate for ...         O6
PLAT161_ALERT_4_C Missing or Zero su (esd) on x-coordinate for ...         O7
PLAT161_ALERT_4_C Missing or Zero su (esd) on x-coordinate for ...         O8
PLAT161_ALERT_4_C Missing or Zero su (esd) on x-coordinate for ...         O9
PLAT161_ALERT_4_C Missing or Zero su (esd) on x-coordinate for ...        O10
PLAT161_ALERT_4_C Missing or Zero su (esd) on x-coordinate for ...        O11
PLAT161_ALERT_4_C Missing or Zero su (esd) on x-coordinate for ...        O12
PLAT161_ALERT_4_C Missing or Zero su (esd) on x-coordinate for ...        O13
PLAT161_ALERT_4_C Missing or Zero su (esd) on x-coordinate for ...         C1
PLAT161_ALERT_4_C Missing or Zero su (esd) on x-coordinate for ...         C2
PLAT161_ALERT_4_C Missing or Zero su (esd) on x-coordinate for ...         C3
PLAT161_ALERT_4_C Missing or Zero su (esd) on x-coordinate for ...         C4
PLAT161_ALERT_4_C Missing or Zero su (esd) on x-coordinate for ...         N1
PLAT161_ALERT_4_C Missing or Zero su (esd) on x-coordinate for ...         F1
PLAT162_ALERT_4_C Missing or Zero su (esd) on y-coordinate for ...        AL1
PLAT162_ALERT_4_C Missing or Zero su (esd) on y-coordinate for ...        AL2
PLAT162_ALERT_4_C Missing or Zero su (esd) on y-coordinate for ...        AL3
PLAT162_ALERT_4_C Missing or Zero su (esd) on y-coordinate for ...         P1
PLAT162_ALERT_4_C Missing or Zero su (esd) on y-coordinate for ...         P2
PLAT162_ALERT_4_C Missing or Zero su (esd) on y-coordinate for ...         P3
PLAT162_ALERT_4_C Missing or Zero su (esd) on y-coordinate for ...         O1
PLAT162_ALERT_4_C Missing or Zero su (esd) on y-coordinate for ...         O2
```

```
PLAT162_ALERT_4_C Missing or Zero su (esd) on y-coordinate for ...         O3
PLAT162_ALERT_4_C Missing or Zero su (esd) on y-coordinate for ...         O4
PLAT162_ALERT_4_C Missing or Zero su (esd) on y-coordinate for ...         O5
PLAT162_ALERT_4_C Missing or Zero su (esd) on y-coordinate for ...         O6
PLAT162_ALERT_4_C Missing or Zero su (esd) on y-coordinate for ...         O7
PLAT162_ALERT_4_C Missing or Zero su (esd) on y-coordinate for ...         O8
PLAT162_ALERT_4_C Missing or Zero su (esd) on y-coordinate for ...         O9
PLAT162_ALERT_4_C Missing or Zero su (esd) on y-coordinate for ...        O10
PLAT162_ALERT_4_C Missing or Zero su (esd) on y-coordinate for ...        O11
PLAT162_ALERT_4_C Missing or Zero su (esd) on y-coordinate for ...        O12
PLAT162_ALERT_4_C Missing or Zero su (esd) on y-coordinate for ...        O13
PLAT162_ALERT_4_C Missing or Zero su (esd) on y-coordinate for ...         C1
PLAT162_ALERT_4_C Missing or Zero su (esd) on y-coordinate for ...         C2
PLAT162_ALERT_4_C Missing or Zero su (esd) on y-coordinate for ...         C3
PLAT162_ALERT_4_C Missing or Zero su (esd) on y-coordinate for ...         C4
PLAT162_ALERT_4_C Missing or Zero su (esd) on y-coordinate for ...         N1
PLAT162_ALERT_4_C Missing or Zero su (esd) on y-coordinate for ...         F1
PLAT163_ALERT_4_C Missing or Zero su (esd) on z-coordinate for ...        AL1
PLAT163_ALERT_4_C Missing or Zero su (esd) on z-coordinate for ...        AL2
PLAT163_ALERT_4_C Missing or Zero su (esd) on z-coordinate for ...        AL3
PLAT163_ALERT_4_C Missing or Zero su (esd) on z-coordinate for ...         P1
PLAT163_ALERT_4_C Missing or Zero su (esd) on z-coordinate for ...         P2
PLAT163_ALERT_4_C Missing or Zero su (esd) on z-coordinate for ...         P3
PLAT163_ALERT_4_C Missing or Zero su (esd) on z-coordinate for ...         O1
PLAT163_ALERT_4_C Missing or Zero su (esd) on z-coordinate for ...         O2
PLAT163_ALERT_4_C Missing or Zero su (esd) on z-coordinate for ...         O3
PLAT163_ALERT_4_C Missing or Zero su (esd) on z-coordinate for ...         O4
PLAT163_ALERT_4_C Missing or Zero su (esd) on z-coordinate for ...         O5
PLAT163_ALERT_4_C Missing or Zero su (esd) on z-coordinate for ...         O6
PLAT163_ALERT_4_C Missing or Zero su (esd) on z-coordinate for ...         O7
PLAT163_ALERT_4_C Missing or Zero su (esd) on z-coordinate for ...         O8
PLAT163_ALERT_4_C Missing or Zero su (esd) on z-coordinate for ...         O9
PLAT163_ALERT_4_C Missing or Zero su (esd) on z-coordinate for ...        O10
PLAT163_ALERT_4_C Missing or Zero su (esd) on z-coordinate for ...        O11
PLAT163_ALERT_4_C Missing or Zero su (esd) on z-coordinate for ...        O12
PLAT163_ALERT_4_C Missing or Zero su (esd) on z-coordinate for ...        O13
PLAT163_ALERT_4_C Missing or Zero su (esd) on z-coordinate for ...         C1
PLAT163_ALERT_4_C Missing or Zero su (esd) on z-coordinate for ...         C2
PLAT163_ALERT_4_C Missing or Zero su (esd) on z-coordinate for ...         C3
PLAT163_ALERT_4_C Missing or Zero su (esd) on z-coordinate for ...         C4
PLAT163_ALERT_4_C Missing or Zero su (esd) on z-coordinate for ...         N1
PLAT163_ALERT_4_C Missing or Zero su (esd) on z-coordinate for ...         F1
PLAT202_ALERT_3_C Isotropic non-H Atoms in Anion/Solvent .........          6
```

Alert level G

```
PLAT004_ALERT_5_G Polymeric Structure Found with Maximum Dimension          3 Info
PLAT005_ALERT_5_G No _iucr_refine_instructions_details  in the CIF     Please Do !
PLAT007_ALERT_5_G Number of Unrefined Donor-H Atoms ..............          2 Report
PLAT045_ALERT_1_G Calculated and Reported Z Differ by ............       0.00 Ratio
PLAT194_ALERT_1_G Missing _cell_measurement_reflns_used datum ....     Please Do !
PLAT195_ALERT_1_G Missing _cell_measurement_theta_max   datum ....     Please Do !
PLAT196_ALERT_1_G Missing _cell_measurement_theta_min   datum ....     Please Do !
PLAT199_ALERT_1_G Reported _cell_measurement_temperature ..... (K)    -999999 Check
PLAT344_ALERT_2_G Unusual sp3     Angle Range in Solvent/Ion for .     C1     Check
PLAT793_ALERT_4_G The Model has Chirality at P2      (Centro SPGR)          S Verify
PLAT794_ALERT_5_G Tentative Bond Valency for Al2     (III)   .....       3.22 Note
PLAT794_ALERT_5_G Tentative Bond Valency for Al3     (III)   .....       2.99 Note
PLAT808_ALERT_5_G No Parseable SHELXL Style Weighting Scheme Found     Please Check
PLAT980_ALERT_1_G No Anomalous Scattering Factors Found in CIF ...     Please Check
```

```
10 ALERT level A = Most likely a serious problem - resolve or explain
 0 ALERT level B = A potentially serious problem, consider carefully
83 ALERT level C = Check. Ensure it is not caused by an omission or oversight
14 ALERT level G = General information/check it is not something unexpected

15 ALERT type 1 CIF construction/syntax error, inconsistent or missing data
 1 ALERT type 2 Indicator that the structure model may be wrong or deficient
 2 ALERT type 3 Indicator that the structure quality may be low
83 ALERT type 4 Improvement, methodology, query or suggestion
 6 ALERT type 5 Informative message, check
```

It is advisable to attempt to resolve as many as possible of the alerts in all categories. Often the minor alerts point to easily fixed oversights, errors and omissions in your CIF or refinement strategy, so attention to these fine details can be worthwhile. In order to resolve some of the more serious problems it may be necessary to carry out additional measurements or structure refinements. However, the purpose of your study may justify the reported deviations and the more serious of these should normally be commented upon in the discussion or experimental section of a paper or in the "special_details" fields of the CIF. checkCIF was carefully designed to identify outliers and unusual parameters, but every test has its limitations and alerts that are not important in a particular case may appear. Conversely, the absence of alerts does not guarantee there are no aspects of the results needing attention. It is up to the individual to critically assess their own results and, if necessary, seek expert advice.

Publication of your CIF in IUCr journals

A basic structural check has been run on your CIF. These basic checks will be run on all CIFs submitted for publication in IUCr journals (*Acta Crystallographica*, *Journal of Applied Crystallography*, *Journal of Synchrotron Radiation*); however, if you intend to submit to *Acta Crystallographica Section C* or *E*, you should make sure that full publication checks are run on the final version of your CIF prior to submission.

Publication of your CIF in other journals

Please refer to the *Notes for Authors* of the relevant journal for any special instructions relating to CIF submission.

PLATON version of 21/06/2015; check.def file version of 21/06/2015

Datablock 3.1.1.2.004_2 - ellipsoid plot

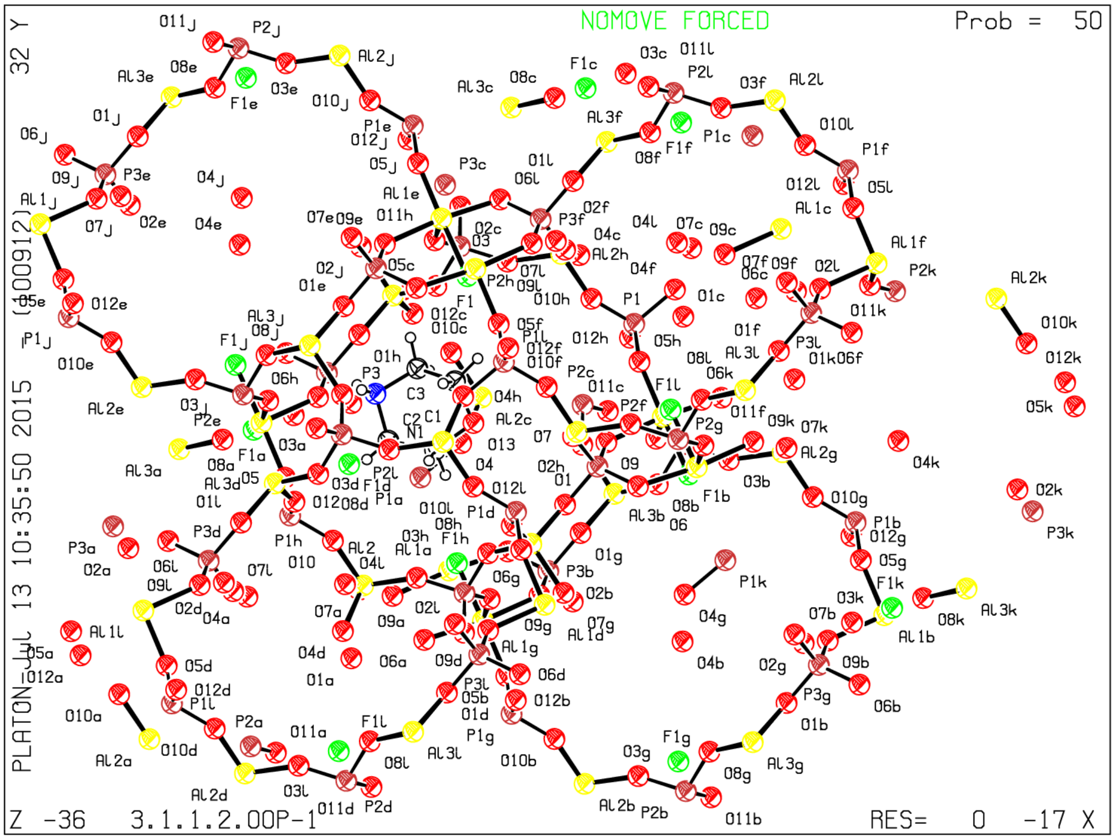

checkCIF/PLATON report

You have not supplied any structure factors. As a result the full set of tests cannot be run.

THIS REPORT IS FOR GUIDANCE ONLY. IF USED AS PART OF A REVIEW PROCEDURE FOR PUBLICATION, IT SHOULD NOT REPLACE THE EXPERTISE OF AN EXPERIENCED CRYSTALLOGRAPHIC REFEREE.

No syntax errors found. CIF dictionary Interpreting this report

Datablock: yp-1

Bond precision: C-C = 0.0180 A Wavelength=0.71073

Cell:	a=9.1800(8)	b=9.1957(8)	c=9.3606(8)
	alpha=86.532(2)	beta=78.192(2)	gamma=87.739(2)
Temperature:	293 K		

	Calculated	Reported
Volume	771.76(12)	771.76(12)
Space group	P -1	?
Hall group	-P 1	?
Moiety formula	Al3 F O12 P3, C5 H12 N, O0.25	?
Sum formula	C5 H12 Al3 F N O12.25 P3	C5 H12.50 Al3 F N O12.25 P3
Mr	475.01	475.51
Dx,g cm-3	2.044	2.046
Z	2	2
Mu (mm-1)	0.635	0.635
F000	480.0	481.0
F000’	481.26	
h,k,lmax	10,10,10	10,10,10
Nref	2214	2206
Tmin,Tmax		
Tmin’		

Correction method= Not given

Data completeness= 0.996 Theta(max)= 23.250

R(reflections)= 0.0493(1705) wR2(reflections)= 0.1232(2206)

S = 1.037 Npar= 235

The following ALERTS were generated. Each ALERT has the format
test-name_ALERT_alert-type_alert-level.
Click on the hyperlinks for more details of the test.

Alert level A

```
SYMM001_ALERT_1_A  _symmetry_cell_setting is missing
            The cell setting should be one of the following
            *  triclinic
            *  monoclinic
            *  orthorhombic
            *  tetragonal
            *  rhombohedral
            *  trigonal
            *  hexagonal
            *  cubic
            The following tests will not be performed.
            SYMMS_01,SYMMS_02
EXPT005_ALERT_1_A  _exptl_crystal_description is missing
            Crystal habit description.
            The following tests will not be performed.
            CRYSR_01
DIFF003_ALERT_1_A  _diffrn_measurement_device_type is missing
            Diffractometer make and type. Replaces _diffrn_measurement_type.
PLAT122_ALERT_1_A No _symmetry_space_group_name_H-M Given ........     Please Do !
PLAT430_ALERT_2_A Short Inter D...A Contact  O1W    ..  O1W     ..       2.04 Ang.
```

Alert level B

```
THETM01_ALERT_3_B  The value of sine(theta_max)/wavelength is less than 0.575
            Calculated sin(theta_max)/wavelength =    0.5554
PLAT245_ALERT_2_B U(iso) H2A     Smaller than U(eq) C2      by ...      0.067 AngSq
PLAT245_ALERT_2_B U(iso) H2B     Smaller than U(eq) C2      by ...      0.067 AngSq
PLAT340_ALERT_3_B Low Bond Precision on  C-C Bonds ..............     0.0180 Ang.
```

Alert level C

```
PLAT041_ALERT_1_C Calc. and Reported SumFormula    Strings  Differ     Please Check
PLAT043_ALERT_1_C Calculated and Reported Mol. Weight Differ by ..       0.50 Check
PLAT052_ALERT_1_C Info on Absorption Correction Method Not Given .     Please Do !
PLAT053_ALERT_1_C Minimum Crystal Dimension Missing (or Error) ...     Please Check
PLAT054_ALERT_1_C Medium  Crystal Dimension Missing (or Error) ...     Please Check
PLAT055_ALERT_1_C Maximum Crystal Dimension Missing (or Error) ...     Please Check
PLAT077_ALERT_4_C Unitcell contains non-integer number of atoms ..     Please Check
PLAT088_ALERT_3_C Poor Data / Parameter Ratio ....................       9.42 Note
PLAT214_ALERT_2_C Atom C1      (Anion/Solvent) ADP max/min Ratio          4.2 prolat
PLAT243_ALERT_4_C High  'Solvent' Ueq as Compared to Neighbors of          C2 Check
PLAT243_ALERT_4_C High  'Solvent' Ueq as Compared to Neighbors of          C5 Check
PLAT244_ALERT_4_C Low   'Solvent' Ueq as Compared to Neighbors of          N1 Check
PLAT244_ALERT_4_C Low   'Solvent' Ueq as Compared to Neighbors of          C3 Check
PLAT245_ALERT_2_C U(iso) H1A     Smaller than U(eq) N1      by ...      0.028 AngSq
PLAT245_ALERT_2_C U(iso) H1B     Smaller than U(eq) N1      by ...      0.028 AngSq
PLAT245_ALERT_2_C U(iso) H1C     Smaller than U(eq) C1      by ...      0.045 AngSq
PLAT245_ALERT_2_C U(iso) H1D     Smaller than U(eq) C1      by ...      0.045 AngSq
PLAT245_ALERT_2_C U(iso) H4A     Smaller than U(eq) C4      by ...      0.013 AngSq
PLAT245_ALERT_2_C U(iso) H4B     Smaller than U(eq) C4      by ...      0.013 AngSq
PLAT245_ALERT_2_C U(iso) H5A     Smaller than U(eq) C5      by ...      0.046 AngSq
PLAT245_ALERT_2_C U(iso) H5B     Smaller than U(eq) C5      by ...      0.046 AngSq
PLAT360_ALERT_2_C Short  C(sp3)-C(sp3) Bond   C1     -   C2      ..       1.40 Ang.
PLAT360_ALERT_2_C Short  C(sp3)-C(sp3) Bond   C2     -   C3      ..       1.34 Ang.
PLAT360_ALERT_2_C Short  C(sp3)-C(sp3) Bond   C3     -   C4      ..       1.41 Ang.
```

```
PLAT360_ALERT_2_C Short  C(sp3)-C(sp3) Bond   C4     -   C5     ..       1.42 Ang.
PLAT420_ALERT_2_C D-H Without Acceptor        N1     -   H1A    ..     Please Check
PLAT420_ALERT_2_C D-H Without Acceptor        N1     -   H1B    ..     Please Check
```

Alert level G

```
FORMU01_ALERT_2_G  There is a discrepancy between the atom counts in the
            _chemical_formula_sum and the formula from the _atom_site* data.
            Atom count from _chemical_formula_sum:C5 H12.5 Al3 F1 N1 O12.25 P3
            Atom count from the _atom_site data:  C5 H12 Al3 F1 N1 O12.25 P3
PLAT004_ALERT_5_G Polymeric Structure Found with Maximum Dimension         3 Info
PLAT005_ALERT_5_G No _iucr_refine_instructions_details  in the CIF     Please Do !
PLAT007_ALERT_5_G Number of Unrefined Donor-H Atoms ..............         2 Report
PLAT068_ALERT_1_G Reported F000 Differs from Calcd (or Missing)...     Please Check
PLAT093_ALERT_1_G No su's on H-positions, refinement reported as .      mixed Check
PLAT104_ALERT_1_G The Reported Crystal System is Inconsistent with       P-1 Check
PLAT154_ALERT_1_G The su's on the Cell Angles are Equal ..........    0.00200 Degree
PLAT199_ALERT_1_G Reported _cell_measurement_temperature ..... (K)       293 Check
PLAT200_ALERT_1_G Reported   _diffrn_ambient_temperature ..... (K)       293 Check
PLAT300_ALERT_4_G Atom Site Occupancy of <O1W    is Constrained at      0.250 Check
PLAT302_ALERT_4_G Anion/Solvent Disorder ............ Percentage =         4 Note
PLAT311_ALERT_2_G Isolated Disordered Oxygen Atom (No H's ?) .....       O1W Check
PLAT432_ALERT_2_G Short Inter X...Y Contact  O1W    ..  C1      ..       2.89 Ang.
PLAT764_ALERT_4_G Overcomplete CIF Bond List Detected (Rep/Expd) .       1.22 Ratio
PLAT793_ALERT_4_G The Model has Chirality at P1      (Centro SPGR)         R Verify
PLAT793_ALERT_4_G The Model has Chirality at P2      (Centro SPGR)         S Verify
PLAT793_ALERT_4_G The Model has Chirality at P3      (Centro SPGR)         R Verify
PLAT794_ALERT_5_G Tentative Bond Valency for Al2     (III)   .....       2.95 Note
PLAT899_ALERT_4_G SHELXL97   is Deprecated and Succeeded by SHELXL       2014 Note
```

---

5 **ALERT level A** = Most likely a serious problem - resolve or explain
4 **ALERT level B** = A potentially serious problem, consider carefully
27 **ALERT level C** = Check. Ensure it is not caused by an omission or oversight
20 **ALERT level G** = General information/check it is not something unexpected

16 ALERT type 1 CIF construction/syntax error, inconsistent or missing data
21 ALERT type 2 Indicator that the structure model may be wrong or deficient
3 ALERT type 3 Indicator that the structure quality may be low
12 ALERT type 4 Improvement, methodology, query or suggestion
4 ALERT type 5 Informative message, check

It is advisable to attempt to resolve as many as possible of the alerts in all categories. Often the minor alerts point to easily fixed oversights, errors and omissions in your CIF or refinement strategy, so attention to these fine details can be worthwhile. In order to resolve some of the more serious problems it may be necessary to carry out additional measurements or structure refinements. However, the purpose of your study may justify the reported deviations and the more serious of these should normally be commented upon in the discussion or experimental section of a paper or in the "special_details" fields of the CIF. checkCIF was carefully designed to identify outliers and unusual parameters, but every test has its limitations and alerts that are not important in a particular case may appear. Conversely, the absence of alerts does not guarantee there are no aspects of the results needing attention. It is up to the individual to critically assess their own results and, if necessary, seek expert advice.

Publication of your CIF in IUCr journals

A basic structural check has been run on your CIF. These basic checks will be run on all CIFs submitted for publication in IUCr journals (*Acta Crystallographica*, *Journal of Applied Crystallography*, *Journal of Synchrotron Radiation*); however, if you intend to submit to *Acta Crystallographica Section C* or *E*, you should make sure that full publication checks are run on the final version of your CIF prior to submission.

Publication of your CIF in other journals

Please refer to the *Notes for Authors* of the relevant journal for any special instructions relating to CIF submission.

PLATON version of 21/06/2015; check.def file version of 21/06/2015

Datablock yp-1 - ellipsoid plot

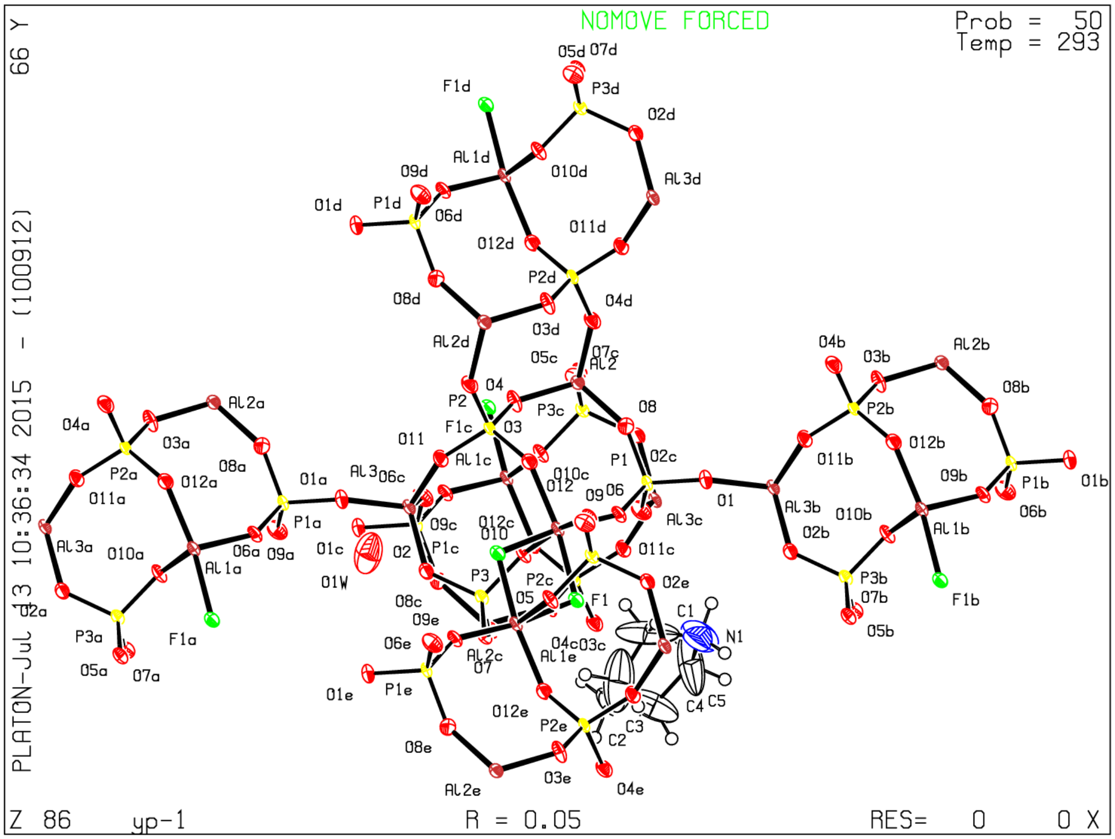

checkCIF/PLATON report

You have not supplied any structure factors. As a result the full set of tests cannot be run.

THIS REPORT IS FOR GUIDANCE ONLY. IF USED AS PART OF A REVIEW PROCEDURE FOR PUBLICATION, IT SHOULD NOT REPLACE THE EXPERTISE OF AN EXPERIENCED CRYSTALLOGRAPHIC REFEREE.

No syntax errors found.   CIF dictionary   Interpreting this report

Datablock: NAYPIM

```
Bond precision:  C-C = 0.0072 A          Wavelength=0.71073

Cell:           a=9.118(1)        b=9.161(1)       c=9.335(1)
                alpha=85.98(1)    beta=77.45(1)    gamma=89.01(1)
Temperature:    0 K
```

	Calculated	Reported
Volume	759.25(15)	759
Space group	P -1	P -1
Hall group	-P 1	?
Moiety formula	Al3 F O12 P3, C5 H6 N, 0.5(O2)	(C5 H6 N1 1+)N,N(Al3 F1 O12 P3 1-),0.15N(H2 O1)
Sum formula	C5 H6 Al3 F N O13 P3	C5 H6.3 Al3 F1 N1 O12.15 P3
Mr	480.96	0.00
Dx,g cm-3	2.104	2.046
Z	2	2
Mu (mm-1)	0.651	0.000
F000	480.0	0.0
F000’	481.28	
h,k,lmax		
Nref		
Tmin,Tmax		
Tmin’		

```
Correction method= Not given

Data completeness=                Theta(max)=

R(reflections)= 0.0280( 0)        wR2(reflections)= wR= 0.0280( 0)

S = ******                 Npar= *****
```

The following ALERTS were generated. Each ALERT has the format
test-name_ALERT_alert-type_alert-level.
Click on the hyperlinks for more details of the test.

Alert level A

```
EXPT005_ALERT_1_A  _exptl_crystal_description is missing
            Crystal habit description.
            The following tests will not be performed.
            CRYSR_01
DIFF003_ALERT_1_A  _diffrn_measurement_device_type is missing
            Diffractometer make and type. Replaces _diffrn_measurement_type.
ATOM007_ALERT_1_A  _atom_site_aniso_label is missing
            Unique label identifying the atom site.
GEOM001_ALERT_1_A  _geom_bond_atom_site_label_1 is missing
            Label identifying the atom site 1.
GEOM003_ALERT_1_A  _geom_bond_distance is missing
            Distance between atom sites 1 and 2.
GEOM006_ALERT_1_A  _geom_angle_atom_site_label_2 is missing
            Label identifying the atom site 2.
GEOM007_ALERT_1_A  _geom_angle_atom_site_label_3 is missing
            Label identifying the atom site 3.
PLAT029_ALERT_3_A _diffrn_measured_fraction_theta_full Low .......      0.000 Note
PLAT043_ALERT_1_A Calculated and Reported Mol. Weight Differ by ..     480.96 Check
PLAT046_ALERT_1_A Reported Z, MW and D(calc) are Inconsistent ....      0.000
PLAT091_ALERT_1_A No Wavelength found in CIF - 0.71073 Ang Assumed     Please Check
PLAT197_ALERT_1_A Missing           _cell_measurement_temperature     Please Suppl
PLAT198_ALERT_1_A Missing             _diffrn_ambient_temperature     Please Suppl
PLAT201_ALERT_2_A Isotropic non-H Atoms in Main Residue(s) .......         19 Report
```

Alert level C

```
PLAT041_ALERT_1_C Calc. and Reported SumFormula    Strings  Differ     Please Check
PLAT151_ALERT_1_C No su (esd) Given on Volume ....................     Please Do !
PLAT202_ALERT_3_C Isotropic non-H Atoms in Anion/Solvent .........          7
PLAT340_ALERT_3_C Low Bond Precision on  C-C Bonds ...............     0.0072 Ang.
```

Alert level G

```
FORMU01_ALERT_1_G  There is a discrepancy between the atom counts in the
            _chemical_formula_sum and _chemical_formula_moiety. This is
            usually due to the moiety formula being in the wrong format.
            Atom count from _chemical_formula_sum:   C5 H6.3 Al3 F1 N1 O12.15 P3
            Atom count from _chemical_formula_moiety:H0.3 O0.15
FORMU01_ALERT_2_G  There is a discrepancy between the atom counts in the
            _chemical_formula_sum and the formula from the _atom_site* data.
            Atom count from _chemical_formula_sum:C5 H6.3 Al3 F1 N1 O12.15 P3
            Atom count from the _atom_site data:  C5 H6 Al9 F2 N1 O20 P3
CELLZ01_ALERT_1_G Difference between formula and atom_site contents detected.
CELLZ01_ALERT_1_G ALERT: Large difference may be due to a
            symmetry error - see SYMMG tests
           From the CIF: _cell_formula_units_Z    2
           From the CIF: _chemical_formula_sum  C5 H6.3 Al3 F1 N1 O12.15 P3
           TEST: Compare cell contents of formula and atom_site data
           Note that site occupancies missing: assumed 1.0

           atom    Z*formula  cif sites diff
           C         10.00     10.00    0.00
           H         12.60     12.00    0.60
           Al         6.00     18.00  -12.00
           F          2.00      4.00   -2.00
```

```
           N          2.00      2.00    0.00
           O         24.30     40.00  -15.70
           P          6.00      6.00    0.00
PLAT004_ALERT_5_G Polymeric Structure Found with Maximum Dimension          3 Info
PLAT005_ALERT_5_G No _iucr_refine_instructions_details  in the CIF     Please Do !
PLAT042_ALERT_1_G Calc. and Reported MoietyFormula Strings  Differ     Please Check
PLAT154_ALERT_1_G The su’s on the Cell Angles are Equal ..........    0.01000 Degree
PLAT194_ALERT_1_G Missing _cell_measurement_reflns_used datum ....     Please Do !
PLAT195_ALERT_1_G Missing _cell_measurement_theta_max   datum ....     Please Do !
PLAT196_ALERT_1_G Missing _cell_measurement_theta_min   datum ....     Please Do !
PLAT199_ALERT_1_G Reported _cell_measurement_temperature ..... (K)    -999999 Check
PLAT720_ALERT_4_G Number of Unusual/Non-Standard Labels ..........          5 Note
PLAT793_ALERT_4_G The Model has Chirality at P1      (Centro SPGR)          R Verify
PLAT793_ALERT_4_G The Model has Chirality at P2      (Centro SPGR)          S Verify
PLAT793_ALERT_4_G The Model has Chirality at P3      (Centro SPGR)          R Verify
PLAT794_ALERT_5_G Tentative Bond Valency for Al1     (III)   .....       3.08 Note
PLAT794_ALERT_5_G Tentative Bond Valency for Al3     (III)   .....       2.97 Note
PLAT804_ALERT_5_G Number of ARU-Code Packing Problem(s) in PLATON           1 Info
PLAT808_ALERT_5_G No Parseable SHELXL Style Weighting Scheme Found     Please Check
PLAT981_ALERT_1_G No non-zero f" Anomalous Scattering Values Found     Please Check
PLAT986_ALERT_1_G No non-zero f’ Anomalous Scattering Values Found     Please Check
```

---

14 **ALERT level A** = Most likely a serious problem - resolve or explain
0 **ALERT level B** = A potentially serious problem, consider carefully
4 **ALERT level C** = Check. Ensure it is not caused by an omission or oversight
22 **ALERT level G** = General information/check it is not something unexpected

```
 25 ALERT type 1 CIF construction/syntax error, inconsistent or missing data
  2 ALERT type 2 Indicator that the structure model may be wrong or deficient
  3 ALERT type 3 Indicator that the structure quality may be low
  4 ALERT type 4 Improvement, methodology, query or suggestion
  6 ALERT type 5 Informative message, check
```

It is advisable to attempt to resolve as many as possible of the alerts in all categories. Often the minor alerts point to easily fixed oversights, errors and omissions in your CIF or refinement strategy, so attention to these fine details can be worthwhile. In order to resolve some of the more serious problems it may be necessary to carry out additional measurements or structure refinements. However, the purpose of your study may justify the reported deviations and the more serious of these should normally be commented upon in the discussion or experimental section of a paper or in the "special_details" fields of the CIF. checkCIF was carefully designed to identify outliers and unusual parameters, but every test has its limitations and alerts that are not important in a particular case may appear. Conversely, the absence of alerts does not guarantee there are no aspects of the results needing attention. It is up to the individual to critically assess their own results and, if necessary, seek expert advice.

Publication of your CIF in IUCr journals

A basic structural check has been run on your CIF. These basic checks will be run on all CIFs submitted for publication in IUCr journals (*Acta Crystallographica*, *Journal of Applied Crystallography*, *Journal of Synchrotron Radiation*); however, if you intend to submit to *Acta Crystallographica Section C* or *E*, you should make sure that full publication checks are run on the final version of your CIF prior to submission.

Publication of your CIF in other journals

Please refer to the *Notes for Authors* of the relevant journal for any special instructions relating to CIF submission.

PLATON version of 21/06/2015; check.def file version of 21/06/2015

Datablock NAYPIM - ellipsoid plot

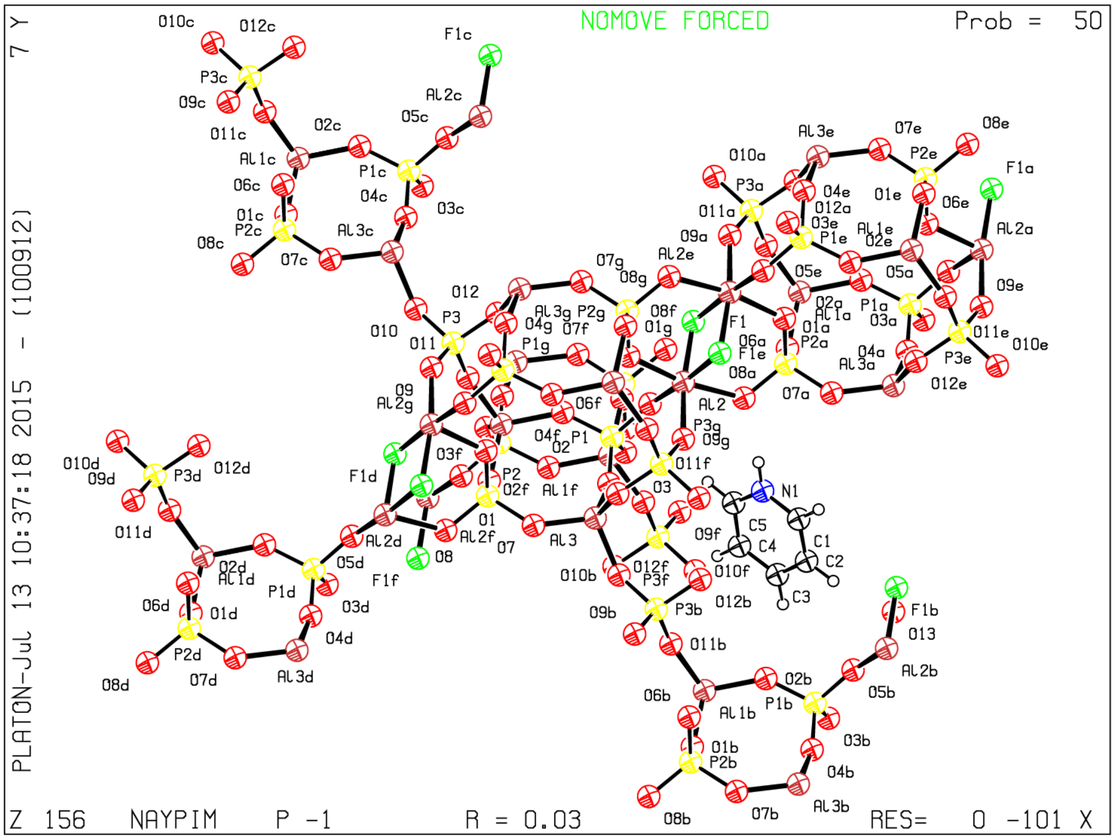

checkCIF/PLATON report

You have not supplied any structure factors. As a result the full set of tests cannot be run.

THIS REPORT IS FOR GUIDANCE ONLY. IF USED AS PART OF A REVIEW PROCEDURE FOR PUBLICATION, IT SHOULD NOT REPLACE THE EXPERTISE OF AN EXPERIENCED CRYSTALLOGRAPHIC REFEREE.

No syntax errors found. CIF dictionary Interpreting this report

Datablock: J._Solid_State_Chem._1994,_111,_427-ULM-3

```
Bond precision:  = 0.0000 A              Wavelength=0.71073

Cell:               a=10.15400       b=18.39300       c=15.77300
                    alpha=90         beta=90          gamma=90
Temperature:        0 K

                Calculated                   Reported
Volume          2945.805                     0
Space group     P b c a                      Pbca
Hall group      -P 2ac 2ab                   ?
Moiety formula  F2 Ga3 O12 P3, C3 N2, O      ?
Sum formula     C3 F2 Ga3 N2 O13 P3          ?
Mr              612.12                       0.00
Dx,g cm-3       2.760                        0.000
Z               8                            0
Mu (mm-1)       5.874                        0.000
F000            2336.0                       0.0
F000'           2345.15
h,k,lmax
Nref
Tmin,Tmax
Tmin'

Correction method= Not given

Data completeness=                 Theta(max)=

R(reflections)=                    wR2(reflections)=

S =                        Npar=
```

The following ALERTS were generated. Each ALERT has the format
test-name_ALERT_alert-type_alert-level.
Click on the hyperlinks for more details of the test.

Alert level A

```
EXPT005_ALERT_1_A  _exptl_crystal_description is missing
            Crystal habit description.
            The following tests will not be performed.
            CRYSR_01
DIFF003_ALERT_1_A  _diffrn_measurement_device_type is missing
            Diffractometer make and type. Replaces _diffrn_measurement_type.
ATOM007_ALERT_1_A  _atom_site_aniso_label is missing
            Unique label identifying the atom site.
GEOM006_ALERT_1_A  _geom_angle_atom_site_label_2 is missing
            Label identifying the atom site 2.
GEOM007_ALERT_1_A  _geom_angle_atom_site_label_3 is missing
            Label identifying the atom site 3.
PLAT029_ALERT_3_A _diffrn_measured_fraction_theta_full Low .......      0.000 Note
PLAT043_ALERT_1_A Calculated and Reported Mol. Weight Differ by ..     612.12 Check
PLAT091_ALERT_1_A No Wavelength found in CIF - 0.71073 Ang Assumed     Please Check
PLAT197_ALERT_1_A Missing            _cell_measurement_temperature     Please Suppl
PLAT198_ALERT_1_A Missing              _diffrn_ambient_temperature     Please Suppl
```

Alert level B

```
PLAT306_ALERT_2_B Isolated Oxygen Atom (H-atoms Missing ?) .......         Ow Check
```

Alert level C

```
PLAT141_ALERT_4_C su on a - Axis Small or Missing ...............    0.00000 Ang.
PLAT142_ALERT_4_C su on b - Axis Small or Missing ...............    0.00000 Ang.
PLAT143_ALERT_4_C su on c - Axis Small or Missing ...............    0.00000 Ang.
PLAT161_ALERT_4_C Missing or Zero su (esd) on x-coordinate for ...        GA1
PLAT161_ALERT_4_C Missing or Zero su (esd) on x-coordinate for ...        GA2
PLAT161_ALERT_4_C Missing or Zero su (esd) on x-coordinate for ...        GA3
PLAT161_ALERT_4_C Missing or Zero su (esd) on x-coordinate for ...         P1
PLAT161_ALERT_4_C Missing or Zero su (esd) on x-coordinate for ...         P2
PLAT161_ALERT_4_C Missing or Zero su (esd) on x-coordinate for ...         P3
PLAT161_ALERT_4_C Missing or Zero su (esd) on x-coordinate for ...         F1
PLAT161_ALERT_4_C Missing or Zero su (esd) on x-coordinate for ...         F2
PLAT161_ALERT_4_C Missing or Zero su (esd) on x-coordinate for ...         O1
PLAT161_ALERT_4_C Missing or Zero su (esd) on x-coordinate for ...         O2
PLAT161_ALERT_4_C Missing or Zero su (esd) on x-coordinate for ...         O3
PLAT161_ALERT_4_C Missing or Zero su (esd) on x-coordinate for ...         O4
PLAT161_ALERT_4_C Missing or Zero su (esd) on x-coordinate for ...         O5
PLAT161_ALERT_4_C Missing or Zero su (esd) on x-coordinate for ...         O6
PLAT161_ALERT_4_C Missing or Zero su (esd) on x-coordinate for ...         O7
PLAT161_ALERT_4_C Missing or Zero su (esd) on x-coordinate for ...         O8
PLAT161_ALERT_4_C Missing or Zero su (esd) on x-coordinate for ...         O9
PLAT161_ALERT_4_C Missing or Zero su (esd) on x-coordinate for ...        O10
PLAT161_ALERT_4_C Missing or Zero su (esd) on x-coordinate for ...        O11
PLAT161_ALERT_4_C Missing or Zero su (esd) on x-coordinate for ...        O12
PLAT161_ALERT_4_C Missing or Zero su (esd) on x-coordinate for ...         OW
PLAT161_ALERT_4_C Missing or Zero su (esd) on x-coordinate for ...         N1
PLAT161_ALERT_4_C Missing or Zero su (esd) on x-coordinate for ...         N2
PLAT161_ALERT_4_C Missing or Zero su (esd) on x-coordinate for ...         C1
PLAT161_ALERT_4_C Missing or Zero su (esd) on x-coordinate for ...         C2
PLAT161_ALERT_4_C Missing or Zero su (esd) on x-coordinate for ...         C3
PLAT162_ALERT_4_C Missing or Zero su (esd) on y-coordinate for ...        GA1
PLAT162_ALERT_4_C Missing or Zero su (esd) on y-coordinate for ...        GA2
PLAT162_ALERT_4_C Missing or Zero su (esd) on y-coordinate for ...        GA3
PLAT162_ALERT_4_C Missing or Zero su (esd) on y-coordinate for ...         P1
PLAT162_ALERT_4_C Missing or Zero su (esd) on y-coordinate for ...         P2
PLAT162_ALERT_4_C Missing or Zero su (esd) on y-coordinate for ...         P3
PLAT162_ALERT_4_C Missing or Zero su (esd) on y-coordinate for ...         F1
```

```
PLAT162_ALERT_4_C Missing or Zero su (esd) on y-coordinate for ...         F2
PLAT162_ALERT_4_C Missing or Zero su (esd) on y-coordinate for ...         O1
PLAT162_ALERT_4_C Missing or Zero su (esd) on y-coordinate for ...         O2
PLAT162_ALERT_4_C Missing or Zero su (esd) on y-coordinate for ...         O3
PLAT162_ALERT_4_C Missing or Zero su (esd) on y-coordinate for ...         O4
PLAT162_ALERT_4_C Missing or Zero su (esd) on y-coordinate for ...         O5
PLAT162_ALERT_4_C Missing or Zero su (esd) on y-coordinate for ...         O6
PLAT162_ALERT_4_C Missing or Zero su (esd) on y-coordinate for ...         O7
PLAT162_ALERT_4_C Missing or Zero su (esd) on y-coordinate for ...         O8
PLAT162_ALERT_4_C Missing or Zero su (esd) on y-coordinate for ...         O9
PLAT162_ALERT_4_C Missing or Zero su (esd) on y-coordinate for ...        O10
PLAT162_ALERT_4_C Missing or Zero su (esd) on y-coordinate for ...        O11
PLAT162_ALERT_4_C Missing or Zero su (esd) on y-coordinate for ...        O12
PLAT162_ALERT_4_C Missing or Zero su (esd) on y-coordinate for ...         OW
PLAT162_ALERT_4_C Missing or Zero su (esd) on y-coordinate for ...         N1
PLAT162_ALERT_4_C Missing or Zero su (esd) on y-coordinate for ...         N2
PLAT162_ALERT_4_C Missing or Zero su (esd) on y-coordinate for ...         C1
PLAT162_ALERT_4_C Missing or Zero su (esd) on y-coordinate for ...         C2
PLAT162_ALERT_4_C Missing or Zero su (esd) on y-coordinate for ...         C3
PLAT163_ALERT_4_C Missing or Zero su (esd) on z-coordinate for ...        GA1
PLAT163_ALERT_4_C Missing or Zero su (esd) on z-coordinate for ...        GA2
PLAT163_ALERT_4_C Missing or Zero su (esd) on z-coordinate for ...        GA3
PLAT163_ALERT_4_C Missing or Zero su (esd) on z-coordinate for ...         P1
PLAT163_ALERT_4_C Missing or Zero su (esd) on z-coordinate for ...         P2
PLAT163_ALERT_4_C Missing or Zero su (esd) on z-coordinate for ...         P3
PLAT163_ALERT_4_C Missing or Zero su (esd) on z-coordinate for ...         F1
PLAT163_ALERT_4_C Missing or Zero su (esd) on z-coordinate for ...         F2
PLAT163_ALERT_4_C Missing or Zero su (esd) on z-coordinate for ...         O1
PLAT163_ALERT_4_C Missing or Zero su (esd) on z-coordinate for ...         O2
PLAT163_ALERT_4_C Missing or Zero su (esd) on z-coordinate for ...         O3
PLAT163_ALERT_4_C Missing or Zero su (esd) on z-coordinate for ...         O4
PLAT163_ALERT_4_C Missing or Zero su (esd) on z-coordinate for ...         O5
PLAT163_ALERT_4_C Missing or Zero su (esd) on z-coordinate for ...         O6
PLAT163_ALERT_4_C Missing or Zero su (esd) on z-coordinate for ...         O7
PLAT163_ALERT_4_C Missing or Zero su (esd) on z-coordinate for ...         O8
PLAT163_ALERT_4_C Missing or Zero su (esd) on z-coordinate for ...         O9
PLAT163_ALERT_4_C Missing or Zero su (esd) on z-coordinate for ...        O10
PLAT163_ALERT_4_C Missing or Zero su (esd) on z-coordinate for ...        O11
PLAT163_ALERT_4_C Missing or Zero su (esd) on z-coordinate for ...        O12
PLAT163_ALERT_4_C Missing or Zero su (esd) on z-coordinate for ...         OW
PLAT163_ALERT_4_C Missing or Zero su (esd) on z-coordinate for ...         N1
PLAT163_ALERT_4_C Missing or Zero su (esd) on z-coordinate for ...         N2
PLAT163_ALERT_4_C Missing or Zero su (esd) on z-coordinate for ...         C1
PLAT163_ALERT_4_C Missing or Zero su (esd) on z-coordinate for ...         C2
PLAT163_ALERT_4_C Missing or Zero su (esd) on z-coordinate for ...         C3
PLAT202_ALERT_3_C Isotropic non-H Atoms in Anion/Solvent .........          6
PLAT431_ALERT_2_C Short Inter HL..A  Contact F1     ..  N1      ..       2.86 Ang.
```

Alert level G

```
PLAT004_ALERT_5_G Polymeric Structure Found with Maximum Dimension          3 Info
PLAT005_ALERT_5_G No _iucr_refine_instructions_details  in the CIF     Please Do !
PLAT040_ALERT_1_G No H-atoms in this Carbon Containing Compound ..     Please Check
PLAT045_ALERT_1_G Calculated and Reported Z Differ by ............       0.00 Ratio
PLAT194_ALERT_1_G Missing _cell_measurement_reflns_used datum ....     Please Do !
PLAT195_ALERT_1_G Missing _cell_measurement_theta_max   datum ....     Please Do !
PLAT196_ALERT_1_G Missing _cell_measurement_theta_min   datum ....     Please Do !
PLAT199_ALERT_1_G Reported _cell_measurement_temperature ..... (K)    -999999 Check
PLAT720_ALERT_4_G Number of Unusual/Non-Standard Labels ..........          1 Note
PLAT793_ALERT_4_G The Model has Chirality at P1      (Centro SPGR)          R Verify
PLAT793_ALERT_4_G The Model has Chirality at P2      (Centro SPGR)          R Verify
```

```
PLAT808_ALERT_5_G No Parseable SHELXL Style Weighting Scheme Found     Please Check
PLAT980_ALERT_1_G No Anomalous Scattering Factors Found in CIF ...     Please Check
```

```
 10 ALERT level A = Most likely a serious problem - resolve or explain
  1 ALERT level B = A potentially serious problem, consider carefully
 83 ALERT level C = Check. Ensure it is not caused by an omission or oversight
 13 ALERT level G = General information/check it is not something unexpected

 16 ALERT type 1 CIF construction/syntax error, inconsistent or missing data
  2 ALERT type 2 Indicator that the structure model may be wrong or deficient
  2 ALERT type 3 Indicator that the structure quality may be low
 84 ALERT type 4 Improvement, methodology, query or suggestion
  3 ALERT type 5 Informative message, check
```

It is advisable to attempt to resolve as many as possible of the alerts in all categories. Often the minor alerts point to easily fixed oversights, errors and omissions in your CIF or refinement strategy, so attention to these fine details can be worthwhile. In order to resolve some of the more serious problems it may be necessary to carry out additional measurements or structure refinements. However, the purpose of your study may justify the reported deviations and the more serious of these should normally be commented upon in the discussion or experimental section of a paper or in the "special_details" fields of the CIF. checkCIF was carefully designed to identify outliers and unusual parameters, but every test has its limitations and alerts that are not important in a particular case may appear. Conversely, the absence of alerts does not guarantee there are no aspects of the results needing attention. It is up to the individual to critically assess their own results and, if necessary, seek expert advice.

Publication of your CIF in IUCr journals

A basic structural check has been run on your CIF. These basic checks will be run on all CIFs submitted for publication in IUCr journals (*Acta Crystallographica*, *Journal of Applied Crystallography*, *Journal of Synchrotron Radiation*); however, if you intend to submit to *Acta Crystallographica Section C* or *E*, you should make sure that full publication checks are run on the final version of your CIF prior to submission.

Publication of your CIF in other journals

Please refer to the *Notes for Authors* of the relevant journal for any special instructions relating to CIF submission.

PLATON version of 21/06/2015; check.def file version of 21/06/2015

Datablock J._Solid_State_Chem._1994,_111,_427-ULM-3 - ellipsoid plot

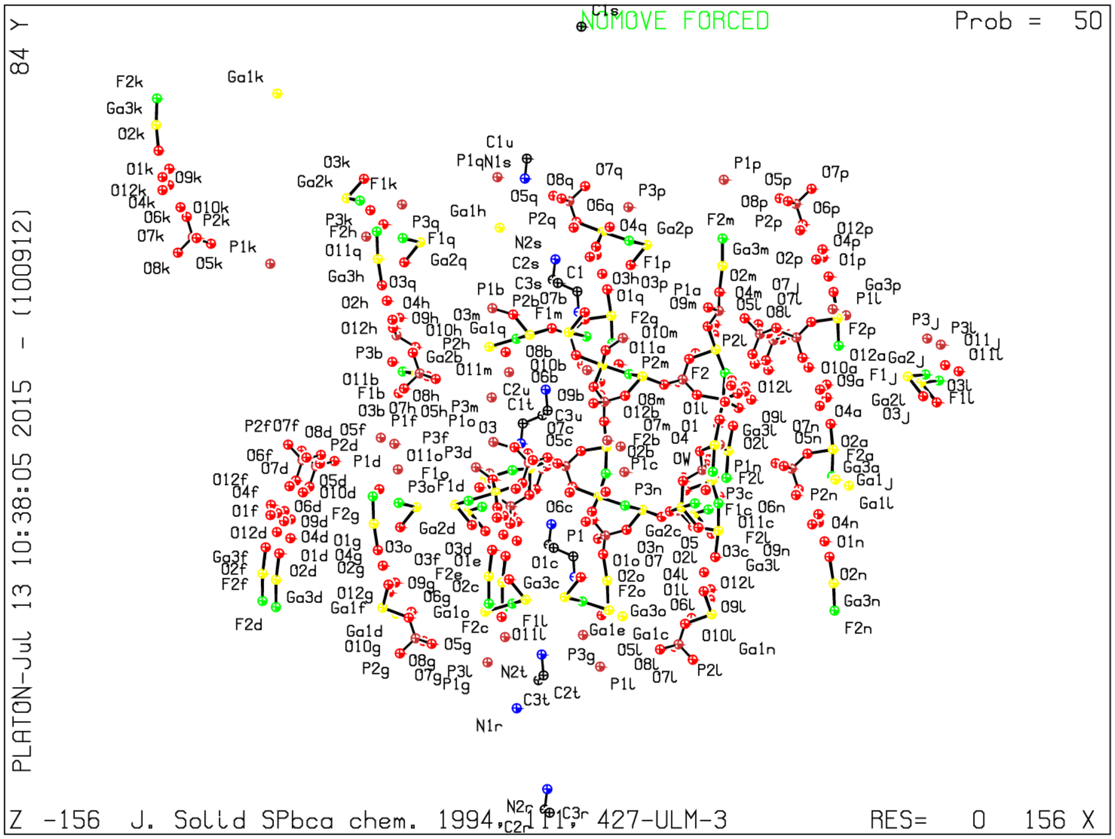

checkCIF/PLATON report

You have not supplied any structure factors. As a result the full set of tests cannot be run.

THIS REPORT IS FOR GUIDANCE ONLY. IF USED AS PART OF A REVIEW PROCEDURE FOR PUBLICATION, IT SHOULD NOT REPLACE THE EXPERTISE OF AN EXPERIENCED CRYSTALLOGRAPHIC REFEREE.

No syntax errors found. CIF dictionary Interpreting this report

Datablock: Micropor._Mater._5_1996_365-ULM-3-1

```
Bond precision:  = 0.0000 A             Wavelength=0.71073

Cell:               a=10.07500       b=18.50600       c=16.06000
                    alpha=90         beta=90          gamma=90
Temperature:        0 K

                Calculated                 Reported
Volume          2994.354                   0
Space group     P b c a                    Pbca
Hall group      -P 2ac 2ab                 ?
Moiety formula  F2 Ga3 O12 P3, C4 N3       ?
Sum formula     C4 F2 Ga3 N3 O12 P3        ?
Mr              622.14                     0.00
Dx,g cm-3       2.760                      0.000
Z               8                          0
Mu (mm-1)       5.778                      0.000
F000            2376.0                     0.0
F000'           2385.09
h,k,lmax
Nref
Tmin,Tmax
Tmin'

Correction method= Not given

Data completeness=                Theta(max)=

R(reflections)=                   wR2(reflections)=

S =                        Npar=
```

The following ALERTS were generated. Each ALERT has the format
test-name_ALERT_alert-type_alert-level.
Click on the hyperlinks for more details of the test.

Alert level A

```
EXPT005_ALERT_1_A  _exptl_crystal_description is missing
            Crystal habit description.
            The following tests will not be performed.
            CRYSR_01
DIFF003_ALERT_1_A  _diffrn_measurement_device_type is missing
            Diffractometer make and type. Replaces _diffrn_measurement_type.
ATOM007_ALERT_1_A  _atom_site_aniso_label is missing
            Unique label identifying the atom site.
GEOM006_ALERT_1_A  _geom_angle_atom_site_label_2 is missing
            Label identifying the atom site 2.
GEOM007_ALERT_1_A  _geom_angle_atom_site_label_3 is missing
            Label identifying the atom site 3.
PLAT029_ALERT_3_A _diffrn_measured_fraction_theta_full Low .......      0.000 Note
PLAT043_ALERT_1_A Calculated and Reported Mol. Weight Differ by ..     622.14 Check
PLAT091_ALERT_1_A No Wavelength found in CIF - 0.71073 Ang Assumed     Please Check
PLAT197_ALERT_1_A Missing            _cell_measurement_temperature     Please Suppl
PLAT198_ALERT_1_A Missing              _diffrn_ambient_temperature     Please Suppl
```

Alert level C

```
PLAT141_ALERT_4_C su on a - Axis Small or Missing ...............    0.00000 Ang.
PLAT142_ALERT_4_C su on b - Axis Small or Missing ...............    0.00000 Ang.
PLAT143_ALERT_4_C su on c - Axis Small or Missing ...............    0.00000 Ang.
PLAT161_ALERT_4_C Missing or Zero su (esd) on x-coordinate for ...        GA1
PLAT161_ALERT_4_C Missing or Zero su (esd) on x-coordinate for ...        GA2
PLAT161_ALERT_4_C Missing or Zero su (esd) on x-coordinate for ...        GA3
PLAT161_ALERT_4_C Missing or Zero su (esd) on x-coordinate for ...         P1
PLAT161_ALERT_4_C Missing or Zero su (esd) on x-coordinate for ...         P2
PLAT161_ALERT_4_C Missing or Zero su (esd) on x-coordinate for ...         P3
PLAT161_ALERT_4_C Missing or Zero su (esd) on x-coordinate for ...         F1
PLAT161_ALERT_4_C Missing or Zero su (esd) on x-coordinate for ...         F2
PLAT161_ALERT_4_C Missing or Zero su (esd) on x-coordinate for ...         O1
PLAT161_ALERT_4_C Missing or Zero su (esd) on x-coordinate for ...         O2
PLAT161_ALERT_4_C Missing or Zero su (esd) on x-coordinate for ...         O3
PLAT161_ALERT_4_C Missing or Zero su (esd) on x-coordinate for ...         O4
PLAT161_ALERT_4_C Missing or Zero su (esd) on x-coordinate for ...         O5
PLAT161_ALERT_4_C Missing or Zero su (esd) on x-coordinate for ...         O6
PLAT161_ALERT_4_C Missing or Zero su (esd) on x-coordinate for ...         O7
PLAT161_ALERT_4_C Missing or Zero su (esd) on x-coordinate for ...         O8
PLAT161_ALERT_4_C Missing or Zero su (esd) on x-coordinate for ...         O9
PLAT161_ALERT_4_C Missing or Zero su (esd) on x-coordinate for ...        O10
PLAT161_ALERT_4_C Missing or Zero su (esd) on x-coordinate for ...        O11
PLAT161_ALERT_4_C Missing or Zero su (esd) on x-coordinate for ...        O12
PLAT161_ALERT_4_C Missing or Zero su (esd) on x-coordinate for ...         N1
PLAT161_ALERT_4_C Missing or Zero su (esd) on x-coordinate for ...        N2A
PLAT161_ALERT_4_C Missing or Zero su (esd) on x-coordinate for ...        N2B
PLAT161_ALERT_4_C Missing or Zero su (esd) on x-coordinate for ...         C1
PLAT161_ALERT_4_C Missing or Zero su (esd) on x-coordinate for ...         C2
PLAT161_ALERT_4_C Missing or Zero su (esd) on x-coordinate for ...         C3
PLAT161_ALERT_4_C Missing or Zero su (esd) on x-coordinate for ...         C4
PLAT162_ALERT_4_C Missing or Zero su (esd) on y-coordinate for ...        GA1
PLAT162_ALERT_4_C Missing or Zero su (esd) on y-coordinate for ...        GA2
PLAT162_ALERT_4_C Missing or Zero su (esd) on y-coordinate for ...        GA3
PLAT162_ALERT_4_C Missing or Zero su (esd) on y-coordinate for ...         P1
PLAT162_ALERT_4_C Missing or Zero su (esd) on y-coordinate for ...         P2
PLAT162_ALERT_4_C Missing or Zero su (esd) on y-coordinate for ...         P3
PLAT162_ALERT_4_C Missing or Zero su (esd) on y-coordinate for ...         F1
PLAT162_ALERT_4_C Missing or Zero su (esd) on y-coordinate for ...         F2
PLAT162_ALERT_4_C Missing or Zero su (esd) on y-coordinate for ...         O1
PLAT162_ALERT_4_C Missing or Zero su (esd) on y-coordinate for ...         O2
```

```
PLAT162_ALERT_4_C Missing or Zero su (esd) on y-coordinate for ...         O3
PLAT162_ALERT_4_C Missing or Zero su (esd) on y-coordinate for ...         O4
PLAT162_ALERT_4_C Missing or Zero su (esd) on y-coordinate for ...         O5
PLAT162_ALERT_4_C Missing or Zero su (esd) on y-coordinate for ...         O6
PLAT162_ALERT_4_C Missing or Zero su (esd) on y-coordinate for ...         O7
PLAT162_ALERT_4_C Missing or Zero su (esd) on y-coordinate for ...         O8
PLAT162_ALERT_4_C Missing or Zero su (esd) on y-coordinate for ...         O9
PLAT162_ALERT_4_C Missing or Zero su (esd) on y-coordinate for ...        O10
PLAT162_ALERT_4_C Missing or Zero su (esd) on y-coordinate for ...        O11
PLAT162_ALERT_4_C Missing or Zero su (esd) on y-coordinate for ...        O12
PLAT162_ALERT_4_C Missing or Zero su (esd) on y-coordinate for ...         N1
PLAT162_ALERT_4_C Missing or Zero su (esd) on y-coordinate for ...        N2A
PLAT162_ALERT_4_C Missing or Zero su (esd) on y-coordinate for ...        N2B
PLAT162_ALERT_4_C Missing or Zero su (esd) on y-coordinate for ...         C1
PLAT162_ALERT_4_C Missing or Zero su (esd) on y-coordinate for ...         C2
PLAT162_ALERT_4_C Missing or Zero su (esd) on y-coordinate for ...         C3
PLAT162_ALERT_4_C Missing or Zero su (esd) on y-coordinate for ...         C4
PLAT163_ALERT_4_C Missing or Zero su (esd) on z-coordinate for ...        GA1
PLAT163_ALERT_4_C Missing or Zero su (esd) on z-coordinate for ...        GA2
PLAT163_ALERT_4_C Missing or Zero su (esd) on z-coordinate for ...        GA3
PLAT163_ALERT_4_C Missing or Zero su (esd) on z-coordinate for ...         P1
PLAT163_ALERT_4_C Missing or Zero su (esd) on z-coordinate for ...         P2
PLAT163_ALERT_4_C Missing or Zero su (esd) on z-coordinate for ...         P3
PLAT163_ALERT_4_C Missing or Zero su (esd) on z-coordinate for ...         F1
PLAT163_ALERT_4_C Missing or Zero su (esd) on z-coordinate for ...         F2
PLAT163_ALERT_4_C Missing or Zero su (esd) on z-coordinate for ...         O1
PLAT163_ALERT_4_C Missing or Zero su (esd) on z-coordinate for ...         O2
PLAT163_ALERT_4_C Missing or Zero su (esd) on z-coordinate for ...         O3
PLAT163_ALERT_4_C Missing or Zero su (esd) on z-coordinate for ...         O4
PLAT163_ALERT_4_C Missing or Zero su (esd) on z-coordinate for ...         O5
PLAT163_ALERT_4_C Missing or Zero su (esd) on z-coordinate for ...         O6
PLAT163_ALERT_4_C Missing or Zero su (esd) on z-coordinate for ...         O7
PLAT163_ALERT_4_C Missing or Zero su (esd) on z-coordinate for ...         O8
PLAT163_ALERT_4_C Missing or Zero su (esd) on z-coordinate for ...         O9
PLAT163_ALERT_4_C Missing or Zero su (esd) on z-coordinate for ...        O10
PLAT163_ALERT_4_C Missing or Zero su (esd) on z-coordinate for ...        O11
PLAT163_ALERT_4_C Missing or Zero su (esd) on z-coordinate for ...        O12
PLAT163_ALERT_4_C Missing or Zero su (esd) on z-coordinate for ...         N1
PLAT163_ALERT_4_C Missing or Zero su (esd) on z-coordinate for ...        N2A
PLAT163_ALERT_4_C Missing or Zero su (esd) on z-coordinate for ...        N2B
PLAT163_ALERT_4_C Missing or Zero su (esd) on z-coordinate for ...         C1
PLAT163_ALERT_4_C Missing or Zero su (esd) on z-coordinate for ...         C2
PLAT163_ALERT_4_C Missing or Zero su (esd) on z-coordinate for ...         C3
PLAT163_ALERT_4_C Missing or Zero su (esd) on z-coordinate for ...         C4
PLAT202_ALERT_3_C Isotropic non-H Atoms in Anion/Solvent .........          7
PLAT431_ALERT_2_C Short Inter HL..A  Contact F1     ..  N2A     ..       2.85 Ang.
```

Alert level G

```
PLAT004_ALERT_5_G Polymeric Structure Found with Maximum Dimension          3 Info
PLAT005_ALERT_5_G No _iucr_refine_instructions_details  in the CIF     Please Do !
PLAT040_ALERT_1_G No H-atoms in this Carbon Containing Compound ..     Please Check
PLAT045_ALERT_1_G Calculated and Reported Z Differ by ............       0.00 Ratio
PLAT194_ALERT_1_G Missing _cell_measurement_reflns_used datum ....     Please Do !
PLAT195_ALERT_1_G Missing _cell_measurement_theta_max   datum ....     Please Do !
PLAT196_ALERT_1_G Missing _cell_measurement_theta_min   datum ....     Please Do !
PLAT199_ALERT_1_G Reported _cell_measurement_temperature ..... (K)    -999999 Check
PLAT793_ALERT_4_G The Model has Chirality at P1      (Centro SPGR)          R Verify
PLAT793_ALERT_4_G The Model has Chirality at P2      (Centro SPGR)          R Verify
PLAT808_ALERT_5_G No Parseable SHELXL Style Weighting Scheme Found     Please Check
PLAT980_ALERT_1_G No Anomalous Scattering Factors Found in CIF ...     Please Check
```

```
 10 ALERT level A = Most likely a serious problem - resolve or explain
  0 ALERT level B = A potentially serious problem, consider carefully
 86 ALERT level C = Check. Ensure it is not caused by an omission or oversight
 12 ALERT level G = General information/check it is not something unexpected

 16 ALERT type 1 CIF construction/syntax error, inconsistent or missing data
  1 ALERT type 2 Indicator that the structure model may be wrong or deficient
  2 ALERT type 3 Indicator that the structure quality may be low
 86 ALERT type 4 Improvement, methodology, query or suggestion
  3 ALERT type 5 Informative message, check
```

It is advisable to attempt to resolve as many as possible of the alerts in all categories. Often the minor alerts point to easily fixed oversights, errors and omissions in your CIF or refinement strategy, so attention to these fine details can be worthwhile. In order to resolve some of the more serious problems it may be necessary to carry out additional measurements or structure refinements. However, the purpose of your study may justify the reported deviations and the more serious of these should normally be commented upon in the discussion or experimental section of a paper or in the "special_details" fields of the CIF. checkCIF was carefully designed to identify outliers and unusual parameters, but every test has its limitations and alerts that are not important in a particular case may appear. Conversely, the absence of alerts does not guarantee there are no aspects of the results needing attention. It is up to the individual to critically assess their own results and, if necessary, seek expert advice.

Publication of your CIF in IUCr journals

A basic structural check has been run on your CIF. These basic checks will be run on all CIFs submitted for publication in IUCr journals (*Acta Crystallographica*, *Journal of Applied Crystallography*, *Journal of Synchrotron Radiation*); however, if you intend to submit to *Acta Crystallographica Section C* or *E*, you should make sure that full publication checks are run on the final version of your CIF prior to submission.

Publication of your CIF in other journals

Please refer to the *Notes for Authors* of the relevant journal for any special instructions relating to CIF submission.

PLATON version of 21/06/2015; check.def file version of 21/06/2015

Datablock Micropor._Mater._5_1996_365-ULM-3-1 - ellipsoid plot

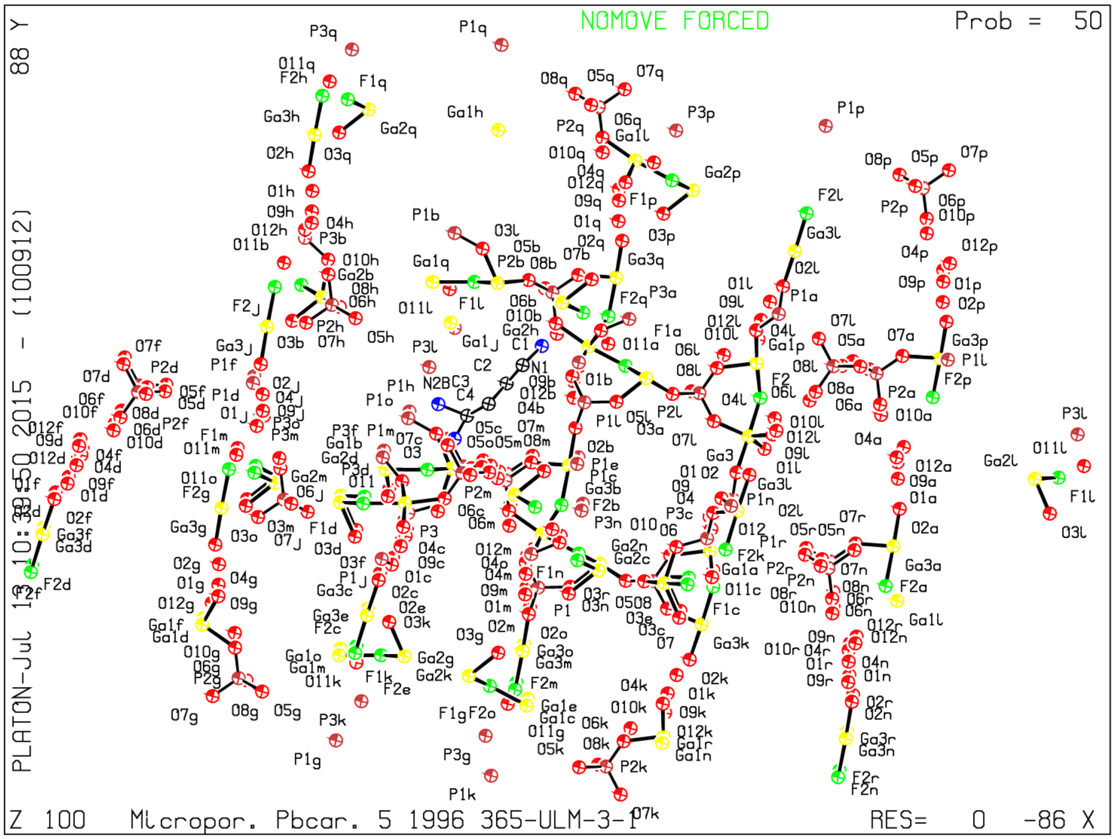

checkCIF/PLATON report

You have not supplied any structure factors. As a result the full set of tests cannot be run.

THIS REPORT IS FOR GUIDANCE ONLY. IF USED AS PART OF A REVIEW PROCEDURE FOR PUBLICATION, IT SHOULD NOT REPLACE THE EXPERTISE OF AN EXPERIENCED CRYSTALLOGRAPHIC REFEREE.

No syntax errors found. CIF dictionary Interpreting this report

Datablock: Micropor._Mater._5_1996_365-ULM-3-2

```
Bond precision:  = 0.0000 A             Wavelength=0.71073

Cell:               a=10.07500      b=18.50600      c=16.06000
                    alpha=90        beta=90         gamma=90
Temperature:        0 K

                Calculated                  Reported
Volume          2994.354                    0
Space group     P b c a                     Pbca
Hall group      -P 2ac 2ab                  ?
Moiety formula  F2 Ga3 O12 P3, C5 N3        ?
Sum formula     C5 F2 Ga3 N3 O12 P3         ?
Mr              634.15                      0.00
Dx,g cm-3       2.813                       0.000
Z               8                           0
Mu (mm-1)       5.781                       0.000
F000            2424.0                      0.0
F000’           2433.10
h,k,lmax
Nref
Tmin,Tmax
Tmin’

Correction method= Not given

Data completeness=                Theta(max)=

R(reflections)=                   wR2(reflections)=

S =                         Npar=
```

The following ALERTS were generated. Each ALERT has the format
test-name_ALERT_alert-type_alert-level.
Click on the hyperlinks for more details of the test.

Alert level A

```
EXPT005_ALERT_1_A  _exptl_crystal_description is missing
            Crystal habit description.
            The following tests will not be performed.
            CRYSR_01
DIFF003_ALERT_1_A  _diffrn_measurement_device_type is missing
            Diffractometer make and type. Replaces _diffrn_measurement_type.
ATOM007_ALERT_1_A  _atom_site_aniso_label is missing
            Unique label identifying the atom site.
GEOM006_ALERT_1_A  _geom_angle_atom_site_label_2 is missing
            Label identifying the atom site 2.
GEOM007_ALERT_1_A  _geom_angle_atom_site_label_3 is missing
            Label identifying the atom site 3.
PLAT029_ALERT_3_A _diffrn_measured_fraction_theta_full Low .......      0.000 Note
PLAT043_ALERT_1_A Calculated and Reported Mol. Weight Differ by ..     634.15 Check
PLAT091_ALERT_1_A No Wavelength found in CIF - 0.71073 Ang Assumed     Please Check
PLAT197_ALERT_1_A Missing            _cell_measurement_temperature     Please Suppl
PLAT198_ALERT_1_A Missing              _diffrn_ambient_temperature     Please Suppl
```

Alert level C

```
PLAT141_ALERT_4_C su on a - Axis Small or Missing ...............    0.00000 Ang.
PLAT142_ALERT_4_C su on b - Axis Small or Missing ...............    0.00000 Ang.
PLAT143_ALERT_4_C su on c - Axis Small or Missing ...............    0.00000 Ang.
PLAT161_ALERT_4_C Missing or Zero su (esd) on x-coordinate for ...        GA1
PLAT161_ALERT_4_C Missing or Zero su (esd) on x-coordinate for ...        GA2
PLAT161_ALERT_4_C Missing or Zero su (esd) on x-coordinate for ...        GA3
PLAT161_ALERT_4_C Missing or Zero su (esd) on x-coordinate for ...         P1
PLAT161_ALERT_4_C Missing or Zero su (esd) on x-coordinate for ...         P2
PLAT161_ALERT_4_C Missing or Zero su (esd) on x-coordinate for ...         P3
PLAT161_ALERT_4_C Missing or Zero su (esd) on x-coordinate for ...         F1
PLAT161_ALERT_4_C Missing or Zero su (esd) on x-coordinate for ...         F2
PLAT161_ALERT_4_C Missing or Zero su (esd) on x-coordinate for ...         O1
PLAT161_ALERT_4_C Missing or Zero su (esd) on x-coordinate for ...         O2
PLAT161_ALERT_4_C Missing or Zero su (esd) on x-coordinate for ...         O3
PLAT161_ALERT_4_C Missing or Zero su (esd) on x-coordinate for ...         O4
PLAT161_ALERT_4_C Missing or Zero su (esd) on x-coordinate for ...         O5
PLAT161_ALERT_4_C Missing or Zero su (esd) on x-coordinate for ...         O6
PLAT161_ALERT_4_C Missing or Zero su (esd) on x-coordinate for ...         O7
PLAT161_ALERT_4_C Missing or Zero su (esd) on x-coordinate for ...         O8
PLAT161_ALERT_4_C Missing or Zero su (esd) on x-coordinate for ...         O9
PLAT161_ALERT_4_C Missing or Zero su (esd) on x-coordinate for ...        O10
PLAT161_ALERT_4_C Missing or Zero su (esd) on x-coordinate for ...        O11
PLAT161_ALERT_4_C Missing or Zero su (esd) on x-coordinate for ...        O12
PLAT161_ALERT_4_C Missing or Zero su (esd) on x-coordinate for ...         N1
PLAT161_ALERT_4_C Missing or Zero su (esd) on x-coordinate for ...        N2A
PLAT161_ALERT_4_C Missing or Zero su (esd) on x-coordinate for ...        N2B
PLAT161_ALERT_4_C Missing or Zero su (esd) on x-coordinate for ...         C1
PLAT161_ALERT_4_C Missing or Zero su (esd) on x-coordinate for ...         C2
PLAT161_ALERT_4_C Missing or Zero su (esd) on x-coordinate for ...         C3
PLAT161_ALERT_4_C Missing or Zero su (esd) on x-coordinate for ...         C4
PLAT161_ALERT_4_C Missing or Zero su (esd) on x-coordinate for ...         C5
PLAT162_ALERT_4_C Missing or Zero su (esd) on y-coordinate for ...        GA1
PLAT162_ALERT_4_C Missing or Zero su (esd) on y-coordinate for ...        GA2
PLAT162_ALERT_4_C Missing or Zero su (esd) on y-coordinate for ...        GA3
PLAT162_ALERT_4_C Missing or Zero su (esd) on y-coordinate for ...         P1
PLAT162_ALERT_4_C Missing or Zero su (esd) on y-coordinate for ...         P2
PLAT162_ALERT_4_C Missing or Zero su (esd) on y-coordinate for ...         P3
PLAT162_ALERT_4_C Missing or Zero su (esd) on y-coordinate for ...         F1
PLAT162_ALERT_4_C Missing or Zero su (esd) on y-coordinate for ...         F2
PLAT162_ALERT_4_C Missing or Zero su (esd) on y-coordinate for ...         O1
```

```
PLAT162_ALERT_4_C Missing or Zero su (esd) on y-coordinate for ...         O2
PLAT162_ALERT_4_C Missing or Zero su (esd) on y-coordinate for ...         O3
PLAT162_ALERT_4_C Missing or Zero su (esd) on y-coordinate for ...         O4
PLAT162_ALERT_4_C Missing or Zero su (esd) on y-coordinate for ...         O5
PLAT162_ALERT_4_C Missing or Zero su (esd) on y-coordinate for ...         O6
PLAT162_ALERT_4_C Missing or Zero su (esd) on y-coordinate for ...         O7
PLAT162_ALERT_4_C Missing or Zero su (esd) on y-coordinate for ...         O8
PLAT162_ALERT_4_C Missing or Zero su (esd) on y-coordinate for ...         O9
PLAT162_ALERT_4_C Missing or Zero su (esd) on y-coordinate for ...        O10
PLAT162_ALERT_4_C Missing or Zero su (esd) on y-coordinate for ...        O11
PLAT162_ALERT_4_C Missing or Zero su (esd) on y-coordinate for ...        O12
PLAT162_ALERT_4_C Missing or Zero su (esd) on y-coordinate for ...         N1
PLAT162_ALERT_4_C Missing or Zero su (esd) on y-coordinate for ...        N2A
PLAT162_ALERT_4_C Missing or Zero su (esd) on y-coordinate for ...        N2B
PLAT162_ALERT_4_C Missing or Zero su (esd) on y-coordinate for ...         C1
PLAT162_ALERT_4_C Missing or Zero su (esd) on y-coordinate for ...         C2
PLAT162_ALERT_4_C Missing or Zero su (esd) on y-coordinate for ...         C3
PLAT162_ALERT_4_C Missing or Zero su (esd) on y-coordinate for ...         C4
PLAT162_ALERT_4_C Missing or Zero su (esd) on y-coordinate for ...         C5
PLAT163_ALERT_4_C Missing or Zero su (esd) on z-coordinate for ...        GA1
PLAT163_ALERT_4_C Missing or Zero su (esd) on z-coordinate for ...        GA2
PLAT163_ALERT_4_C Missing or Zero su (esd) on z-coordinate for ...        GA3
PLAT163_ALERT_4_C Missing or Zero su (esd) on z-coordinate for ...         P1
PLAT163_ALERT_4_C Missing or Zero su (esd) on z-coordinate for ...         P2
PLAT163_ALERT_4_C Missing or Zero su (esd) on z-coordinate for ...         P3
PLAT163_ALERT_4_C Missing or Zero su (esd) on z-coordinate for ...         F1
PLAT163_ALERT_4_C Missing or Zero su (esd) on z-coordinate for ...         F2
PLAT163_ALERT_4_C Missing or Zero su (esd) on z-coordinate for ...         O1
PLAT163_ALERT_4_C Missing or Zero su (esd) on z-coordinate for ...         O2
PLAT163_ALERT_4_C Missing or Zero su (esd) on z-coordinate for ...         O3
PLAT163_ALERT_4_C Missing or Zero su (esd) on z-coordinate for ...         O4
PLAT163_ALERT_4_C Missing or Zero su (esd) on z-coordinate for ...         O5
PLAT163_ALERT_4_C Missing or Zero su (esd) on z-coordinate for ...         O6
PLAT163_ALERT_4_C Missing or Zero su (esd) on z-coordinate for ...         O7
PLAT163_ALERT_4_C Missing or Zero su (esd) on z-coordinate for ...         O8
PLAT163_ALERT_4_C Missing or Zero su (esd) on z-coordinate for ...         O9
PLAT163_ALERT_4_C Missing or Zero su (esd) on z-coordinate for ...        O10
PLAT163_ALERT_4_C Missing or Zero su (esd) on z-coordinate for ...        O11
PLAT163_ALERT_4_C Missing or Zero su (esd) on z-coordinate for ...        O12
PLAT163_ALERT_4_C Missing or Zero su (esd) on z-coordinate for ...         N1
PLAT163_ALERT_4_C Missing or Zero su (esd) on z-coordinate for ...        N2A
PLAT163_ALERT_4_C Missing or Zero su (esd) on z-coordinate for ...        N2B
PLAT163_ALERT_4_C Missing or Zero su (esd) on z-coordinate for ...         C1
PLAT163_ALERT_4_C Missing or Zero su (esd) on z-coordinate for ...         C2
PLAT163_ALERT_4_C Missing or Zero su (esd) on z-coordinate for ...         C3
PLAT163_ALERT_4_C Missing or Zero su (esd) on z-coordinate for ...         C4
PLAT163_ALERT_4_C Missing or Zero su (esd) on z-coordinate for ...         C5
PLAT202_ALERT_3_C Isotropic non-H Atoms in Anion/Solvent .........          8
PLAT431_ALERT_2_C Short Inter HL..A  Contact F1     ..  N2B     ..       2.78 Ang.
PLAT431_ALERT_2_C Short Inter HL..A  Contact F1     ..  N1      ..       2.89 Ang.
PLAT431_ALERT_2_C Short Inter HL..A  Contact F2     ..  N1      ..       2.85 Ang.
```

Alert level G

```
PLAT004_ALERT_5_G Polymeric Structure Found with Maximum Dimension          3 Info
PLAT005_ALERT_5_G No _iucr_refine_instructions_details  in the CIF     Please Do !
PLAT040_ALERT_1_G No H-atoms in this Carbon Containing Compound ..     Please Check
PLAT045_ALERT_1_G Calculated and Reported Z Differ by ...........       0.00 Ratio
PLAT194_ALERT_1_G Missing _cell_measurement_reflns_used datum ....     Please Do !
PLAT195_ALERT_1_G Missing _cell_measurement_theta_max   datum ....     Please Do !
PLAT196_ALERT_1_G Missing _cell_measurement_theta_min   datum ....     Please Do !
```

```
PLAT199_ALERT_1_G Reported _cell_measurement_temperature ..... (K)    -999999 Check
PLAT793_ALERT_4_G The Model has Chirality at P1      (Centro SPGR)          R Verify
PLAT793_ALERT_4_G The Model has Chirality at P2      (Centro SPGR)          R Verify
PLAT808_ALERT_5_G No Parseable SHELXL Style Weighting Scheme Found     Please Check
PLAT980_ALERT_1_G No Anomalous Scattering Factors Found in CIF ...     Please Check
```

```
 10 ALERT level A = Most likely a serious problem - resolve or explain
  0 ALERT level B = A potentially serious problem, consider carefully
 91 ALERT level C = Check. Ensure it is not caused by an omission or oversight
 12 ALERT level G = General information/check it is not something unexpected

 16 ALERT type 1 CIF construction/syntax error, inconsistent or missing data
  3 ALERT type 2 Indicator that the structure model may be wrong or deficient
  2 ALERT type 3 Indicator that the structure quality may be low
 89 ALERT type 4 Improvement, methodology, query or suggestion
  3 ALERT type 5 Informative message, check
```

It is advisable to attempt to resolve as many as possible of the alerts in all categories. Often the minor alerts point to easily fixed oversights, errors and omissions in your CIF or refinement strategy, so attention to these fine details can be worthwhile. In order to resolve some of the more serious problems it may be necessary to carry out additional measurements or structure refinements. However, the purpose of your study may justify the reported deviations and the more serious of these should normally be commented upon in the discussion or experimental section of a paper or in the "special_details" fields of the CIF. checkCIF was carefully designed to identify outliers and unusual parameters, but every test has its limitations and alerts that are not important in a particular case may appear. Conversely, the absence of alerts does not guarantee there are no aspects of the results needing attention. It is up to the individual to critically assess their own results and, if necessary, seek expert advice.

Publication of your CIF in IUCr journals

A basic structural check has been run on your CIF. These basic checks will be run on all CIFs submitted for publication in IUCr journals (*Acta Crystallographica*, *Journal of Applied Crystallography*, *Journal of Synchrotron Radiation*); however, if you intend to submit to *Acta Crystallographica Section C* or *E*, you should make sure that full publication checks are run on the final version of your CIF prior to submission.

Publication of your CIF in other journals

Please refer to the *Notes for Authors* of the relevant journal for any special instructions relating to CIF submission.

PLATON version of 21/06/2015; check.def file version of 21/06/2015

Datablock Micropor._Mater._5_1996_365-ULM-3-2 - ellipsoid plot

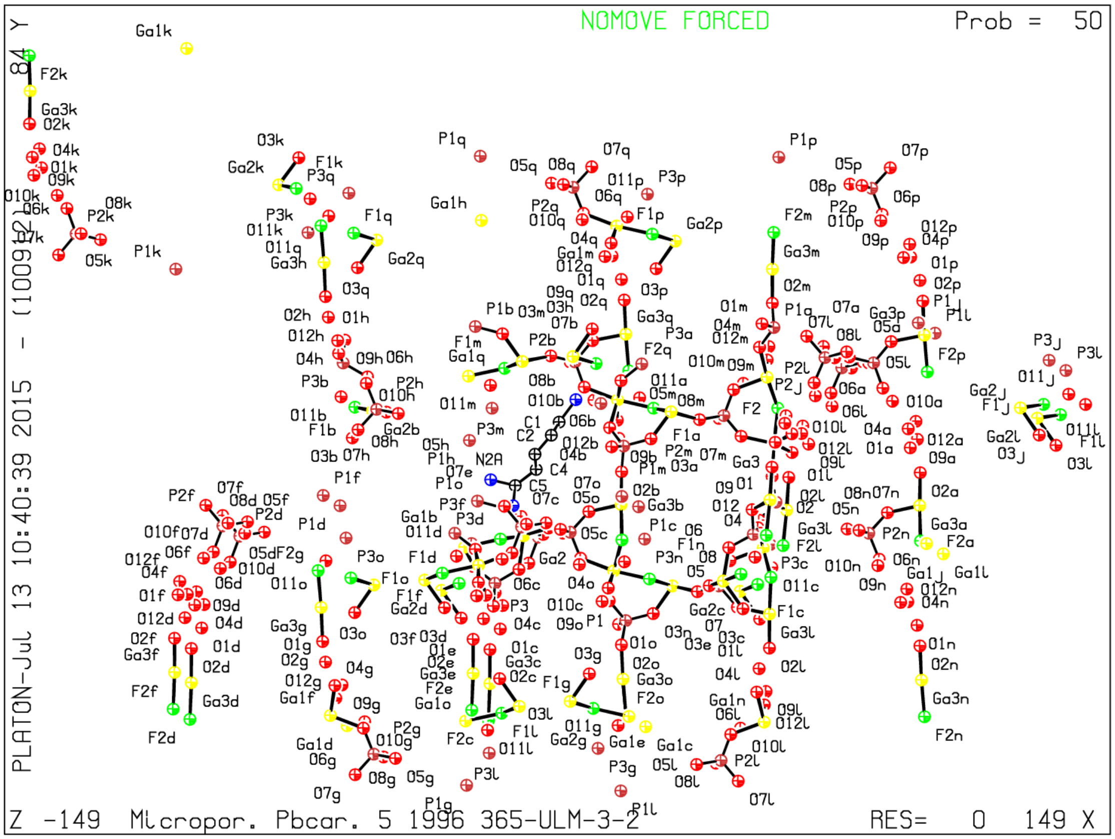

checkCIF/PLATON report

You have not supplied any structure factors. As a result the full set of tests cannot be run.

THIS REPORT IS FOR GUIDANCE ONLY. IF USED AS PART OF A REVIEW PROCEDURE FOR PUBLICATION, IT SHOULD NOT REPLACE THE EXPERTISE OF AN EXPERIENCED CRYSTALLOGRAPHIC REFEREE.

No syntax errors found. CIF dictionary Interpreting this report

Datablock: Layered_4x6x8_1,5-diaminopentane

Bond precision: = 0.0000 A Wavelength=0.71073

Cell:	a=9.80100	b=14.83700	c=17.81500
	alpha=90	beta=105.6500	gamma=90
Temperature:	0 K		

	Calculated	Reported
Volume	2494.571	0
Space group	P 21/c	P21/c
Hall group	-P 2ybc	?
Moiety formula	Al3 O16 P4, C5 H16 N2, C5 H12 N	?
Sum formula	C10 H28 Al3 N3 O16 P4	?
Mr	651.17	0.00
Dx,g cm-3	1.734	0.000
Z	4	0
Mu (mm-1)	0.487	0.000
F000	1344.0	0.0
F000’	1347.07	
h,k,lmax		
Nref		
Tmin,Tmax		
Tmin’		

Correction method= Not given

Data completeness= Theta(max)=

R(reflections)= wR2(reflections)=

S = Npar=

The following ALERTS were generated. Each ALERT has the format
test-name_ALERT_alert-type_alert-level.
Click on the hyperlinks for more details of the test.

Alert level A

```
EXPT005_ALERT_1_A  _exptl_crystal_description is missing
            Crystal habit description.
            The following tests will not be performed.
            CRYSR_01
DIFF003_ALERT_1_A  _diffrn_measurement_device_type is missing
            Diffractometer make and type. Replaces _diffrn_measurement_type.
ATOM007_ALERT_1_A  _atom_site_aniso_label is missing
            Unique label identifying the atom site.
GEOM006_ALERT_1_A  _geom_angle_atom_site_label_2 is missing
            Label identifying the atom site 2.
GEOM007_ALERT_1_A  _geom_angle_atom_site_label_3 is missing
            Label identifying the atom site 3.
PLAT029_ALERT_3_A _diffrn_measured_fraction_theta_full Low .......      0.000 Note
PLAT043_ALERT_1_A Calculated and Reported Mol. Weight Differ by ..     651.17 Check
PLAT091_ALERT_1_A No Wavelength found in CIF - 0.71073 Ang Assumed     Please Check
PLAT197_ALERT_1_A Missing            _cell_measurement_temperature     Please Suppl
PLAT198_ALERT_1_A Missing              _diffrn_ambient_temperature     Please Suppl
```

Alert level C

```
PLAT141_ALERT_4_C su on a - Axis Small or Missing ................    0.00000 Ang.
PLAT142_ALERT_4_C su on b - Axis Small or Missing ................    0.00000 Ang.
PLAT143_ALERT_4_C su on c - Axis Small or Missing ................    0.00000 Ang.
PLAT145_ALERT_4_C su on beta     Small or Missing ................     0.0000 Degree
PLAT161_ALERT_4_C Missing or Zero su (esd) on x-coordinate for ...        AL5
PLAT161_ALERT_4_C Missing or Zero su (esd) on x-coordinate for ...        AL6
PLAT161_ALERT_4_C Missing or Zero su (esd) on x-coordinate for ...        AL7
PLAT161_ALERT_4_C Missing or Zero su (esd) on x-coordinate for ...         P1
PLAT161_ALERT_4_C Missing or Zero su (esd) on x-coordinate for ...         P2
PLAT161_ALERT_4_C Missing or Zero su (esd) on x-coordinate for ...         P3
PLAT161_ALERT_4_C Missing or Zero su (esd) on x-coordinate for ...         P4
PLAT161_ALERT_4_C Missing or Zero su (esd) on x-coordinate for ...         O8
PLAT161_ALERT_4_C Missing or Zero su (esd) on x-coordinate for ...         O9
PLAT161_ALERT_4_C Missing or Zero su (esd) on x-coordinate for ...        O10
PLAT161_ALERT_4_C Missing or Zero su (esd) on x-coordinate for ...        O11
PLAT161_ALERT_4_C Missing or Zero su (esd) on x-coordinate for ...        O12
PLAT161_ALERT_4_C Missing or Zero su (esd) on x-coordinate for ...        O13
PLAT161_ALERT_4_C Missing or Zero su (esd) on x-coordinate for ...        O14
PLAT161_ALERT_4_C Missing or Zero su (esd) on x-coordinate for ...        O15
PLAT161_ALERT_4_C Missing or Zero su (esd) on x-coordinate for ...        O16
PLAT161_ALERT_4_C Missing or Zero su (esd) on x-coordinate for ...        O17
PLAT161_ALERT_4_C Missing or Zero su (esd) on x-coordinate for ...        O18
PLAT161_ALERT_4_C Missing or Zero su (esd) on x-coordinate for ...        O19
PLAT161_ALERT_4_C Missing or Zero su (esd) on x-coordinate for ...        O20
PLAT161_ALERT_4_C Missing or Zero su (esd) on x-coordinate for ...        O21
PLAT161_ALERT_4_C Missing or Zero su (esd) on x-coordinate for ...        O22
PLAT161_ALERT_4_C Missing or Zero su (esd) on x-coordinate for ...        O23
PLAT161_ALERT_4_C Missing or Zero su (esd) on x-coordinate for ...        N24
PLAT161_ALERT_4_C Missing or Zero su (esd) on x-coordinate for ...        N25
PLAT161_ALERT_4_C Missing or Zero su (esd) on x-coordinate for ...        N26
PLAT161_ALERT_4_C Missing or Zero su (esd) on x-coordinate for ...        C27
PLAT161_ALERT_4_C Missing or Zero su (esd) on x-coordinate for ...        C28
PLAT161_ALERT_4_C Missing or Zero su (esd) on x-coordinate for ...        C29
PLAT161_ALERT_4_C Missing or Zero su (esd) on x-coordinate for ...        C30
PLAT161_ALERT_4_C Missing or Zero su (esd) on x-coordinate for ...        C31
PLAT161_ALERT_4_C Missing or Zero su (esd) on x-coordinate for ...        C32
PLAT161_ALERT_4_C Missing or Zero su (esd) on x-coordinate for ...        C33
PLAT161_ALERT_4_C Missing or Zero su (esd) on x-coordinate for ...        C34
PLAT161_ALERT_4_C Missing or Zero su (esd) on x-coordinate for ...        C35
PLAT161_ALERT_4_C Missing or Zero su (esd) on x-coordinate for ...        C36
```

```
PLAT162_ALERT_4_C Missing or Zero su (esd) on y-coordinate for ...        AL5
PLAT162_ALERT_4_C Missing or Zero su (esd) on y-coordinate for ...        AL6
PLAT162_ALERT_4_C Missing or Zero su (esd) on y-coordinate for ...        AL7
PLAT162_ALERT_4_C Missing or Zero su (esd) on y-coordinate for ...         P1
PLAT162_ALERT_4_C Missing or Zero su (esd) on y-coordinate for ...         P2
PLAT162_ALERT_4_C Missing or Zero su (esd) on y-coordinate for ...         P3
PLAT162_ALERT_4_C Missing or Zero su (esd) on y-coordinate for ...         P4
PLAT162_ALERT_4_C Missing or Zero su (esd) on y-coordinate for ...         O8
PLAT162_ALERT_4_C Missing or Zero su (esd) on y-coordinate for ...         O9
PLAT162_ALERT_4_C Missing or Zero su (esd) on y-coordinate for ...        O10
PLAT162_ALERT_4_C Missing or Zero su (esd) on y-coordinate for ...        O11
PLAT162_ALERT_4_C Missing or Zero su (esd) on y-coordinate for ...        O12
PLAT162_ALERT_4_C Missing or Zero su (esd) on y-coordinate for ...        O13
PLAT162_ALERT_4_C Missing or Zero su (esd) on y-coordinate for ...        O14
PLAT162_ALERT_4_C Missing or Zero su (esd) on y-coordinate for ...        O15
PLAT162_ALERT_4_C Missing or Zero su (esd) on y-coordinate for ...        O16
PLAT162_ALERT_4_C Missing or Zero su (esd) on y-coordinate for ...        O17
PLAT162_ALERT_4_C Missing or Zero su (esd) on y-coordinate for ...        O18
PLAT162_ALERT_4_C Missing or Zero su (esd) on y-coordinate for ...        O19
PLAT162_ALERT_4_C Missing or Zero su (esd) on y-coordinate for ...        O20
PLAT162_ALERT_4_C Missing or Zero su (esd) on y-coordinate for ...        O21
PLAT162_ALERT_4_C Missing or Zero su (esd) on y-coordinate for ...        O22
PLAT162_ALERT_4_C Missing or Zero su (esd) on y-coordinate for ...        O23
PLAT162_ALERT_4_C Missing or Zero su (esd) on y-coordinate for ...        N24
PLAT162_ALERT_4_C Missing or Zero su (esd) on y-coordinate for ...        N25
PLAT162_ALERT_4_C Missing or Zero su (esd) on y-coordinate for ...        N26
PLAT162_ALERT_4_C Missing or Zero su (esd) on y-coordinate for ...        C27
PLAT162_ALERT_4_C Missing or Zero su (esd) on y-coordinate for ...        C28
PLAT162_ALERT_4_C Missing or Zero su (esd) on y-coordinate for ...        C29
PLAT162_ALERT_4_C Missing or Zero su (esd) on y-coordinate for ...        C30
PLAT162_ALERT_4_C Missing or Zero su (esd) on y-coordinate for ...        C31
PLAT162_ALERT_4_C Missing or Zero su (esd) on y-coordinate for ...        C32
PLAT162_ALERT_4_C Missing or Zero su (esd) on y-coordinate for ...        C33
PLAT162_ALERT_4_C Missing or Zero su (esd) on y-coordinate for ...        C34
PLAT162_ALERT_4_C Missing or Zero su (esd) on y-coordinate for ...        C35
PLAT162_ALERT_4_C Missing or Zero su (esd) on y-coordinate for ...        C36
PLAT163_ALERT_4_C Missing or Zero su (esd) on z-coordinate for ...        AL5
PLAT163_ALERT_4_C Missing or Zero su (esd) on z-coordinate for ...        AL6
PLAT163_ALERT_4_C Missing or Zero su (esd) on z-coordinate for ...        AL7
PLAT163_ALERT_4_C Missing or Zero su (esd) on z-coordinate for ...         P1
PLAT163_ALERT_4_C Missing or Zero su (esd) on z-coordinate for ...         P2
PLAT163_ALERT_4_C Missing or Zero su (esd) on z-coordinate for ...         P3
PLAT163_ALERT_4_C Missing or Zero su (esd) on z-coordinate for ...         P4
PLAT163_ALERT_4_C Missing or Zero su (esd) on z-coordinate for ...         O8
PLAT163_ALERT_4_C Missing or Zero su (esd) on z-coordinate for ...         O9
PLAT163_ALERT_4_C Missing or Zero su (esd) on z-coordinate for ...        O10
PLAT163_ALERT_4_C Missing or Zero su (esd) on z-coordinate for ...        O11
PLAT163_ALERT_4_C Missing or Zero su (esd) on z-coordinate for ...        O12
PLAT163_ALERT_4_C Missing or Zero su (esd) on z-coordinate for ...        O13
PLAT163_ALERT_4_C Missing or Zero su (esd) on z-coordinate for ...        O14
PLAT163_ALERT_4_C Missing or Zero su (esd) on z-coordinate for ...        O15
PLAT163_ALERT_4_C Missing or Zero su (esd) on z-coordinate for ...        O16
PLAT163_ALERT_4_C Missing or Zero su (esd) on z-coordinate for ...        O17
PLAT163_ALERT_4_C Missing or Zero su (esd) on z-coordinate for ...        O18
PLAT163_ALERT_4_C Missing or Zero su (esd) on z-coordinate for ...        O19
PLAT163_ALERT_4_C Missing or Zero su (esd) on z-coordinate for ...        O20
PLAT163_ALERT_4_C Missing or Zero su (esd) on z-coordinate for ...        O21
PLAT163_ALERT_4_C Missing or Zero su (esd) on z-coordinate for ...        O22
PLAT163_ALERT_4_C Missing or Zero su (esd) on z-coordinate for ...        O23
PLAT163_ALERT_4_C Missing or Zero su (esd) on z-coordinate for ...        N24
PLAT163_ALERT_4_C Missing or Zero su (esd) on z-coordinate for ...        N25
PLAT163_ALERT_4_C Missing or Zero su (esd) on z-coordinate for ...        N26
```

```
PLAT163_ALERT_4_C Missing or Zero su (esd) on z-coordinate for ...        C27
PLAT163_ALERT_4_C Missing or Zero su (esd) on z-coordinate for ...        C28
PLAT163_ALERT_4_C Missing or Zero su (esd) on z-coordinate for ...        C29
PLAT163_ALERT_4_C Missing or Zero su (esd) on z-coordinate for ...        C30
PLAT163_ALERT_4_C Missing or Zero su (esd) on z-coordinate for ...        C31
PLAT163_ALERT_4_C Missing or Zero su (esd) on z-coordinate for ...        C32
PLAT163_ALERT_4_C Missing or Zero su (esd) on z-coordinate for ...        C33
PLAT163_ALERT_4_C Missing or Zero su (esd) on z-coordinate for ...        C34
PLAT163_ALERT_4_C Missing or Zero su (esd) on z-coordinate for ...        C35
PLAT163_ALERT_4_C Missing or Zero su (esd) on z-coordinate for ...        C36
PLAT202_ALERT_3_C Isotropic non-H Atoms in Anion/Solvent .........         13
PLAT353_ALERT_3_C Long    N-H (N0.87,N1.01A)  N24    -   H22    ..       1.05 Ang.
PLAT353_ALERT_3_C Long    N-H (N0.87,N1.01A)  N25    -   H24    ..       1.04 Ang.
```

Alert level G

```
PLAT004_ALERT_5_G Polymeric Structure Found with Maximum Dimension          2 Info
PLAT005_ALERT_5_G No _iucr_refine_instructions_details  in the CIF     Please Do !
PLAT007_ALERT_5_G Number of Unrefined Donor-H Atoms .............           8 Report
PLAT045_ALERT_1_G Calculated and Reported Z Differ by ...........        0.00 Ratio
PLAT194_ALERT_1_G Missing _cell_measurement_reflns_used datum ....     Please Do !
PLAT195_ALERT_1_G Missing _cell_measurement_theta_max   datum ....     Please Do !
PLAT196_ALERT_1_G Missing _cell_measurement_theta_min   datum ....     Please Do !
PLAT199_ALERT_1_G Reported _cell_measurement_temperature ..... (K)    -999999 Check
PLAT793_ALERT_4_G The Model has Chirality at P1      (Centro SPGR)          R Verify
PLAT793_ALERT_4_G The Model has Chirality at P2      (Centro SPGR)          R Verify
PLAT793_ALERT_4_G The Model has Chirality at P3      (Centro SPGR)          R Verify
PLAT793_ALERT_4_G The Model has Chirality at P4      (Centro SPGR)          R Verify
PLAT794_ALERT_5_G Tentative Bond Valency for Al5     (III)   .....       3.00 Note
PLAT794_ALERT_5_G Tentative Bond Valency for Al6     (III)   .....       3.01 Note
PLAT794_ALERT_5_G Tentative Bond Valency for Al7     (III)   .....       2.95 Note
PLAT808_ALERT_5_G No Parseable SHELXL Style Weighting Scheme Found     Please Check
PLAT980_ALERT_1_G No Anomalous Scattering Factors Found in CIF ...     Please Check
```

```
 10 ALERT level A = Most likely a serious problem - resolve or explain
  0 ALERT level B = A potentially serious problem, consider carefully
115 ALERT level C = Check. Ensure it is not caused by an omission or oversight
 17 ALERT level G = General information/check it is not something unexpected

 15 ALERT type 1 CIF construction/syntax error, inconsistent or missing data
  0 ALERT type 2 Indicator that the structure model may be wrong or deficient
  4 ALERT type 3 Indicator that the structure quality may be low
116 ALERT type 4 Improvement, methodology, query or suggestion
  7 ALERT type 5 Informative message, check
```

It is advisable to attempt to resolve as many as possible of the alerts in all categories. Often the minor alerts point to easily fixed oversights, errors and omissions in your CIF or refinement strategy, so attention to these fine details can be worthwhile. In order to resolve some of the more serious problems it may be necessary to carry out additional measurements or structure refinements. However, the purpose of your study may justify the reported deviations and the more serious of these should normally be commented upon in the discussion or experimental section of a paper or in the "special_details" fields of the CIF. checkCIF was carefully designed to identify outliers and unusual parameters, but every test has its limitations and alerts that are not important in a particular case may appear. Conversely, the absence of alerts does not guarantee there are no aspects of the results needing attention. It is up to the individual to critically assess their own results and, if necessary, seek expert advice.

Publication of your CIF in IUCr journals

A basic structural check has been run on your CIF. These basic checks will be run on all CIFs submitted for publication in IUCr journals (*Acta Crystallographica*, *Journal of Applied Crystallography*, *Journal of Synchrotron Radiation*); however, if you intend to submit to *Acta Crystallographica Section C* or *E*, you should make sure that full publication checks are run on the final version of your CIF prior to submission.

Publication of your CIF in other journals

Please refer to the *Notes for Authors* of the relevant journal for any special instructions relating to CIF submission.

PLATON version of 21/06/2015; check.def file version of 21/06/2015

Datablock Layered_4x6x8_1,5-diaminopentane - ellipsoid plot

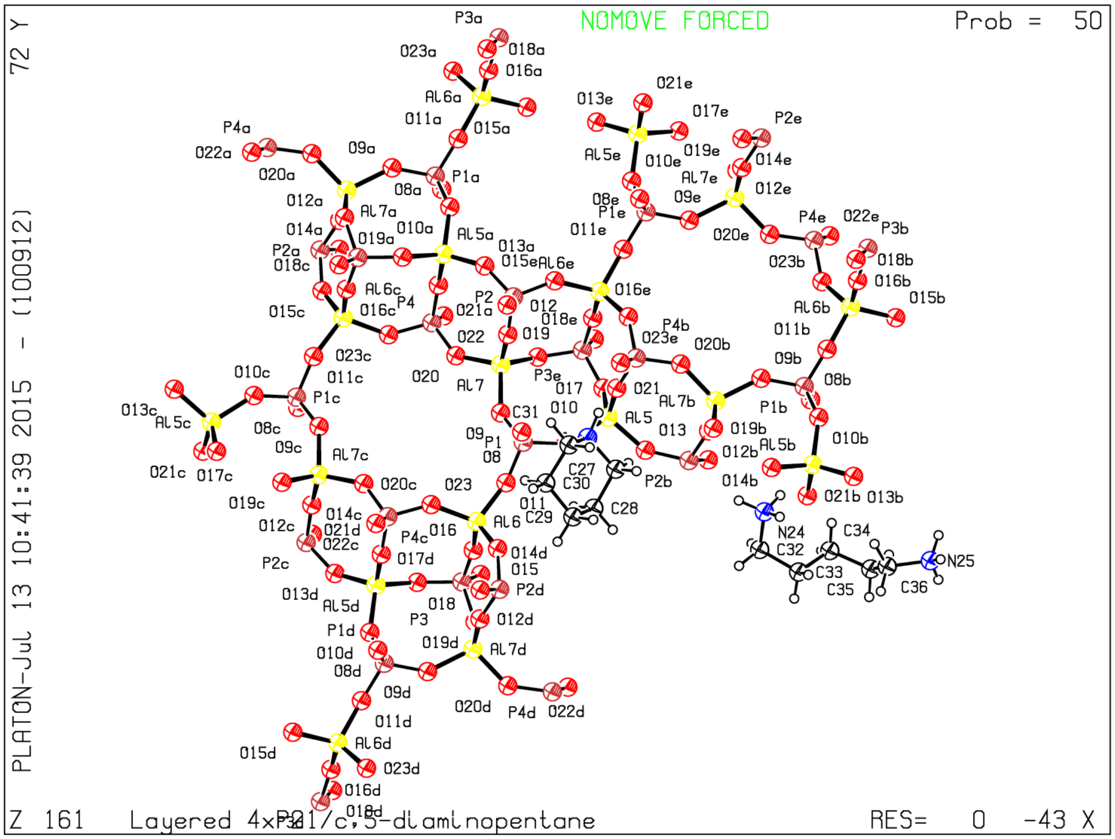

checkCIF/PLATON report

You have not supplied any structure factors. As a result the full set of tests cannot be run.

THIS REPORT IS FOR GUIDANCE ONLY. IF USED AS PART OF A REVIEW PROCEDURE FOR PUBLICATION, IT SHOULD NOT REPLACE THE EXPERTISE OF AN EXPERIENCED CRYSTALLOGRAPHIC REFEREE.

No syntax errors found. CIF dictionary Interpreting this report

Datablock: Layered_4x6x8_cyclobutylamine,_piperidine

```
Bond precision:  = 0.0000 A              Wavelength=0.71073

Cell:               a=8.99300      b=14.88400          c=9.79900
                    alpha=90       beta=103.5200       gamma=90
Temperature:        0 K

                Calculated                 Reported
Volume          1275.267                   0
Space group     P 21                       P21
Hall group      P 2yb                      ?
Moiety formula  Al3 O16 P4, C5 N, 2(C4 N)  ?
Sum formula     C13 Al3 N3 O16 P4          ?
Mr              658.98                     0.00
Dx,g cm-3       1.716                      0.000
Z               2                          0
Mu (mm-1)       0.479                      0.000
F000            652.0                      0.0
F000'           653.55
h,k,lmax
Nref
Tmin,Tmax
Tmin'

Correction method= Not given

Data completeness=                Theta(max)=

R(reflections)=                   wR2(reflections)=

S =                          Npar=
```

The following ALERTS were generated. Each ALERT has the format
test-name_ALERT_alert-type_alert-level.
Click on the hyperlinks for more details of the test.

Alert level A

```
EXPT005_ALERT_1_A  _exptl_crystal_description is missing
            Crystal habit description.
            The following tests will not be performed.
            CRYSR_01
DIFF003_ALERT_1_A  _diffrn_measurement_device_type is missing
            Diffractometer make and type. Replaces _diffrn_measurement_type.
ATOM007_ALERT_1_A  _atom_site_aniso_label is missing
            Unique label identifying the atom site.
GEOM006_ALERT_1_A  _geom_angle_atom_site_label_2 is missing
            Label identifying the atom site 2.
GEOM007_ALERT_1_A  _geom_angle_atom_site_label_3 is missing
            Label identifying the atom site 3.
PLAT029_ALERT_3_A _diffrn_measured_fraction_theta_full Low .......      0.000 Note
PLAT043_ALERT_1_A Calculated and Reported Mol. Weight Differ by ..     658.98 Check
PLAT091_ALERT_1_A No Wavelength found in CIF - 0.71073 Ang Assumed     Please Check
PLAT197_ALERT_1_A Missing            _cell_measurement_temperature     Please Suppl
PLAT198_ALERT_1_A Missing              _diffrn_ambient_temperature     Please Suppl
```

Alert level B

```
PLAT035_ALERT_1_B No _chemical_absolute_configuration info given .     Please Do !
PLAT327_ALERT_2_B Possible Missing H on sp3? Carbon .............         C30 Check
```

Alert level C

```
PLAT034_ALERT_1_C No Flack Parameter Given. Z > Si, NonCentro ....     Please Do !
PLAT141_ALERT_4_C su on a - Axis Small or Missing ...............    0.00000 Ang.
PLAT142_ALERT_4_C su on b - Axis Small or Missing ...............    0.00000 Ang.
PLAT143_ALERT_4_C su on c - Axis Small or Missing ...............    0.00000 Ang.
PLAT145_ALERT_4_C su on beta     Small or Missing ...............     0.0000 Degree
PLAT161_ALERT_4_C Missing or Zero su (esd) on x-coordinate for ...        AL1
PLAT161_ALERT_4_C Missing or Zero su (esd) on x-coordinate for ...        AL2
PLAT161_ALERT_4_C Missing or Zero su (esd) on x-coordinate for ...        AL3
PLAT161_ALERT_4_C Missing or Zero su (esd) on x-coordinate for ...         P4
PLAT161_ALERT_4_C Missing or Zero su (esd) on x-coordinate for ...         P5
PLAT161_ALERT_4_C Missing or Zero su (esd) on x-coordinate for ...         P6
PLAT161_ALERT_4_C Missing or Zero su (esd) on x-coordinate for ...         P7
PLAT161_ALERT_4_C Missing or Zero su (esd) on x-coordinate for ...         O8
PLAT161_ALERT_4_C Missing or Zero su (esd) on x-coordinate for ...         O9
PLAT161_ALERT_4_C Missing or Zero su (esd) on x-coordinate for ...        O10
PLAT161_ALERT_4_C Missing or Zero su (esd) on x-coordinate for ...        O11
PLAT161_ALERT_4_C Missing or Zero su (esd) on x-coordinate for ...        O12
PLAT161_ALERT_4_C Missing or Zero su (esd) on x-coordinate for ...        O13
PLAT161_ALERT_4_C Missing or Zero su (esd) on x-coordinate for ...        O14
PLAT161_ALERT_4_C Missing or Zero su (esd) on x-coordinate for ...        O15
PLAT161_ALERT_4_C Missing or Zero su (esd) on x-coordinate for ...        O16
PLAT161_ALERT_4_C Missing or Zero su (esd) on x-coordinate for ...        O17
PLAT161_ALERT_4_C Missing or Zero su (esd) on x-coordinate for ...        O18
PLAT161_ALERT_4_C Missing or Zero su (esd) on x-coordinate for ...        O19
PLAT161_ALERT_4_C Missing or Zero su (esd) on x-coordinate for ...        O20
PLAT161_ALERT_4_C Missing or Zero su (esd) on x-coordinate for ...        O21
PLAT161_ALERT_4_C Missing or Zero su (esd) on x-coordinate for ...        O22
PLAT161_ALERT_4_C Missing or Zero su (esd) on x-coordinate for ...        O23
PLAT161_ALERT_4_C Missing or Zero su (esd) on x-coordinate for ...        N24
PLAT161_ALERT_4_C Missing or Zero su (esd) on x-coordinate for ...        N29
PLAT161_ALERT_4_C Missing or Zero su (esd) on x-coordinate for ...        N34
PLAT161_ALERT_4_C Missing or Zero su (esd) on x-coordinate for ...        C25
PLAT161_ALERT_4_C Missing or Zero su (esd) on x-coordinate for ...        C26
PLAT161_ALERT_4_C Missing or Zero su (esd) on x-coordinate for ...        C27
PLAT161_ALERT_4_C Missing or Zero su (esd) on x-coordinate for ...        C28
```

```
PLAT161_ALERT_4_C Missing or Zero su (esd) on x-coordinate for ...        C30
PLAT161_ALERT_4_C Missing or Zero su (esd) on x-coordinate for ...        C31
PLAT161_ALERT_4_C Missing or Zero su (esd) on x-coordinate for ...        C32
PLAT161_ALERT_4_C Missing or Zero su (esd) on x-coordinate for ...        C33
PLAT161_ALERT_4_C Missing or Zero su (esd) on x-coordinate for ...        C35
PLAT161_ALERT_4_C Missing or Zero su (esd) on x-coordinate for ...        C36
PLAT161_ALERT_4_C Missing or Zero su (esd) on x-coordinate for ...        C37
PLAT161_ALERT_4_C Missing or Zero su (esd) on x-coordinate for ...        C38
PLAT161_ALERT_4_C Missing or Zero su (esd) on x-coordinate for ...        C39
PLAT162_ALERT_4_C Missing or Zero su (esd) on y-coordinate for ...        AL1
PLAT162_ALERT_4_C Missing or Zero su (esd) on y-coordinate for ...        AL2
PLAT162_ALERT_4_C Missing or Zero su (esd) on y-coordinate for ...        AL3
PLAT162_ALERT_4_C Missing or Zero su (esd) on y-coordinate for ...         P4
PLAT162_ALERT_4_C Missing or Zero su (esd) on y-coordinate for ...         P5
PLAT162_ALERT_4_C Missing or Zero su (esd) on y-coordinate for ...         P6
PLAT162_ALERT_4_C Missing or Zero su (esd) on y-coordinate for ...         P7
PLAT162_ALERT_4_C Missing or Zero su (esd) on y-coordinate for ...         O8
PLAT162_ALERT_4_C Missing or Zero su (esd) on y-coordinate for ...         O9
PLAT162_ALERT_4_C Missing or Zero su (esd) on y-coordinate for ...        O10
PLAT162_ALERT_4_C Missing or Zero su (esd) on y-coordinate for ...        O11
PLAT162_ALERT_4_C Missing or Zero su (esd) on y-coordinate for ...        O12
PLAT162_ALERT_4_C Missing or Zero su (esd) on y-coordinate for ...        O13
PLAT162_ALERT_4_C Missing or Zero su (esd) on y-coordinate for ...        O14
PLAT162_ALERT_4_C Missing or Zero su (esd) on y-coordinate for ...        O15
PLAT162_ALERT_4_C Missing or Zero su (esd) on y-coordinate for ...        O16
PLAT162_ALERT_4_C Missing or Zero su (esd) on y-coordinate for ...        O17
PLAT162_ALERT_4_C Missing or Zero su (esd) on y-coordinate for ...        O18
PLAT162_ALERT_4_C Missing or Zero su (esd) on y-coordinate for ...        O19
PLAT162_ALERT_4_C Missing or Zero su (esd) on y-coordinate for ...        O20
PLAT162_ALERT_4_C Missing or Zero su (esd) on y-coordinate for ...        O21
PLAT162_ALERT_4_C Missing or Zero su (esd) on y-coordinate for ...        O22
PLAT162_ALERT_4_C Missing or Zero su (esd) on y-coordinate for ...        O23
PLAT162_ALERT_4_C Missing or Zero su (esd) on y-coordinate for ...        N24
PLAT162_ALERT_4_C Missing or Zero su (esd) on y-coordinate for ...        N29
PLAT162_ALERT_4_C Missing or Zero su (esd) on y-coordinate for ...        N34
PLAT162_ALERT_4_C Missing or Zero su (esd) on y-coordinate for ...        C25
PLAT162_ALERT_4_C Missing or Zero su (esd) on y-coordinate for ...        C26
PLAT162_ALERT_4_C Missing or Zero su (esd) on y-coordinate for ...        C27
PLAT162_ALERT_4_C Missing or Zero su (esd) on y-coordinate for ...        C28
PLAT162_ALERT_4_C Missing or Zero su (esd) on y-coordinate for ...        C30
PLAT162_ALERT_4_C Missing or Zero su (esd) on y-coordinate for ...        C31
PLAT162_ALERT_4_C Missing or Zero su (esd) on y-coordinate for ...        C32
PLAT162_ALERT_4_C Missing or Zero su (esd) on y-coordinate for ...        C33
PLAT162_ALERT_4_C Missing or Zero su (esd) on y-coordinate for ...        C35
PLAT162_ALERT_4_C Missing or Zero su (esd) on y-coordinate for ...        C36
PLAT162_ALERT_4_C Missing or Zero su (esd) on y-coordinate for ...        C37
PLAT162_ALERT_4_C Missing or Zero su (esd) on y-coordinate for ...        C38
PLAT162_ALERT_4_C Missing or Zero su (esd) on y-coordinate for ...        C39
PLAT163_ALERT_4_C Missing or Zero su (esd) on z-coordinate for ...        AL1
PLAT163_ALERT_4_C Missing or Zero su (esd) on z-coordinate for ...        AL2
PLAT163_ALERT_4_C Missing or Zero su (esd) on z-coordinate for ...        AL3
PLAT163_ALERT_4_C Missing or Zero su (esd) on z-coordinate for ...         P4
PLAT163_ALERT_4_C Missing or Zero su (esd) on z-coordinate for ...         P5
PLAT163_ALERT_4_C Missing or Zero su (esd) on z-coordinate for ...         P6
PLAT163_ALERT_4_C Missing or Zero su (esd) on z-coordinate for ...         P7
PLAT163_ALERT_4_C Missing or Zero su (esd) on z-coordinate for ...         O8
PLAT163_ALERT_4_C Missing or Zero su (esd) on z-coordinate for ...         O9
PLAT163_ALERT_4_C Missing or Zero su (esd) on z-coordinate for ...        O10
PLAT163_ALERT_4_C Missing or Zero su (esd) on z-coordinate for ...        O11
PLAT163_ALERT_4_C Missing or Zero su (esd) on z-coordinate for ...        O12
PLAT163_ALERT_4_C Missing or Zero su (esd) on z-coordinate for ...        O13
PLAT163_ALERT_4_C Missing or Zero su (esd) on z-coordinate for ...        O14
```

```
PLAT163_ALERT_4_C Missing or Zero su (esd) on z-coordinate for ...        O15
PLAT163_ALERT_4_C Missing or Zero su (esd) on z-coordinate for ...        O16
PLAT163_ALERT_4_C Missing or Zero su (esd) on z-coordinate for ...        O17
PLAT163_ALERT_4_C Missing or Zero su (esd) on z-coordinate for ...        O18
PLAT163_ALERT_4_C Missing or Zero su (esd) on z-coordinate for ...        O19
PLAT163_ALERT_4_C Missing or Zero su (esd) on z-coordinate for ...        O20
PLAT163_ALERT_4_C Missing or Zero su (esd) on z-coordinate for ...        O21
PLAT163_ALERT_4_C Missing or Zero su (esd) on z-coordinate for ...        O22
PLAT163_ALERT_4_C Missing or Zero su (esd) on z-coordinate for ...        O23
PLAT163_ALERT_4_C Missing or Zero su (esd) on z-coordinate for ...        N24
PLAT163_ALERT_4_C Missing or Zero su (esd) on z-coordinate for ...        N29
PLAT163_ALERT_4_C Missing or Zero su (esd) on z-coordinate for ...        N34
PLAT163_ALERT_4_C Missing or Zero su (esd) on z-coordinate for ...        C25
PLAT163_ALERT_4_C Missing or Zero su (esd) on z-coordinate for ...        C26
PLAT163_ALERT_4_C Missing or Zero su (esd) on z-coordinate for ...        C27
PLAT163_ALERT_4_C Missing or Zero su (esd) on z-coordinate for ...        C28
PLAT163_ALERT_4_C Missing or Zero su (esd) on z-coordinate for ...        C30
PLAT163_ALERT_4_C Missing or Zero su (esd) on z-coordinate for ...        C31
PLAT163_ALERT_4_C Missing or Zero su (esd) on z-coordinate for ...        C32
PLAT163_ALERT_4_C Missing or Zero su (esd) on z-coordinate for ...        C33
PLAT163_ALERT_4_C Missing or Zero su (esd) on z-coordinate for ...        C35
PLAT163_ALERT_4_C Missing or Zero su (esd) on z-coordinate for ...        C36
PLAT163_ALERT_4_C Missing or Zero su (esd) on z-coordinate for ...        C37
PLAT163_ALERT_4_C Missing or Zero su (esd) on z-coordinate for ...        C38
PLAT163_ALERT_4_C Missing or Zero su (esd) on z-coordinate for ...        C39
PLAT202_ALERT_3_C Isotropic non-H Atoms in Anion/Solvent .........         16
PLAT243_ALERT_4_C High  'Solvent' Ueq as Compared to Neighbors of         C36 Check
PLAT243_ALERT_4_C High  'Solvent' Ueq as Compared to Neighbors of         C26 Check
PLAT243_ALERT_4_C High  'Solvent' Ueq as Compared to Neighbors of         C28 Check
PLAT243_ALERT_4_C High  'Solvent' Ueq as Compared to Neighbors of         C32 Check
PLAT244_ALERT_4_C Low   'Solvent' Ueq as Compared to Neighbors of         C27 Check
PLAT244_ALERT_4_C Low   'Solvent' Ueq as Compared to Neighbors of         C30 Check
PLAT244_ALERT_4_C Low   'Solvent' Ueq as Compared to Neighbors of         C31 Check
```

Alert level G

```
PLAT004_ALERT_5_G Polymeric Structure Found with Maximum Dimension          2 Info
PLAT005_ALERT_5_G No _iucr_refine_instructions_details  in the CIF     Please Do !
PLAT040_ALERT_1_G No H-atoms in this Carbon Containing Compound ..     Please Check
PLAT045_ALERT_1_G Calculated and Reported Z Differ by ............       0.00 Ratio
PLAT194_ALERT_1_G Missing _cell_measurement_reflns_used datum ....     Please Do !
PLAT195_ALERT_1_G Missing _cell_measurement_theta_max   datum ....     Please Do !
PLAT196_ALERT_1_G Missing _cell_measurement_theta_min   datum ....     Please Do !
PLAT199_ALERT_1_G Reported _cell_measurement_temperature ..... (K)    -999999 Check
PLAT432_ALERT_2_G Short Inter X...Y Contact  C26    ..  C33     ..       3.14 Ang.
PLAT773_ALERT_2_G Check long C-C Bond in CIF: C25    --  C28     .       1.70 Ang.
PLAT773_ALERT_2_G Check long C-C Bond in CIF: C26    --  C27     .       1.72 Ang.
PLAT791_ALERT_4_G The Model has Chirality at P4      (Chiral SPGR)          R Verify
PLAT791_ALERT_4_G The Model has Chirality at P5      (Chiral SPGR)          S Verify
PLAT791_ALERT_4_G The Model has Chirality at P6      (Chiral SPGR)          S Verify
PLAT791_ALERT_4_G The Model has Chirality at P7      (Chiral SPGR)          S Verify
PLAT794_ALERT_5_G Tentative Bond Valency for Al1     (III)   .....       2.98 Note
PLAT794_ALERT_5_G Tentative Bond Valency for Al2     (III)   .....       2.94 Note
PLAT794_ALERT_5_G Tentative Bond Valency for Al3     (III)   .....       2.94 Note
PLAT808_ALERT_5_G No Parseable SHELXL Style Weighting Scheme Found     Please Check
PLAT980_ALERT_1_G No Anomalous Scattering Factors Found in CIF ...     Please Check
```

---

10 **ALERT level A** = Most likely a serious problem - resolve or explain
2 **ALERT level B** = A potentially serious problem, consider carefully
130 **ALERT level C** = Check. Ensure it is not caused by an omission or oversight
20 **ALERT level G** = General information/check it is not something unexpected

```
 18 ALERT type 1 CIF construction/syntax error, inconsistent or missing data
  4 ALERT type 2 Indicator that the structure model may be wrong or deficient
  2 ALERT type 3 Indicator that the structure quality may be low
132 ALERT type 4 Improvement, methodology, query or suggestion
  6 ALERT type 5 Informative message, check
```

It is advisable to attempt to resolve as many as possible of the alerts in all categories. Often the minor alerts point to easily fixed oversights, errors and omissions in your CIF or refinement strategy, so attention to these fine details can be worthwhile. In order to resolve some of the more serious problems it may be necessary to carry out additional measurements or structure refinements. However, the purpose of your study may justify the reported deviations and the more serious of these should normally be commented upon in the discussion or experimental section of a paper or in the "special_details" fields of the CIF. checkCIF was carefully designed to identify outliers and unusual parameters, but every test has its limitations and alerts that are not important in a particular case may appear. Conversely, the absence of alerts does not guarantee there are no aspects of the results needing attention. It is up to the individual to critically assess their own results and, if necessary, seek expert advice.

Publication of your CIF in IUCr journals

A basic structural check has been run on your CIF. These basic checks will be run on all CIFs submitted for publication in IUCr journals (*Acta Crystallographica*, *Journal of Applied Crystallography*, *Journal of Synchrotron Radiation*); however, if you intend to submit to *Acta Crystallographica Section C* or *E*, you should make sure that full publication checks are run on the final version of your CIF prior to submission.

Publication of your CIF in other journals

Please refer to the *Notes for Authors* of the relevant journal for any special instructions relating to CIF submission.

PLATON version of 21/06/2015; check.def file version of 21/06/2015

Datablock Layered_4x6x8_cyclobutylamine,_piperidine - ellipsoid plot

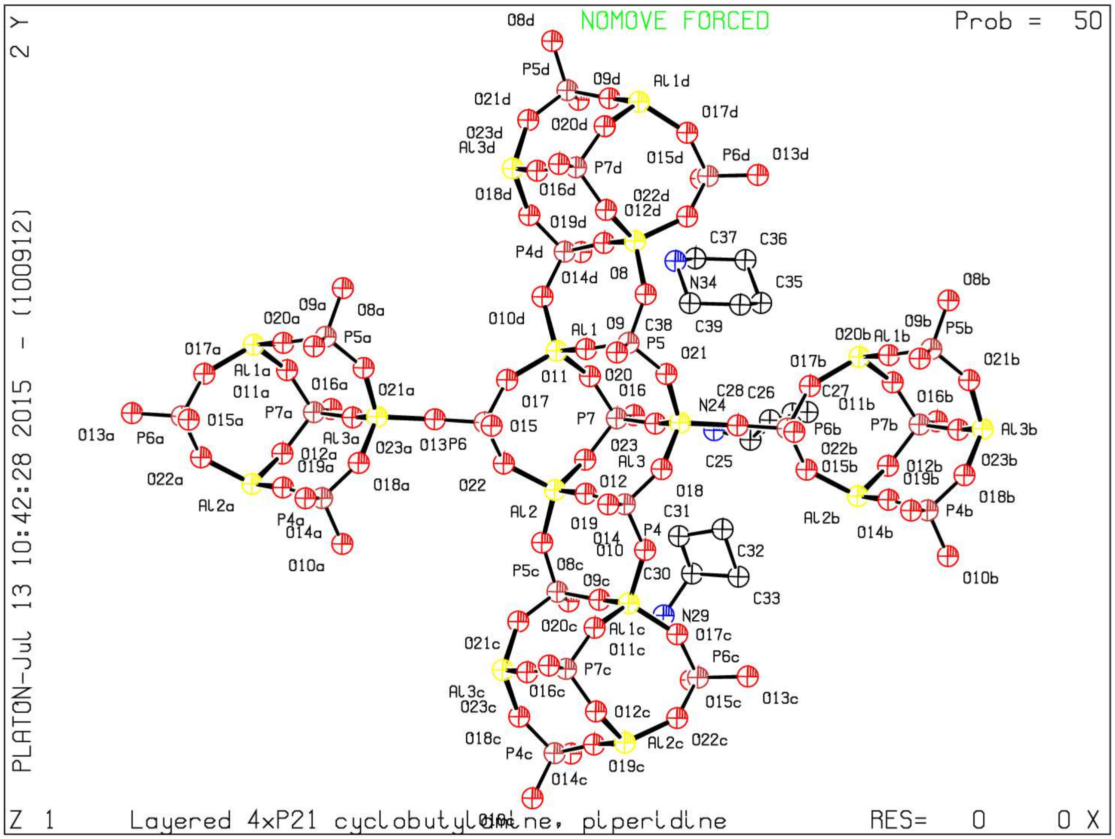

checkCIF/PLATON report

You have not supplied any structure factors. As a result the full set of tests cannot be run.

THIS REPORT IS FOR GUIDANCE ONLY. IF USED AS PART OF A REVIEW PROCEDURE FOR PUBLICATION, IT SHOULD NOT REPLACE THE EXPERTISE OF AN EXPERIENCED CRYSTALLOGRAPHIC REFEREE.

No syntax errors found. CIF dictionary Interpreting this report

Datablock: Layered_4x6x8_etylyamine

```
Bond precision:  = 0.0000 A              Wavelength=0.71073

Cell:              a=8.92000      b=14.89600          c=9.36300
                   alpha=90       beta=106.0700       gamma=90
Temperature:       0 K

                Calculated                 Reported
Volume          1195.470                   0
Space group     P 21/m                     P21/m
Hall group      -P 2yb                     ?
Moiety formula  Al3 O16 P4, 3(C2 H8 N)     ?
Sum formula     C6 H24 Al3 N3 O16 P4       ?
Mr              599.10                     0.00
Dx,g cm-3       1.664                      0.000
Z               2                          0
Mu (mm-1)       0.500                      0.000
F000            616.0                      0.0
F000'           617.52
h,k,lmax
Nref
Tmin,Tmax
Tmin'

Correction method= Not given

Data completeness=                Theta(max)=

R(reflections)=                   wR2(reflections)=

S =                        Npar=
```

The following ALERTS were generated. Each ALERT has the format
test-name_ALERT_alert-type_alert-level.
Click on the hyperlinks for more details of the test.

Alert level A

```
EXPT005_ALERT_1_A  _exptl_crystal_description is missing
            Crystal habit description.
            The following tests will not be performed.
            CRYSR_01
DIFF003_ALERT_1_A  _diffrn_measurement_device_type is missing
            Diffractometer make and type. Replaces _diffrn_measurement_type.
ATOM007_ALERT_1_A  _atom_site_aniso_label is missing
            Unique label identifying the atom site.
GEOM006_ALERT_1_A  _geom_angle_atom_site_label_2 is missing
            Label identifying the atom site 2.
GEOM007_ALERT_1_A  _geom_angle_atom_site_label_3 is missing
            Label identifying the atom site 3.
PLAT029_ALERT_3_A _diffrn_measured_fraction_theta_full Low .......      0.000 Note
PLAT043_ALERT_1_A Calculated and Reported Mol. Weight Differ by ..     599.10 Check
PLAT091_ALERT_1_A No Wavelength found in CIF - 0.71073 Ang Assumed     Please Check
PLAT197_ALERT_1_A Missing            _cell_measurement_temperature     Please Suppl
PLAT198_ALERT_1_A Missing              _diffrn_ambient_temperature     Please Suppl
```

Alert level B

```
PLAT360_ALERT_2_B Short  C(sp3)-C(sp3) Bond   C20    -   C21    ..        1.29 Ang.
```

Alert level C

```
PLAT141_ALERT_4_C su on a - Axis Small or Missing ...............    0.00000 Ang.
PLAT142_ALERT_4_C su on b - Axis Small or Missing ...............    0.00000 Ang.
PLAT143_ALERT_4_C su on c - Axis Small or Missing ...............    0.00000 Ang.
PLAT145_ALERT_4_C su on beta     Small or Missing ...............     0.0000 Degree
PLAT161_ALERT_4_C Missing or Zero su (esd) on x-coordinate for ...        AL5
PLAT161_ALERT_4_C Missing or Zero su (esd) on x-coordinate for ...         P3
PLAT161_ALERT_4_C Missing or Zero su (esd) on x-coordinate for ...         O8
PLAT161_ALERT_4_C Missing or Zero su (esd) on x-coordinate for ...        O11
PLAT161_ALERT_4_C Missing or Zero su (esd) on x-coordinate for ...        O12
PLAT161_ALERT_4_C Missing or Zero su (esd) on x-coordinate for ...        O13
PLAT161_ALERT_4_C Missing or Zero su (esd) on x-coordinate for ...        O14
PLAT161_ALERT_4_C Missing or Zero su (esd) on x-coordinate for ...        O15
PLAT161_ALERT_4_C Missing or Zero su (esd) on x-coordinate for ...        N19
PLAT161_ALERT_4_C Missing or Zero su (esd) on x-coordinate for ...        C20
PLAT161_ALERT_4_C Missing or Zero su (esd) on x-coordinate for ...        C21
PLAT162_ALERT_4_C Missing or Zero su (esd) on y-coordinate for ...        AL5
PLAT162_ALERT_4_C Missing or Zero su (esd) on y-coordinate for ...         P3
PLAT162_ALERT_4_C Missing or Zero su (esd) on y-coordinate for ...         O8
PLAT162_ALERT_4_C Missing or Zero su (esd) on y-coordinate for ...        O11
PLAT162_ALERT_4_C Missing or Zero su (esd) on y-coordinate for ...        O12
PLAT162_ALERT_4_C Missing or Zero su (esd) on y-coordinate for ...        O13
PLAT162_ALERT_4_C Missing or Zero su (esd) on y-coordinate for ...        O14
PLAT162_ALERT_4_C Missing or Zero su (esd) on y-coordinate for ...        O15
PLAT162_ALERT_4_C Missing or Zero su (esd) on y-coordinate for ...        N19
PLAT162_ALERT_4_C Missing or Zero su (esd) on y-coordinate for ...        C20
PLAT162_ALERT_4_C Missing or Zero su (esd) on y-coordinate for ...        C21
PLAT163_ALERT_4_C Missing or Zero su (esd) on z-coordinate for ...        AL5
PLAT163_ALERT_4_C Missing or Zero su (esd) on z-coordinate for ...         P3
PLAT163_ALERT_4_C Missing or Zero su (esd) on z-coordinate for ...         O8
PLAT163_ALERT_4_C Missing or Zero su (esd) on z-coordinate for ...        O11
PLAT163_ALERT_4_C Missing or Zero su (esd) on z-coordinate for ...        O12
PLAT163_ALERT_4_C Missing or Zero su (esd) on z-coordinate for ...        O13
PLAT163_ALERT_4_C Missing or Zero su (esd) on z-coordinate for ...        O14
PLAT163_ALERT_4_C Missing or Zero su (esd) on z-coordinate for ...        O15
PLAT163_ALERT_4_C Missing or Zero su (esd) on z-coordinate for ...        N19
PLAT163_ALERT_4_C Missing or Zero su (esd) on z-coordinate for ...        C20
```

```
PLAT163_ALERT_4_C Missing or Zero su (esd) on z-coordinate for ...        C21
PLAT202_ALERT_3_C Isotropic non-H Atoms in Anion/Solvent .........          6
PLAT241_ALERT_2_C High      Ueq as Compared to Neighbors for .....         O9 Check
PLAT241_ALERT_2_C High      Ueq as Compared to Neighbors for .....        O13 Check
PLAT242_ALERT_2_C Low       Ueq as Compared to Neighbors for .....        Al4 Check
PLAT244_ALERT_4_C Low   'Solvent' Ueq as Compared to Neighbors of         C17 Check
PLAT244_ALERT_4_C Low   'Solvent' Ueq as Compared to Neighbors of         C20 Check
PLAT353_ALERT_3_C Long    N-H (N0.87,N1.01A)  N19    -   H29    ..       1.02 Ang.
```

Alert level G

```
PLAT004_ALERT_5_G Polymeric Structure Found with Maximum Dimension          2 Info
PLAT005_ALERT_5_G No _iucr_refine_instructions_details  in the CIF     Please Do !
PLAT007_ALERT_5_G Number of Unrefined Donor-H Atoms ..............          5 Report
PLAT045_ALERT_1_G Calculated and Reported Z Differ by ............       0.00 Ratio
PLAT194_ALERT_1_G Missing _cell_measurement_reflns_used datum ....     Please Do !
PLAT195_ALERT_1_G Missing _cell_measurement_theta_max   datum ....     Please Do !
PLAT196_ALERT_1_G Missing _cell_measurement_theta_min   datum ....     Please Do !
PLAT199_ALERT_1_G Reported _cell_measurement_temperature ..... (K)    -999999 Check
PLAT794_ALERT_5_G Tentative Bond Valency for Al4     (III)   .....       2.97 Note
PLAT794_ALERT_5_G Tentative Bond Valency for Al5     (III)   .....       2.98 Note
PLAT808_ALERT_5_G No Parseable SHELXL Style Weighting Scheme Found     Please Check
PLAT980_ALERT_1_G No Anomalous Scattering Factors Found in CIF ...     Please Check
```

---

10 **ALERT level A** = Most likely a serious problem - resolve or explain
1 **ALERT level B** = A potentially serious problem, consider carefully
44 **ALERT level C** = Check. Ensure it is not caused by an omission or oversight
12 **ALERT level G** = General information/check it is not something unexpected

15 ALERT type 1 CIF construction/syntax error, inconsistent or missing data
4 ALERT type 2 Indicator that the structure model may be wrong or deficient
3 ALERT type 3 Indicator that the structure quality may be low
39 ALERT type 4 Improvement, methodology, query or suggestion
6 ALERT type 5 Informative message, check

It is advisable to attempt to resolve as many as possible of the alerts in all categories. Often the minor alerts point to easily fixed oversights, errors and omissions in your CIF or refinement strategy, so attention to these fine details can be worthwhile. In order to resolve some of the more serious problems it may be necessary to carry out additional measurements or structure refinements. However, the purpose of your study may justify the reported deviations and the more serious of these should normally be commented upon in the discussion or experimental section of a paper or in the "special_details" fields of the CIF. checkCIF was carefully designed to identify outliers and unusual parameters, but every test has its limitations and alerts that are not important in a particular case may appear. Conversely, the absence of alerts does not guarantee there are no aspects of the results needing attention. It is up to the individual to critically assess their own results and, if necessary, seek expert advice.

Publication of your CIF in IUCr journals

A basic structural check has been run on your CIF. These basic checks will be run on all CIFs submitted for publication in IUCr journals (*Acta Crystallographica*, *Journal of Applied Crystallography*, *Journal of Synchrotron Radiation*); however, if you intend to submit to *Acta Crystallographica Section C* or *E*, you should make sure that full publication checks are run on the final version of your CIF prior to submission.

Publication of your CIF in other journals

Please refer to the *Notes for Authors* of the relevant journal for any special instructions relating to CIF submission.

PLATON version of 21/06/2015; check.def file version of 21/06/2015

Datablock Layered_4x6x8_etylyamine - ellipsoid plot

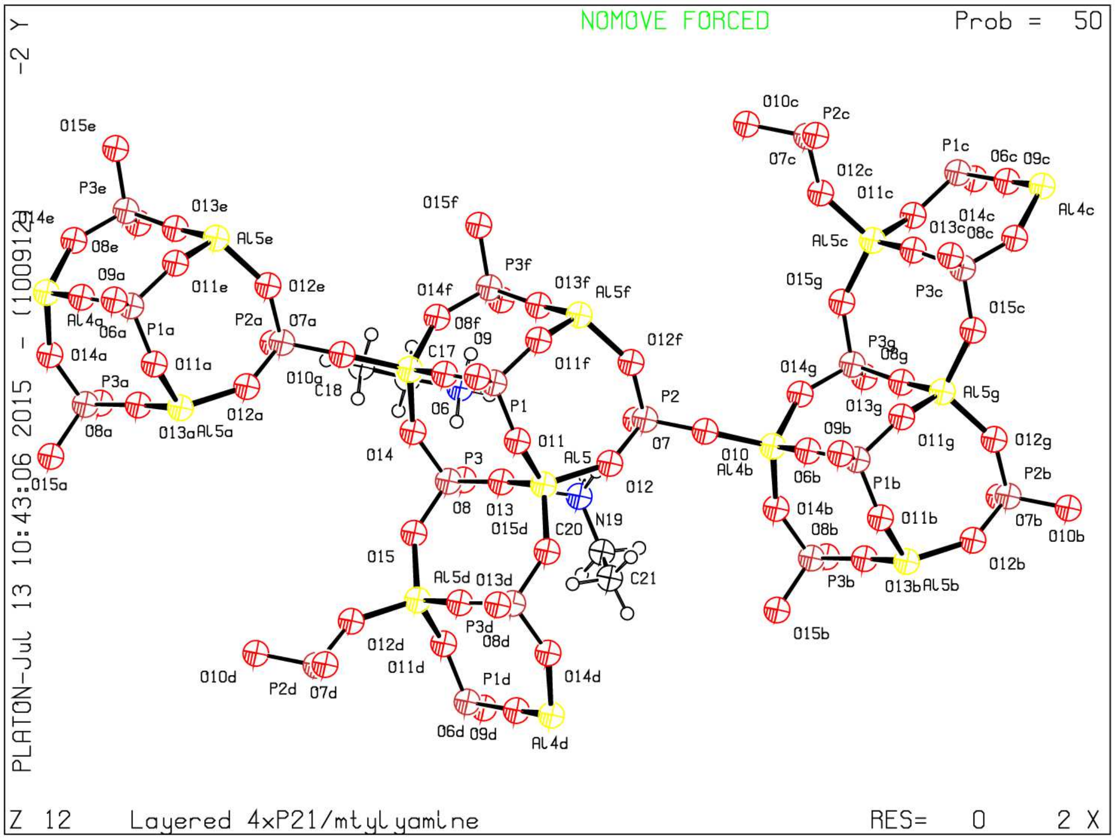

checkCIF/PLATON report

You have not supplied any structure factors. As a result the full set of tests cannot be run.

THIS REPORT IS FOR GUIDANCE ONLY. IF USED AS PART OF A REVIEW PROCEDURE FOR PUBLICATION, IT SHOULD NOT REPLACE THE EXPERTISE OF AN EXPERIENCED CRYSTALLOGRAPHIC REFEREE.

No syntax errors found.   CIF dictionary   Interpreting this report

Datablock: Layered_4x6x8_n-propylamine

```
Bond precision:  = 0.0000 A             Wavelength=0.71073

Cell:              a=11.31000      b=14.85400         c=18.62360
                   alpha=90        beta=127.3941      gamma=90
Temperature:       0 K

                Calculated                   Reported
Volume          2485.714                     0
Space group     P 21/c                       P21/c
Hall group      -P 2ybc                      ?
Moiety formula  Al3 O16 P4, 3(C3 N)          ?
Sum formula     C9 Al3 N3 O16 P4             ?
Mr              610.94                       0.00
Dx,g cm-3       1.633                        0.000
Z               4                            0
Mu (mm-1)       0.484                        0.000
F000            1208.0                       0.0
F000'           1211.07
h,k,lmax
Nref
Tmin,Tmax
Tmin'

Correction method= Not given

Data completeness=                Theta(max)=

R(reflections)=                   wR2(reflections)=

S =                        Npar=
```

The following ALERTS were generated. Each ALERT has the format
test-name_ALERT_alert-type_alert-level.
Click on the hyperlinks for more details of the test.

Alert level A

```
EXPT005_ALERT_1_A  _exptl_crystal_description is missing
            Crystal habit description.
            The following tests will not be performed.
            CRYSR_01
DIFF003_ALERT_1_A  _diffrn_measurement_device_type is missing
            Diffractometer make and type. Replaces _diffrn_measurement_type.
ATOM007_ALERT_1_A  _atom_site_aniso_label is missing
            Unique label identifying the atom site.
GEOM006_ALERT_1_A  _geom_angle_atom_site_label_2 is missing
            Label identifying the atom site 2.
GEOM007_ALERT_1_A  _geom_angle_atom_site_label_3 is missing
            Label identifying the atom site 3.
PLAT029_ALERT_3_A _diffrn_measured_fraction_theta_full Low .......      0.000 Note
PLAT043_ALERT_1_A Calculated and Reported Mol. Weight Differ by ..     610.94 Check
PLAT091_ALERT_1_A No Wavelength found in CIF - 0.71073 Ang Assumed     Please Check
PLAT197_ALERT_1_A Missing            _cell_measurement_temperature     Please Suppl
PLAT198_ALERT_1_A Missing              _diffrn_ambient_temperature     Please Suppl
```

Alert level C

```
PLAT141_ALERT_4_C su on a - Axis Small or Missing ................    0.00000 Ang.
PLAT142_ALERT_4_C su on b - Axis Small or Missing ................    0.00000 Ang.
PLAT143_ALERT_4_C su on c - Axis Small or Missing ................    0.00000 Ang.
PLAT145_ALERT_4_C su on beta     Small or Missing ................     0.0000 Degree
PLAT161_ALERT_4_C Missing or Zero su (esd) on x-coordinate for ...        AL5
PLAT161_ALERT_4_C Missing or Zero su (esd) on x-coordinate for ...        AL6
PLAT161_ALERT_4_C Missing or Zero su (esd) on x-coordinate for ...        AL7
PLAT161_ALERT_4_C Missing or Zero su (esd) on x-coordinate for ...         P1
PLAT161_ALERT_4_C Missing or Zero su (esd) on x-coordinate for ...         P2
PLAT161_ALERT_4_C Missing or Zero su (esd) on x-coordinate for ...         P3
PLAT161_ALERT_4_C Missing or Zero su (esd) on x-coordinate for ...         P4
PLAT161_ALERT_4_C Missing or Zero su (esd) on x-coordinate for ...         O8
PLAT161_ALERT_4_C Missing or Zero su (esd) on x-coordinate for ...         O9
PLAT161_ALERT_4_C Missing or Zero su (esd) on x-coordinate for ...        O10
PLAT161_ALERT_4_C Missing or Zero su (esd) on x-coordinate for ...        O11
PLAT161_ALERT_4_C Missing or Zero su (esd) on x-coordinate for ...        O12
PLAT161_ALERT_4_C Missing or Zero su (esd) on x-coordinate for ...        O13
PLAT161_ALERT_4_C Missing or Zero su (esd) on x-coordinate for ...        O14
PLAT161_ALERT_4_C Missing or Zero su (esd) on x-coordinate for ...        O15
PLAT161_ALERT_4_C Missing or Zero su (esd) on x-coordinate for ...        O16
PLAT161_ALERT_4_C Missing or Zero su (esd) on x-coordinate for ...        O17
PLAT161_ALERT_4_C Missing or Zero su (esd) on x-coordinate for ...        O18
PLAT161_ALERT_4_C Missing or Zero su (esd) on x-coordinate for ...        O19
PLAT161_ALERT_4_C Missing or Zero su (esd) on x-coordinate for ...        O20
PLAT161_ALERT_4_C Missing or Zero su (esd) on x-coordinate for ...        O21
PLAT161_ALERT_4_C Missing or Zero su (esd) on x-coordinate for ...        O22
PLAT161_ALERT_4_C Missing or Zero su (esd) on x-coordinate for ...        O23
PLAT161_ALERT_4_C Missing or Zero su (esd) on x-coordinate for ...        N24
PLAT161_ALERT_4_C Missing or Zero su (esd) on x-coordinate for ...        N28
PLAT161_ALERT_4_C Missing or Zero su (esd) on x-coordinate for ...        N32
PLAT161_ALERT_4_C Missing or Zero su (esd) on x-coordinate for ...        C25
PLAT161_ALERT_4_C Missing or Zero su (esd) on x-coordinate for ...        C26
PLAT161_ALERT_4_C Missing or Zero su (esd) on x-coordinate for ...        C27
PLAT161_ALERT_4_C Missing or Zero su (esd) on x-coordinate for ...        C29
PLAT161_ALERT_4_C Missing or Zero su (esd) on x-coordinate for ...        C30
PLAT161_ALERT_4_C Missing or Zero su (esd) on x-coordinate for ...        C31
PLAT161_ALERT_4_C Missing or Zero su (esd) on x-coordinate for ...        C33
PLAT161_ALERT_4_C Missing or Zero su (esd) on x-coordinate for ...        C34
PLAT161_ALERT_4_C Missing or Zero su (esd) on x-coordinate for ...        C35
PLAT162_ALERT_4_C Missing or Zero su (esd) on y-coordinate for ...        AL5
```

```
PLAT162_ALERT_4_C Missing or Zero su (esd) on y-coordinate for ...        AL6
PLAT162_ALERT_4_C Missing or Zero su (esd) on y-coordinate for ...        AL7
PLAT162_ALERT_4_C Missing or Zero su (esd) on y-coordinate for ...         P1
PLAT162_ALERT_4_C Missing or Zero su (esd) on y-coordinate for ...         P2
PLAT162_ALERT_4_C Missing or Zero su (esd) on y-coordinate for ...         P3
PLAT162_ALERT_4_C Missing or Zero su (esd) on y-coordinate for ...         P4
PLAT162_ALERT_4_C Missing or Zero su (esd) on y-coordinate for ...         O8
PLAT162_ALERT_4_C Missing or Zero su (esd) on y-coordinate for ...         O9
PLAT162_ALERT_4_C Missing or Zero su (esd) on y-coordinate for ...        O10
PLAT162_ALERT_4_C Missing or Zero su (esd) on y-coordinate for ...        O11
PLAT162_ALERT_4_C Missing or Zero su (esd) on y-coordinate for ...        O12
PLAT162_ALERT_4_C Missing or Zero su (esd) on y-coordinate for ...        O13
PLAT162_ALERT_4_C Missing or Zero su (esd) on y-coordinate for ...        O14
PLAT162_ALERT_4_C Missing or Zero su (esd) on y-coordinate for ...        O15
PLAT162_ALERT_4_C Missing or Zero su (esd) on y-coordinate for ...        O16
PLAT162_ALERT_4_C Missing or Zero su (esd) on y-coordinate for ...        O17
PLAT162_ALERT_4_C Missing or Zero su (esd) on y-coordinate for ...        O18
PLAT162_ALERT_4_C Missing or Zero su (esd) on y-coordinate for ...        O19
PLAT162_ALERT_4_C Missing or Zero su (esd) on y-coordinate for ...        O20
PLAT162_ALERT_4_C Missing or Zero su (esd) on y-coordinate for ...        O21
PLAT162_ALERT_4_C Missing or Zero su (esd) on y-coordinate for ...        O22
PLAT162_ALERT_4_C Missing or Zero su (esd) on y-coordinate for ...        O23
PLAT162_ALERT_4_C Missing or Zero su (esd) on y-coordinate for ...        N24
PLAT162_ALERT_4_C Missing or Zero su (esd) on y-coordinate for ...        N28
PLAT162_ALERT_4_C Missing or Zero su (esd) on y-coordinate for ...        N32
PLAT162_ALERT_4_C Missing or Zero su (esd) on y-coordinate for ...        C25
PLAT162_ALERT_4_C Missing or Zero su (esd) on y-coordinate for ...        C26
PLAT162_ALERT_4_C Missing or Zero su (esd) on y-coordinate for ...        C27
PLAT162_ALERT_4_C Missing or Zero su (esd) on y-coordinate for ...        C29
PLAT162_ALERT_4_C Missing or Zero su (esd) on y-coordinate for ...        C30
PLAT162_ALERT_4_C Missing or Zero su (esd) on y-coordinate for ...        C31
PLAT162_ALERT_4_C Missing or Zero su (esd) on y-coordinate for ...        C33
PLAT162_ALERT_4_C Missing or Zero su (esd) on y-coordinate for ...        C34
PLAT162_ALERT_4_C Missing or Zero su (esd) on y-coordinate for ...        C35
PLAT163_ALERT_4_C Missing or Zero su (esd) on z-coordinate for ...        AL5
PLAT163_ALERT_4_C Missing or Zero su (esd) on z-coordinate for ...        AL6
PLAT163_ALERT_4_C Missing or Zero su (esd) on z-coordinate for ...        AL7
PLAT163_ALERT_4_C Missing or Zero su (esd) on z-coordinate for ...         P1
PLAT163_ALERT_4_C Missing or Zero su (esd) on z-coordinate for ...         P2
PLAT163_ALERT_4_C Missing or Zero su (esd) on z-coordinate for ...         P3
PLAT163_ALERT_4_C Missing or Zero su (esd) on z-coordinate for ...         P4
PLAT163_ALERT_4_C Missing or Zero su (esd) on z-coordinate for ...         O8
PLAT163_ALERT_4_C Missing or Zero su (esd) on z-coordinate for ...         O9
PLAT163_ALERT_4_C Missing or Zero su (esd) on z-coordinate for ...        O10
PLAT163_ALERT_4_C Missing or Zero su (esd) on z-coordinate for ...        O11
PLAT163_ALERT_4_C Missing or Zero su (esd) on z-coordinate for ...        O12
PLAT163_ALERT_4_C Missing or Zero su (esd) on z-coordinate for ...        O13
PLAT163_ALERT_4_C Missing or Zero su (esd) on z-coordinate for ...        O14
PLAT163_ALERT_4_C Missing or Zero su (esd) on z-coordinate for ...        O15
PLAT163_ALERT_4_C Missing or Zero su (esd) on z-coordinate for ...        O16
PLAT163_ALERT_4_C Missing or Zero su (esd) on z-coordinate for ...        O17
PLAT163_ALERT_4_C Missing or Zero su (esd) on z-coordinate for ...        O18
PLAT163_ALERT_4_C Missing or Zero su (esd) on z-coordinate for ...        O19
PLAT163_ALERT_4_C Missing or Zero su (esd) on z-coordinate for ...        O20
PLAT163_ALERT_4_C Missing or Zero su (esd) on z-coordinate for ...        O21
PLAT163_ALERT_4_C Missing or Zero su (esd) on z-coordinate for ...        O22
PLAT163_ALERT_4_C Missing or Zero su (esd) on z-coordinate for ...        O23
PLAT163_ALERT_4_C Missing or Zero su (esd) on z-coordinate for ...        N24
PLAT163_ALERT_4_C Missing or Zero su (esd) on z-coordinate for ...        N28
PLAT163_ALERT_4_C Missing or Zero su (esd) on z-coordinate for ...        N32
PLAT163_ALERT_4_C Missing or Zero su (esd) on z-coordinate for ...        C25
PLAT163_ALERT_4_C Missing or Zero su (esd) on z-coordinate for ...        C26
```

```
PLAT163_ALERT_4_C Missing or Zero su (esd) on z-coordinate for ...        C27
PLAT163_ALERT_4_C Missing or Zero su (esd) on z-coordinate for ...        C29
PLAT163_ALERT_4_C Missing or Zero su (esd) on z-coordinate for ...        C30
PLAT163_ALERT_4_C Missing or Zero su (esd) on z-coordinate for ...        C31
PLAT163_ALERT_4_C Missing or Zero su (esd) on z-coordinate for ...        C33
PLAT163_ALERT_4_C Missing or Zero su (esd) on z-coordinate for ...        C34
PLAT163_ALERT_4_C Missing or Zero su (esd) on z-coordinate for ...        C35
PLAT202_ALERT_3_C Isotropic non-H Atoms in Anion/Solvent .........         12
PLAT243_ALERT_4_C High  'Solvent' Ueq as Compared to Neighbors of         C25 Check
PLAT244_ALERT_4_C Low   'Solvent' Ueq as Compared to Neighbors of         C34 Check
```

Alert level G

```
PLAT004_ALERT_5_G Polymeric Structure Found with Maximum Dimension          3 Info
PLAT005_ALERT_5_G No _iucr_refine_instructions_details  in the CIF     Please Do !
PLAT040_ALERT_1_G No H-atoms in this Carbon Containing Compound ..     Please Check
PLAT045_ALERT_1_G Calculated and Reported Z Differ by ............       0.00 Ratio
PLAT128_ALERT_4_G Alternate Setting for Input Space Group  P21/c        P21/n Note
PLAT194_ALERT_1_G Missing _cell_measurement_reflns_used datum ....     Please Do !
PLAT195_ALERT_1_G Missing _cell_measurement_theta_max   datum ....     Please Do !
PLAT196_ALERT_1_G Missing _cell_measurement_theta_min   datum ....     Please Do !
PLAT199_ALERT_1_G Reported _cell_measurement_temperature ..... (K)    -999999 Check
PLAT432_ALERT_2_G Short Inter X...Y Contact  O8     ..  C33     ..       2.97 Ang.
PLAT793_ALERT_4_G The Model has Chirality at P2      (Centro SPGR)          S Verify
PLAT793_ALERT_4_G The Model has Chirality at P3      (Centro SPGR)          R Verify
PLAT793_ALERT_4_G The Model has Chirality at P4      (Centro SPGR)          S Verify
PLAT794_ALERT_5_G Tentative Bond Valency for Al6     (III)   .....       2.50 Note
PLAT794_ALERT_5_G Tentative Bond Valency for Al7     (III)   .....       2.98 Note
PLAT808_ALERT_5_G No Parseable SHELXL Style Weighting Scheme Found     Please Check
PLAT980_ALERT_1_G No Anomalous Scattering Factors Found in CIF ...     Please Check
```

---

10 **ALERT level A** = Most likely a serious problem - resolve or explain
0 **ALERT level B** = A potentially serious problem, consider carefully
112 **ALERT level C** = Check. Ensure it is not caused by an omission or oversight
17 **ALERT level G** = General information/check it is not something unexpected

16 ALERT type 1 CIF construction/syntax error, inconsistent or missing data
1 ALERT type 2 Indicator that the structure model may be wrong or deficient
2 ALERT type 3 Indicator that the structure quality may be low
115 ALERT type 4 Improvement, methodology, query or suggestion
5 ALERT type 5 Informative message, check

It is advisable to attempt to resolve as many as possible of the alerts in all categories. Often the minor alerts point to easily fixed oversights, errors and omissions in your CIF or refinement strategy, so attention to these fine details can be worthwhile. In order to resolve some of the more serious problems it may be necessary to carry out additional measurements or structure refinements. However, the purpose of your study may justify the reported deviations and the more serious of these should normally be commented upon in the discussion or experimental section of a paper or in the "special_details" fields of the CIF. checkCIF was carefully designed to identify outliers and unusual parameters, but every test has its limitations and alerts that are not important in a particular case may appear. Conversely, the absence of alerts does not guarantee there are no aspects of the results needing attention. It is up to the individual to critically assess their own results and, if necessary, seek expert advice.

Publication of your CIF in IUCr journals

A basic structural check has been run on your CIF. These basic checks will be run on all CIFs submitted for publication in IUCr journals (*Acta Crystallographica*, *Journal of Applied Crystallography*, *Journal of Synchrotron Radiation*); however, if you intend to submit to *Acta Crystallographica Section C* or *E*, you should make sure that full publication checks are run on the final version of your CIF prior to submission.

Publication of your CIF in other journals

Please refer to the *Notes for Authors* of the relevant journal for any special instructions relating to CIF submission.

PLATON version of 21/06/2015; check.def file version of 21/06/2015

Datablock Layered_4x6x8_n-propylamine - ellipsoid plot

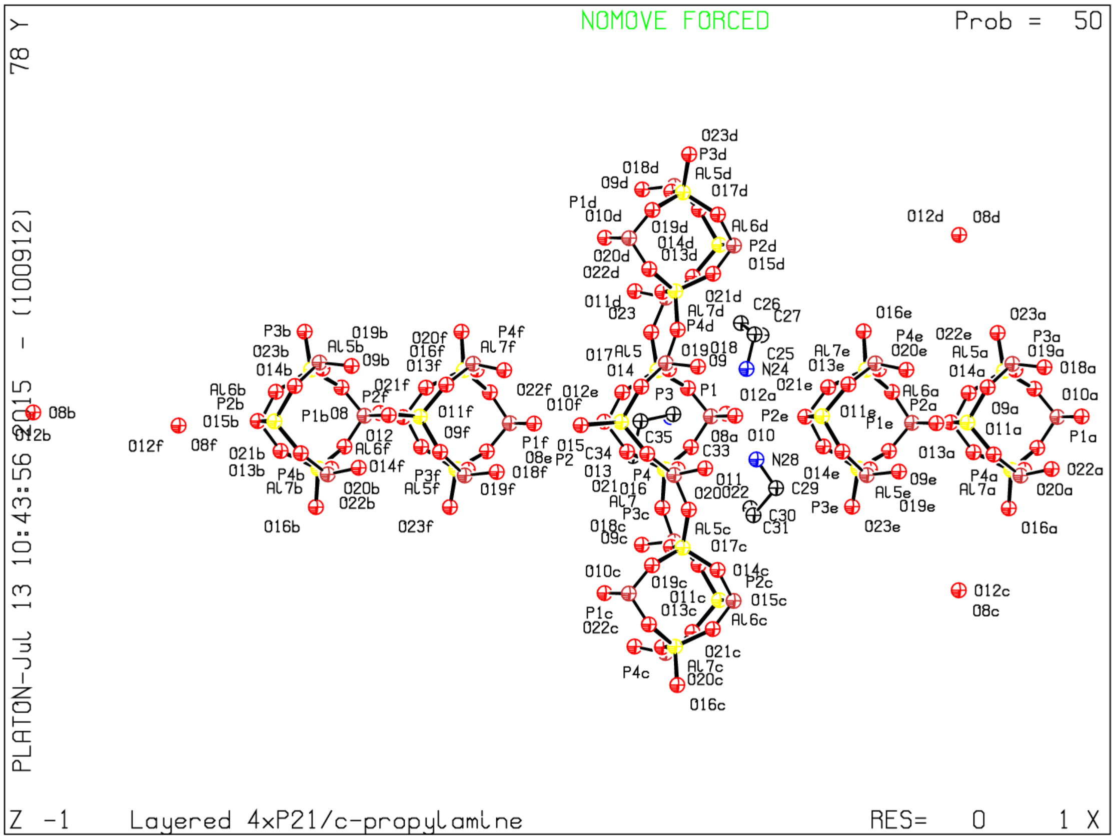

checkCIF/PLATON report

You have not supplied any structure factors. As a result the full set of tests cannot be run.

THIS REPORT IS FOR GUIDANCE ONLY. IF USED AS PART OF A REVIEW PROCEDURE FOR PUBLICATION, IT SHOULD NOT REPLACE THE EXPERTISE OF AN EXPERIENCED CRYSTALLOGRAPHIC REFEREE.

No syntax errors found. CIF dictionary Interpreting this report

Datablock: Layered_4x6x8_tetramethylethylenediamine

Bond precision: = 0.0000 A Wavelength=0.71073

Cell:	a=8.99070	b=9.83590	c=14.55660
	alpha=75.8720	beta=88.6160	gamma=63.4040
Temperature:	0 K		

	Calculated	Reported
Volume	1110.845	0
Space group	P -1	P-1
Hall group	-P 1	?
Moiety formula	Al3 O16 P4, 0.5(C8 N2), 0.5(C6 N2), O	?
Sum formula	C7 Al3 N2 O17 P4	?
Mr	588.91	0.00
Dx,g cm-3	1.761	0.000
Z	2	0
Mu (mm-1)	0.540	0.000
F000	582.0	0.0
F000’	583.54	
h,k,lmax		
Nref		
Tmin,Tmax		
Tmin’		

Correction method= Not given

Data completeness= Theta(max)=

R(reflections)= wR2(reflections)=

S = Npar=

The following ALERTS were generated. Each ALERT has the format
test-name_ALERT_alert-type_alert-level.
Click on the hyperlinks for more details of the test.

Alert level A

```
EXPT005_ALERT_1_A  _exptl_crystal_description is missing
            Crystal habit description.
            The following tests will not be performed.
            CRYSR_01
DIFF003_ALERT_1_A  _diffrn_measurement_device_type is missing
            Diffractometer make and type. Replaces _diffrn_measurement_type.
ATOM007_ALERT_1_A  _atom_site_aniso_label is missing
            Unique label identifying the atom site.
GEOM006_ALERT_1_A  _geom_angle_atom_site_label_2 is missing
            Label identifying the atom site 2.
GEOM007_ALERT_1_A  _geom_angle_atom_site_label_3 is missing
            Label identifying the atom site 3.
PLAT029_ALERT_3_A _diffrn_measured_fraction_theta_full Low .......      0.000 Note
PLAT043_ALERT_1_A Calculated and Reported Mol. Weight Differ by ..     588.91 Check
PLAT091_ALERT_1_A No Wavelength found in CIF - 0.71073 Ang Assumed     Please Check
PLAT197_ALERT_1_A Missing            _cell_measurement_temperature     Please Suppl
PLAT198_ALERT_1_A Missing              _diffrn_ambient_temperature     Please Suppl
```

Alert level B

```
PLAT306_ALERT_2_B Isolated Oxygen Atom (H-atoms Missing ?) .......        O1W Check
```

Alert level C

```
PLAT141_ALERT_4_C su on a - Axis Small or Missing ................    0.00000 Ang.
PLAT142_ALERT_4_C su on b - Axis Small or Missing ................    0.00000 Ang.
PLAT143_ALERT_4_C su on c - Axis Small or Missing ................    0.00000 Ang.
PLAT144_ALERT_4_C su on alpha    Small or Missing ................     0.0000 Degree
PLAT145_ALERT_4_C su on beta     Small or Missing ................     0.0000 Degree
PLAT146_ALERT_4_C su on gamma    Small or Missing ................     0.0000 Degree
PLAT161_ALERT_4_C Missing or Zero su (esd) on x-coordinate for ...        AL1
PLAT161_ALERT_4_C Missing or Zero su (esd) on x-coordinate for ...        AL2
PLAT161_ALERT_4_C Missing or Zero su (esd) on x-coordinate for ...        AL3
PLAT161_ALERT_4_C Missing or Zero su (esd) on x-coordinate for ...         P1
PLAT161_ALERT_4_C Missing or Zero su (esd) on x-coordinate for ...         P2
PLAT161_ALERT_4_C Missing or Zero su (esd) on x-coordinate for ...         P3
PLAT161_ALERT_4_C Missing or Zero su (esd) on x-coordinate for ...         P4
PLAT161_ALERT_4_C Missing or Zero su (esd) on x-coordinate for ...         O1
PLAT161_ALERT_4_C Missing or Zero su (esd) on x-coordinate for ...         O2
PLAT161_ALERT_4_C Missing or Zero su (esd) on x-coordinate for ...         O3
PLAT161_ALERT_4_C Missing or Zero su (esd) on x-coordinate for ...         O4
PLAT161_ALERT_4_C Missing or Zero su (esd) on x-coordinate for ...         O5
PLAT161_ALERT_4_C Missing or Zero su (esd) on x-coordinate for ...         O6
PLAT161_ALERT_4_C Missing or Zero su (esd) on x-coordinate for ...         O7
PLAT161_ALERT_4_C Missing or Zero su (esd) on x-coordinate for ...         O8
PLAT161_ALERT_4_C Missing or Zero su (esd) on x-coordinate for ...         O9
PLAT161_ALERT_4_C Missing or Zero su (esd) on x-coordinate for ...        O10
PLAT161_ALERT_4_C Missing or Zero su (esd) on x-coordinate for ...        O11
PLAT161_ALERT_4_C Missing or Zero su (esd) on x-coordinate for ...        O12
PLAT161_ALERT_4_C Missing or Zero su (esd) on x-coordinate for ...        O13
PLAT161_ALERT_4_C Missing or Zero su (esd) on x-coordinate for ...        O14
PLAT161_ALERT_4_C Missing or Zero su (esd) on x-coordinate for ...        O15
PLAT161_ALERT_4_C Missing or Zero su (esd) on x-coordinate for ...        O16
PLAT161_ALERT_4_C Missing or Zero su (esd) on x-coordinate for ...        O1W
PLAT161_ALERT_4_C Missing or Zero su (esd) on x-coordinate for ...         C1
PLAT161_ALERT_4_C Missing or Zero su (esd) on x-coordinate for ...        C10
PLAT161_ALERT_4_C Missing or Zero su (esd) on x-coordinate for ...         C2
PLAT161_ALERT_4_C Missing or Zero su (esd) on x-coordinate for ...         C3
PLAT161_ALERT_4_C Missing or Zero su (esd) on x-coordinate for ...         C4
PLAT161_ALERT_4_C Missing or Zero su (esd) on x-coordinate for ...         C5
```

```
PLAT161_ALERT_4_C Missing or Zero su (esd) on x-coordinate for ...         C6
PLAT161_ALERT_4_C Missing or Zero su (esd) on x-coordinate for ...         N1
PLAT161_ALERT_4_C Missing or Zero su (esd) on x-coordinate for ...         N2
PLAT162_ALERT_4_C Missing or Zero su (esd) on y-coordinate for ...        AL1
PLAT162_ALERT_4_C Missing or Zero su (esd) on y-coordinate for ...        AL2
PLAT162_ALERT_4_C Missing or Zero su (esd) on y-coordinate for ...        AL3
PLAT162_ALERT_4_C Missing or Zero su (esd) on y-coordinate for ...         P1
PLAT162_ALERT_4_C Missing or Zero su (esd) on y-coordinate for ...         P2
PLAT162_ALERT_4_C Missing or Zero su (esd) on y-coordinate for ...         P3
PLAT162_ALERT_4_C Missing or Zero su (esd) on y-coordinate for ...         P4
PLAT162_ALERT_4_C Missing or Zero su (esd) on y-coordinate for ...         O1
PLAT162_ALERT_4_C Missing or Zero su (esd) on y-coordinate for ...         O2
PLAT162_ALERT_4_C Missing or Zero su (esd) on y-coordinate for ...         O3
PLAT162_ALERT_4_C Missing or Zero su (esd) on y-coordinate for ...         O4
PLAT162_ALERT_4_C Missing or Zero su (esd) on y-coordinate for ...         O5
PLAT162_ALERT_4_C Missing or Zero su (esd) on y-coordinate for ...         O6
PLAT162_ALERT_4_C Missing or Zero su (esd) on y-coordinate for ...         O7
PLAT162_ALERT_4_C Missing or Zero su (esd) on y-coordinate for ...         O8
PLAT162_ALERT_4_C Missing or Zero su (esd) on y-coordinate for ...         O9
PLAT162_ALERT_4_C Missing or Zero su (esd) on y-coordinate for ...        O10
PLAT162_ALERT_4_C Missing or Zero su (esd) on y-coordinate for ...        O11
PLAT162_ALERT_4_C Missing or Zero su (esd) on y-coordinate for ...        O12
PLAT162_ALERT_4_C Missing or Zero su (esd) on y-coordinate for ...        O13
PLAT162_ALERT_4_C Missing or Zero su (esd) on y-coordinate for ...        O14
PLAT162_ALERT_4_C Missing or Zero su (esd) on y-coordinate for ...        O15
PLAT162_ALERT_4_C Missing or Zero su (esd) on y-coordinate for ...        O16
PLAT162_ALERT_4_C Missing or Zero su (esd) on y-coordinate for ...        O1W
PLAT162_ALERT_4_C Missing or Zero su (esd) on y-coordinate for ...         C1
PLAT162_ALERT_4_C Missing or Zero su (esd) on y-coordinate for ...        C10
PLAT162_ALERT_4_C Missing or Zero su (esd) on y-coordinate for ...         C2
PLAT162_ALERT_4_C Missing or Zero su (esd) on y-coordinate for ...         C3
PLAT162_ALERT_4_C Missing or Zero su (esd) on y-coordinate for ...         C4
PLAT162_ALERT_4_C Missing or Zero su (esd) on y-coordinate for ...         C5
PLAT162_ALERT_4_C Missing or Zero su (esd) on y-coordinate for ...         C6
PLAT162_ALERT_4_C Missing or Zero su (esd) on y-coordinate for ...         N1
PLAT162_ALERT_4_C Missing or Zero su (esd) on y-coordinate for ...         N2
PLAT163_ALERT_4_C Missing or Zero su (esd) on z-coordinate for ...        AL1
PLAT163_ALERT_4_C Missing or Zero su (esd) on z-coordinate for ...        AL2
PLAT163_ALERT_4_C Missing or Zero su (esd) on z-coordinate for ...        AL3
PLAT163_ALERT_4_C Missing or Zero su (esd) on z-coordinate for ...         P1
PLAT163_ALERT_4_C Missing or Zero su (esd) on z-coordinate for ...         P2
PLAT163_ALERT_4_C Missing or Zero su (esd) on z-coordinate for ...         P3
PLAT163_ALERT_4_C Missing or Zero su (esd) on z-coordinate for ...         P4
PLAT163_ALERT_4_C Missing or Zero su (esd) on z-coordinate for ...         O1
PLAT163_ALERT_4_C Missing or Zero su (esd) on z-coordinate for ...         O2
PLAT163_ALERT_4_C Missing or Zero su (esd) on z-coordinate for ...         O3
PLAT163_ALERT_4_C Missing or Zero su (esd) on z-coordinate for ...         O4
PLAT163_ALERT_4_C Missing or Zero su (esd) on z-coordinate for ...         O5
PLAT163_ALERT_4_C Missing or Zero su (esd) on z-coordinate for ...         O6
PLAT163_ALERT_4_C Missing or Zero su (esd) on z-coordinate for ...         O7
PLAT163_ALERT_4_C Missing or Zero su (esd) on z-coordinate for ...         O8
PLAT163_ALERT_4_C Missing or Zero su (esd) on z-coordinate for ...         O9
PLAT163_ALERT_4_C Missing or Zero su (esd) on z-coordinate for ...        O10
PLAT163_ALERT_4_C Missing or Zero su (esd) on z-coordinate for ...        O11
PLAT163_ALERT_4_C Missing or Zero su (esd) on z-coordinate for ...        O12
PLAT163_ALERT_4_C Missing or Zero su (esd) on z-coordinate for ...        O13
PLAT163_ALERT_4_C Missing or Zero su (esd) on z-coordinate for ...        O14
PLAT163_ALERT_4_C Missing or Zero su (esd) on z-coordinate for ...        O15
PLAT163_ALERT_4_C Missing or Zero su (esd) on z-coordinate for ...        O16
PLAT163_ALERT_4_C Missing or Zero su (esd) on z-coordinate for ...        O1W
PLAT163_ALERT_4_C Missing or Zero su (esd) on z-coordinate for ...         C1
PLAT163_ALERT_4_C Missing or Zero su (esd) on z-coordinate for ...        C10
```

```
PLAT163_ALERT_4_C Missing or Zero su (esd) on z-coordinate for ...         C2
PLAT163_ALERT_4_C Missing or Zero su (esd) on z-coordinate for ...         C3
PLAT163_ALERT_4_C Missing or Zero su (esd) on z-coordinate for ...         C4
PLAT163_ALERT_4_C Missing or Zero su (esd) on z-coordinate for ...         C5
PLAT163_ALERT_4_C Missing or Zero su (esd) on z-coordinate for ...         C6
PLAT163_ALERT_4_C Missing or Zero su (esd) on z-coordinate for ...         N1
PLAT163_ALERT_4_C Missing or Zero su (esd) on z-coordinate for ...         N2
PLAT202_ALERT_3_C Isotropic non-H Atoms in Anion/Solvent .........          5
PLAT241_ALERT_2_C High      Ueq as Compared to Neighbors for .....         O1 Check
PLAT242_ALERT_2_C Low       Ueq as Compared to Neighbors for .....         P2 Check
PLAT242_ALERT_2_C Low       Ueq as Compared to Neighbors for .....         P3 Check
PLAT242_ALERT_2_C Low       Ueq as Compared to Neighbors for .....         N1 Check
PLAT244_ALERT_4_C Low   'Solvent' Ueq as Compared to Neighbors of          N2 Check
```

Alert level G

```
PLAT004_ALERT_5_G Polymeric Structure Found with Maximum Dimension          2 Info
PLAT005_ALERT_5_G No _iucr_refine_instructions_details  in the CIF     Please Do !
PLAT040_ALERT_1_G No H-atoms in this Carbon Containing Compound ..     Please Check
PLAT045_ALERT_1_G Calculated and Reported Z Differ by ............       0.00 Ratio
PLAT194_ALERT_1_G Missing _cell_measurement_reflns_used datum ....     Please Do !
PLAT195_ALERT_1_G Missing _cell_measurement_theta_max   datum ....     Please Do !
PLAT196_ALERT_1_G Missing _cell_measurement_theta_min   datum ....     Please Do !
PLAT199_ALERT_1_G Reported _cell_measurement_temperature ..... (K)    -999999 Check
PLAT793_ALERT_4_G The Model has Chirality at P1      (Centro SPGR)          S Verify
PLAT793_ALERT_4_G The Model has Chirality at P2      (Centro SPGR)          S Verify
PLAT793_ALERT_4_G The Model has Chirality at P3      (Centro SPGR)          S Verify
PLAT793_ALERT_4_G The Model has Chirality at P4      (Centro SPGR)          R Verify
PLAT794_ALERT_5_G Tentative Bond Valency for Al1     (III)   .....       2.97 Note
PLAT794_ALERT_5_G Tentative Bond Valency for Al2     (III)   .....       3.07 Note
PLAT794_ALERT_5_G Tentative Bond Valency for Al3     (III)   .....       3.00 Note
PLAT808_ALERT_5_G No Parseable SHELXL Style Weighting Scheme Found     Please Check
PLAT980_ALERT_1_G No Anomalous Scattering Factors Found in CIF ...     Please Check
```

---

10 **ALERT level A** = Most likely a serious problem - resolve or explain
1 **ALERT level B** = A potentially serious problem, consider carefully
111 **ALERT level C** = Check. Ensure it is not caused by an omission or oversight
17 **ALERT level G** = General information/check it is not something unexpected

16 ALERT type 1 CIF construction/syntax error, inconsistent or missing data
5 ALERT type 2 Indicator that the structure model may be wrong or deficient
2 ALERT type 3 Indicator that the structure quality may be low
110 ALERT type 4 Improvement, methodology, query or suggestion
6 ALERT type 5 Informative message, check

It is advisable to attempt to resolve as many as possible of the alerts in all categories. Often the minor alerts point to easily fixed oversights, errors and omissions in your CIF or refinement strategy, so attention to these fine details can be worthwhile. In order to resolve some of the more serious problems it may be necessary to carry out additional measurements or structure refinements. However, the purpose of your study may justify the reported deviations and the more serious of these should normally be commented upon in the discussion or experimental section of a paper or in the "special_details" fields of the CIF. checkCIF was carefully designed to identify outliers and unusual parameters, but every test has its limitations and alerts that are not important in a particular case may appear. Conversely, the absence of alerts does not guarantee there are no aspects of the results needing attention. It is up to the individual to critically assess their own results and, if necessary, seek expert advice.

Publication of your CIF in IUCr journals

A basic structural check has been run on your CIF. These basic checks will be run on all CIFs submitted for publication in IUCr journals (*Acta Crystallographica*, *Journal of Applied Crystallography*, *Journal of Synchrotron Radiation*); however, if you intend to submit to *Acta Crystallographica Section C* or *E*, you should make sure that full publication checks are run on the final version of your CIF prior to submission.

Publication of your CIF in other journals

Please refer to the *Notes for Authors* of the relevant journal for any special instructions relating to CIF submission.

PLATON version of 21/06/2015; check.def file version of 21/06/2015

Datablock Layered_4x6x8_tetramethylethylenediamine - ellipsoid plot

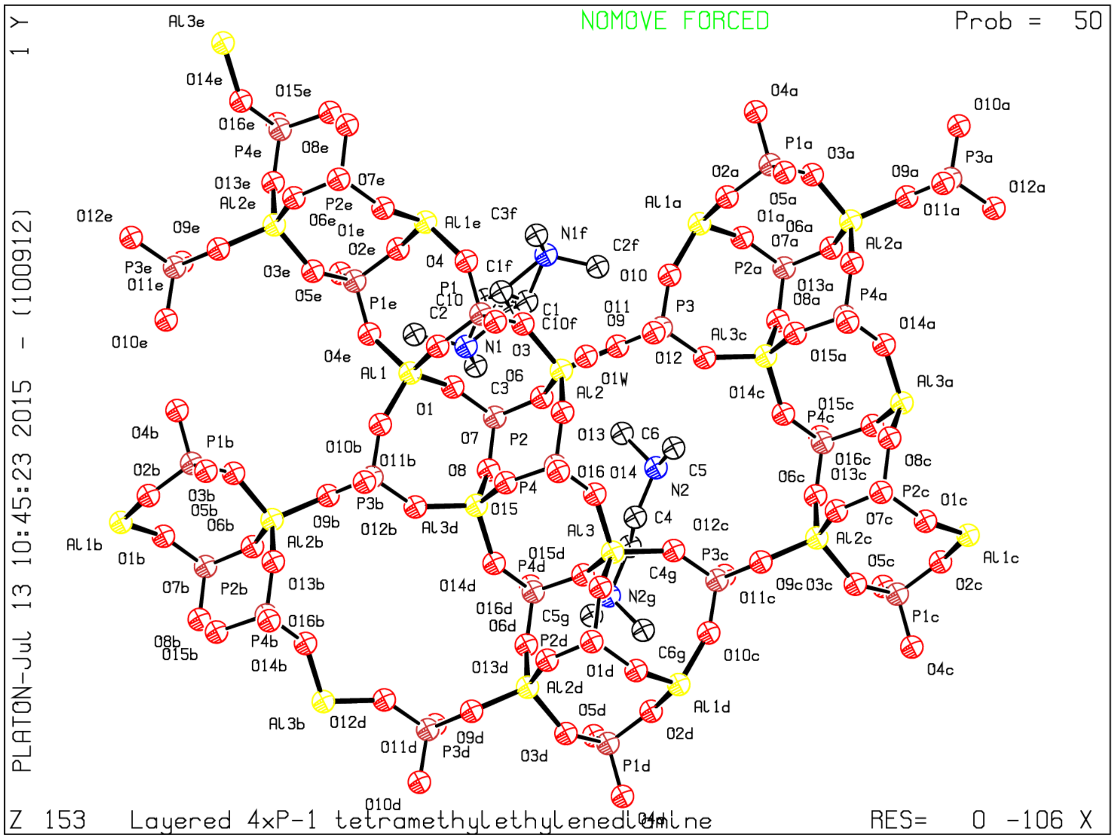

checkCIF/PLATON report

You have not supplied any structure factors. As a result the full set of tests cannot be run.

THIS REPORT IS FOR GUIDANCE ONLY. IF USED AS PART OF A REVIEW PROCEDURE FOR PUBLICATION, IT SHOULD NOT REPLACE THE EXPERTISE OF AN EXPERIENCED CRYSTALLOGRAPHIC REFEREE.

No syntax errors found. CIF dictionary Interpreting this report

Datablock: AUI

```
Bond precision:  C-C = 0.0035 A          Wavelength=0.71073

Cell:           a=8.215(2)          b=8.810(3)        c=8.861(3)
                alpha=88.001(4)     beta=89.818(5)    gamma=89.773(5)
Temperature:    295 K
```

	Calculated	Reported
Volume	640.9(3)	640.9(3)
Space group	P -1	P -1
Hall group	-P 1	-P 1
Moiety formula	H2 O8 P2 Zn, C4 H14 N2, H2 O	H2 O8 P2 Zn, (C4 H14 N2), H2 O
Sum formula	C4 H18 N2 O9 P2 Zn	C4 H18 N2 O9 P2 Zn
Mr	365.53	365.53
Dx,g cm-3	1.894	1.894
Z	2	2
Mu (mm-1)	2.206	2.206
F000	376.0	376.0
F000’	377.16	
h,k,lmax	11,12,12	11,12,12
Nref	3677	3599
Tmin,Tmax	0.627,0.785	0.531,0.791
Tmin’	0.410	

```
Correction method= # Reported T Limits: Tmin=0.531 Tmax=0.791
AbsCorr = INTEGRATION

Data completeness= 0.979          Theta(max)= 29.800

R(reflections)= 0.0326( 3302)     wR2(reflections)= 0.0900( 3597)

S = 1.027                   Npar= 167
```

The following ALERTS were generated. Each ALERT has the format
test-name_ALERT_alert-type_alert-level.
Click on the hyperlinks for more details of the test.

Alert level C

```
RINTA01_ALERT_3_C  The value of Rint is greater than 0.12
            Rint given   0.127
```

Alert level G

```
PLAT004_ALERT_5_G Polymeric Structure Found with Maximum Dimension          1 Info
PLAT005_ALERT_5_G No _iucr_refine_instructions_details  in the CIF     Please Do !
PLAT007_ALERT_5_G Number of Unrefined Donor-H Atoms ..............          8 Report
PLAT042_ALERT_1_G Calc. and Reported MoietyFormula Strings  Differ     Please Check
PLAT093_ALERT_1_G No su's on H-positions, refinement reported as .      mixed Check
PLAT720_ALERT_4_G Number of Unusual/Non-Standard Labels ..........          2 Note
PLAT793_ALERT_4_G The Model has Chirality at P1      (Centro SPGR)          R Verify
PLAT793_ALERT_4_G The Model has Chirality at P2      (Centro SPGR)          S Verify
PLAT794_ALERT_5_G Tentative Bond Valency for Zn1     (II)    .....       2.13 Note
PLAT980_ALERT_1_G No Anomalous Scattering Factors Found in CIF ...     Please Check
```

0 **ALERT level A** = Most likely a serious problem - resolve or explain
0 **ALERT level B** = A potentially serious problem, consider carefully
1 **ALERT level C** = Check. Ensure it is not caused by an omission or oversight
10 **ALERT level G** = General information/check it is not something unexpected

3 ALERT type 1 CIF construction/syntax error, inconsistent or missing data
0 ALERT type 2 Indicator that the structure model may be wrong or deficient
1 ALERT type 3 Indicator that the structure quality may be low
3 ALERT type 4 Improvement, methodology, query or suggestion
4 ALERT type 5 Informative message, check

Datablock: AUII

Bond precision: C-C = 0.0030 A   Wavelength=0.71073

Cell:	a=11.7877(4)	b=5.2093(2)	c=12.2031(4)
	alpha=90	beta=98.198(1)	gamma=90
Temperature:	295 K		

	Calculated	Reported
Volume	741.68(5)	741.68(5)
Space group	P 2/n	P 2/n
Hall group	-P 2yac	-P 2yac
Moiety formula	H3 O12 P3 Zn2, C4 H14 N2	H3 O12 P3 Zn2, 2(C2 H7 N)
Sum formula	C4 H17 N2 O12 P3 Zn2	C4 H17 N2 O12 P3 Zn2
Mr	508.89	508.89
Dx,g cm-3	2.279	2.278
Z	2	2
Mu (mm-1)	3.623	3.623
F000	512.0	512.0
F000’	513.99	
h,k,lmax	16,7,17	16,7,17
Nref	2275	2174
Tmin,Tmax	0.400,0.647	0.359,0.667
Tmin’	0.210	

Correction method= # Reported T Limits: Tmin=0.359 Tmax=0.667
AbsCorr = INTEGRATION

Data completeness= 0.956 Theta(max)= 30.520

R(reflections)= 0.0267(2032) wR2(reflections)= 0.0745(2174)

S = 1.098 Npar= 108

The following ALERTS were generated. Each ALERT has the format
test-name_ALERT_alert-type_alert-level.
Click on the hyperlinks for more details of the test.

Alert level B

PLAT029_ALERT_3_B _diffrn_measured_fraction_theta_full Low .... 0.955 Note

---

Alert level G

PLAT004_ALERT_5_G Polymeric Structure Found with Maximum Dimension 2 Info
PLAT005_ALERT_5_G No _iucr_refine_instructions_details in the CIF Please Do !
PLAT007_ALERT_5_G Number of Unrefined Donor-H Atoms .... 4 Report
PLAT042_ALERT_1_G Calc. and Reported MoietyFormula Strings Differ Please Check
PLAT093_ALERT_1_G No su’s on H-positions, refinement reported as . mixed Check
PLAT300_ALERT_4_G Atom Site Occupancy of *H1O is Constrained at 0.500 Check
PLAT720_ALERT_4_G Number of Unusual/Non-Standard Labels .... 2 Note
PLAT794_ALERT_5_G Tentative Bond Valency for Zn1 (II) ..... 2.21 Note
PLAT899_ALERT_4_G SHELXL97 is Deprecated and Succeeded by SHELXL 2014 Note

---

0 **ALERT level A** = Most likely a serious problem - resolve or explain
1 **ALERT level B** = A potentially serious problem, consider carefully
0 **ALERT level C** = Check. Ensure it is not caused by an omission or oversight
9 **ALERT level G** = General information/check it is not something unexpected

2 ALERT type 1 CIF construction/syntax error, inconsistent or missing data

```
 0 ALERT type 2 Indicator that the structure model may be wrong or deficient
 1 ALERT type 3 Indicator that the structure quality may be low
 3 ALERT type 4 Improvement, methodology, query or suggestion
 4 ALERT type 5 Informative message, check
```

Datablock: AUIII

```
Bond precision:  C-C = 0.0080 A          Wavelength=0.71073

Cell:             a=20.723(2)     b=5.2095(6)     c=17.8741(19)
                  alpha=90        beta=90         gamma=90
Temperature:      295 K

                Calculated                  Reported
Volume          1929.6(4)                   1929.6(4)
Space group     P n a 21                    P n a 21
Hall group      P 2c -2n                    P 2c -2n
Moiety formula  H2 O17 P4 Zn5, C4 H14 N2    H2 O17 P4 Zn5, C4 H14 N2
Sum formula     C4 H16 N2 O17 P4 Zn5        C4 H16 N2 O17 P4 Zn5
Mr              815.02                      814.91
Dx,g cm-3       2.806                       2.805
Z               4                           4
Mu (mm-1)       6.548                       6.548
F000            1600.0                      1600.0
F000'           1608.03
h,k,lmax        29,7,25                     29,7,25
Nref            5957[ 3066]                 5712
Tmin,Tmax       0.320,0.770                 0.209,0.770
Tmin'           0.036

Correction method= # Reported T Limits: Tmin=0.209 Tmax=0.770
AbsCorr = MULTI-SCAN

Data completeness= 1.86/0.96      Theta(max)= 30.600

R(reflections)= 0.0341( 4887)     wR2(reflections)= 0.0709( 5712)

S = 1.030                   Npar= 310
```

The following ALERTS were generated. Each ALERT has the format
test-name_ALERT_alert-type_alert-level.
Click on the hyperlinks for more details of the test.

Alert level C

ABSTY02_ALERT_1_C An _exptl_absorpt_correction_type has been given without
a literature citation. This should be contained in the
_exptl_absorpt_process_details field.
Absorption correction given as multi-scan

```
RINTA01_ALERT_3_C  The value of Rint is greater than 0.12
            Rint given   0.136
PLAT029_ALERT_3_C _diffrn_measured_fraction_theta_full Low .......      0.979 Note
PLAT090_ALERT_3_C Poor Data / Parameter Ratio (Zmax > 18) ........       9.89 Note
PLAT341_ALERT_3_C Low Bond Precision on  C-C Bonds ..............     0.0080 Ang.
```

Alert level G

```
PLAT004_ALERT_5_G Polymeric Structure Found with Maximum Dimension          3 Info
PLAT005_ALERT_5_G No _iucr_refine_instructions_details  in the CIF     Please Do !
PLAT007_ALERT_5_G Number of Unrefined Donor-H Atoms .............           1 Report
PLAT720_ALERT_4_G Number of Unusual/Non-Standard Labels ..........          6 Note
PLAT792_ALERT_1_G The Model has Chirality at P2      (Polar  SPGR)          S Verify
PLAT792_ALERT_1_G The Model has Chirality at P3      (Polar  SPGR)          R Verify
PLAT792_ALERT_1_G The Model has Chirality at P4      (Polar  SPGR)          S Verify
PLAT794_ALERT_5_G Tentative Bond Valency for Zn5     (II)    .....       2.10 Note
```

---

0 **ALERT level A** = Most likely a serious problem - resolve or explain
0 **ALERT level B** = A potentially serious problem, consider carefully
5 **ALERT level C** = Check. Ensure it is not caused by an omission or oversight
8 **ALERT level G** = General information/check it is not something unexpected

4 ALERT type 1 CIF construction/syntax error, inconsistent or missing data
0 ALERT type 2 Indicator that the structure model may be wrong or deficient
4 ALERT type 3 Indicator that the structure quality may be low
1 ALERT type 4 Improvement, methodology, query or suggestion
4 ALERT type 5 Informative message, check

It is advisable to attempt to resolve as many as possible of the alerts in all categories. Often the minor alerts point to easily fixed oversights, errors and omissions in your CIF or refinement strategy, so attention to these fine details can be worthwhile. In order to resolve some of the more serious problems it may be necessary to carry out additional measurements or structure refinements. However, the purpose of your study may justify the reported deviations and the more serious of these should normally be commented upon in the discussion or experimental section of a paper or in the "special_details" fields of the CIF. checkCIF was carefully designed to identify outliers and unusual parameters, but every test has its limitations and alerts that are not important in a particular case may appear. Conversely, the absence of alerts does not guarantee there are no aspects of the results needing attention. It is up to the individual to critically assess their own results and, if necessary, seek expert advice.

Publication of your CIF in IUCr journals

A basic structural check has been run on your CIF. These basic checks will be run on all CIFs submitted for publication in IUCr journals (*Acta Crystallographica*, *Journal of Applied Crystallography*, *Journal of Synchrotron Radiation*); however, if you intend to submit to *Acta Crystallographica Section C* or *E*, you should make sure that full publication checks are run on the final version of your CIF prior to submission.

Publication of your CIF in other journals

Please refer to the *Notes for Authors* of the relevant journal for any special instructions relating to CIF submission.

PLATON version of 21/06/2015; check.def file version of 21/06/2015

Datablock AUI - ellipsoid plot

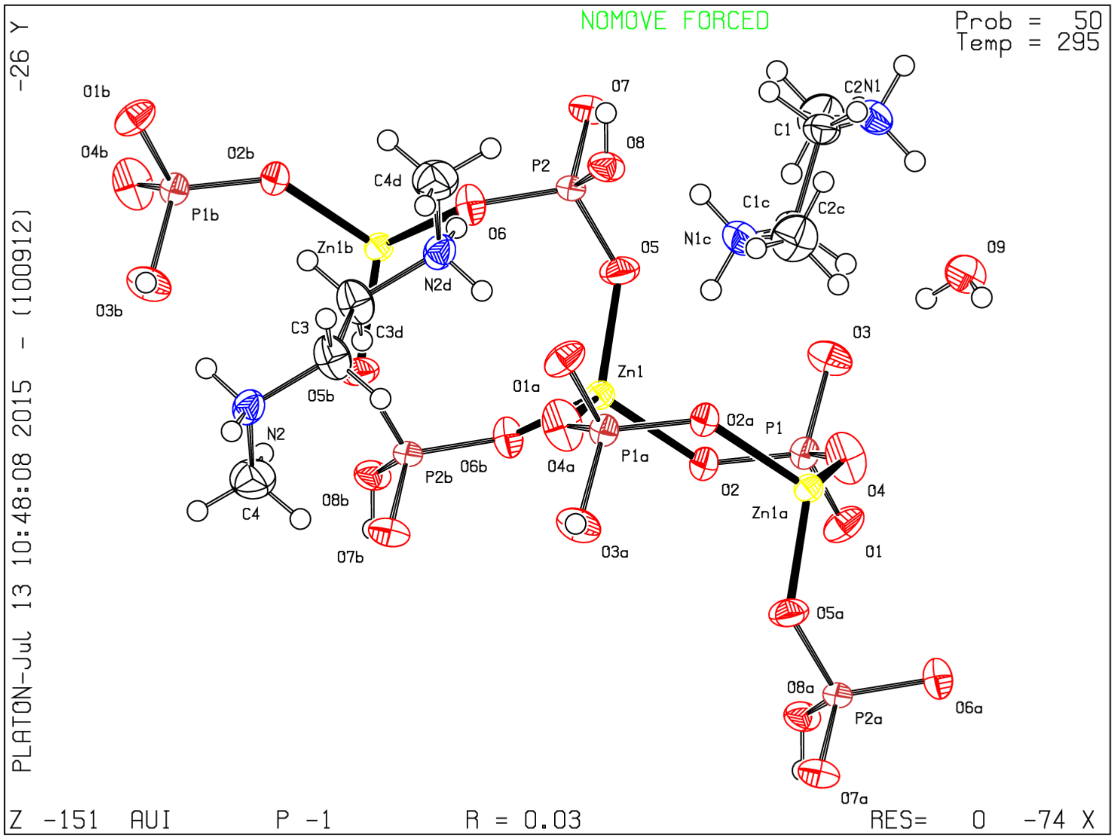

Datablock AUII - ellipsoid plot

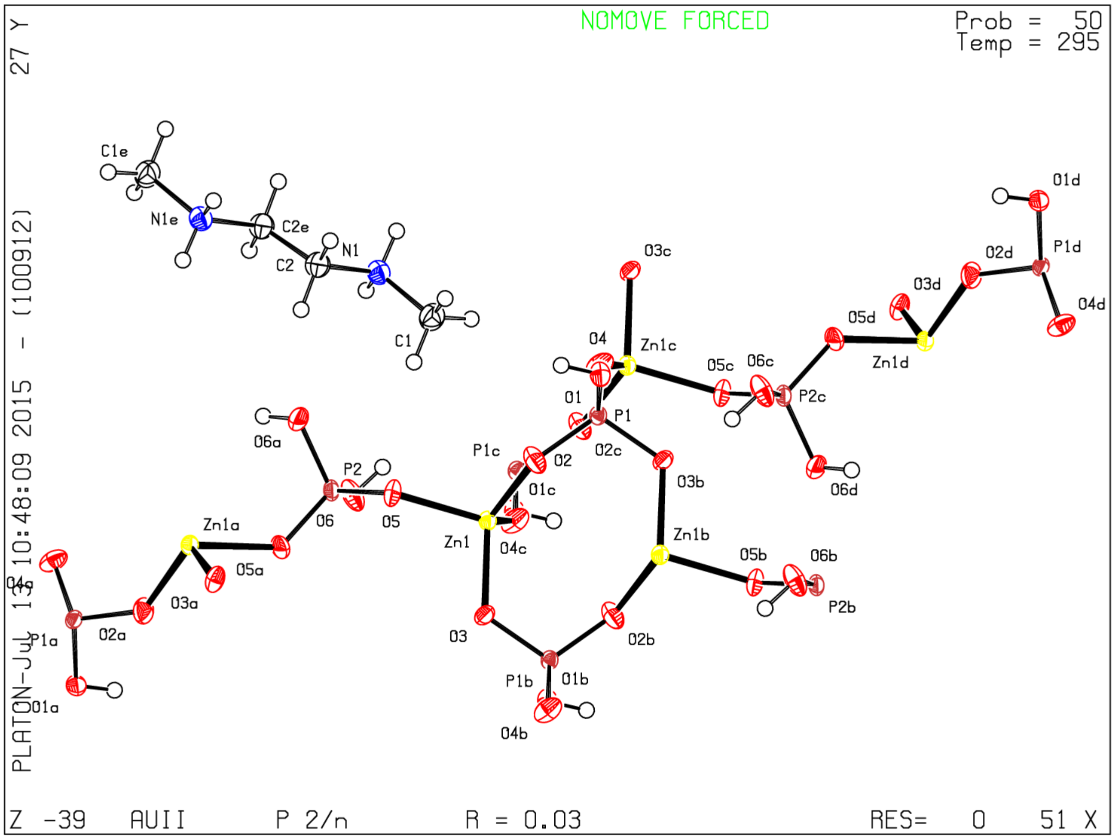

Datablock AUIII - ellipsoid plot

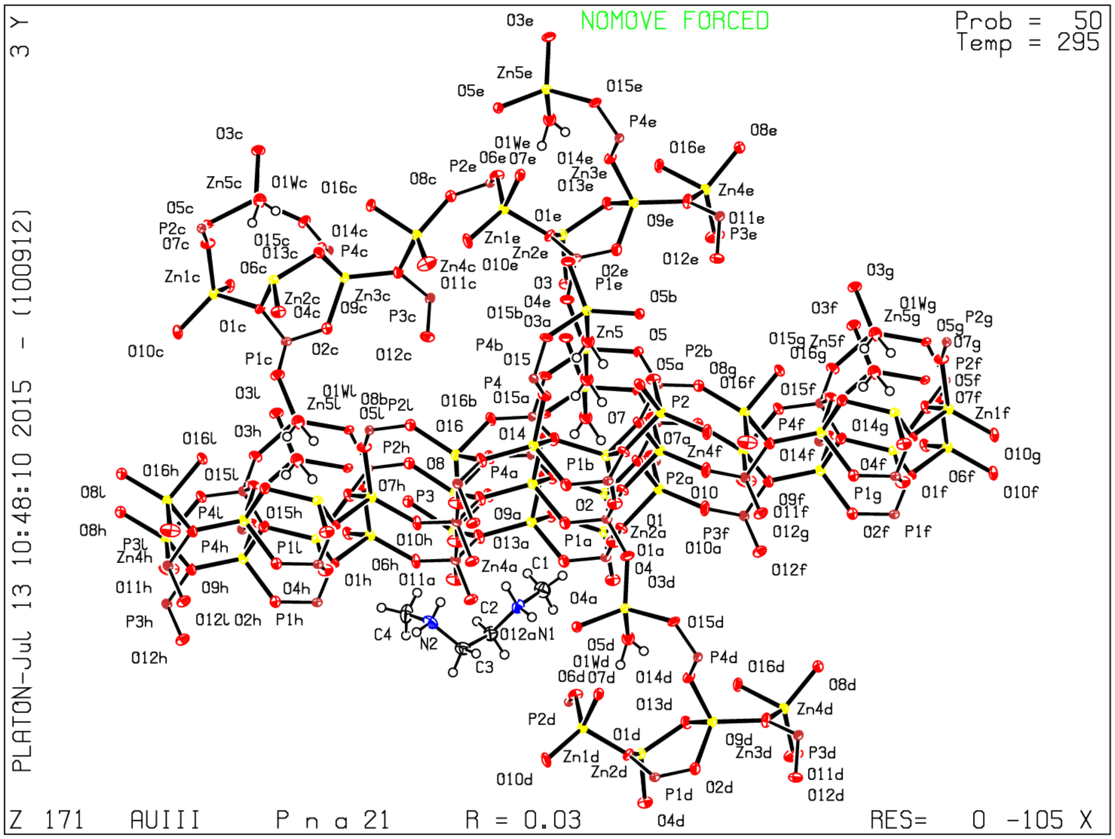

checkCIF/PLATON report

You have not supplied any structure factors. As a result the full set of tests cannot be run.

THIS REPORT IS FOR GUIDANCE ONLY. IF USED AS PART OF A REVIEW PROCEDURE FOR PUBLICATION, IT SHOULD NOT REPLACE THE EXPERTISE OF AN EXPERIENCED CRYSTALLOGRAPHIC REFEREE.

No syntax errors found. CIF dictionary Interpreting this report

Datablock: I

```
Bond precision:  C-C = 0.0095 A          Wavelength=0.71073

Cell:             a=11.8920(2)     b=5.1318(1)       c=12.3063(2)
                  alpha=90         beta=98.125(1)    gamma=90
Temperature:      293 K
```

	Calculated	Reported
Volume	743.48(2)	743.48(2)
Space group	P n	P n
Hall group	P -2yac	P -2yac
Moiety formula	H3 O12 P3 Zn2, C4 H14 N2	C4 H14 N2 2+, H3 O12 P3 Zn2 2-
Sum formula	C4 H17 N2 O12 P3 Zn2	C4 H17 N2 O12 P3 Zn2
Mr	508.89	508.85
Dx,g cm-3	2.273	2.273
Z	2	2
Mu (mm-1)	3.614	3.614
F000	512.0	512.0
F000’	513.99	
h,k,lmax	15,6,15	15,6,15
Nref	3412[1709]	3108
Tmin,Tmax	0.490,0.835	0.490,0.835
Tmin’	0.481	

```
Correction method= # Reported T Limits: Tmin=0.490 Tmax=0.835
AbsCorr = MULTI-SCAN

Data completeness= 1.82/0.91      Theta(max)= 27.480

R(reflections)= 0.0281( 3029)     wR2(reflections)= 0.0865( 3108)

S = 1.187                  Npar= 214
```

The following ALERTS were generated. Each ALERT has the format
test-name_ALERT_alert-type_alert-level.
Click on the hyperlinks for more details of the test.

Alert level C

```
STRVA01_ALERT_4_C           Flack test results are ambiguous.
          From the CIF: _refine_ls_abs_structure_Flack    0.440
          From the CIF: _refine_ls_abs_structure_Flack_su    0.010
PLAT090_ALERT_3_C Poor Data / Parameter Ratio (Zmax > 18) ........       7.99 Note
PLAT193_ALERT_1_C Cell and Diffraction Temperatures differ by ....          2 Degree
PLAT250_ALERT_2_C Large U3/U1 Ratio for Average U(i,j) Tensor ....        2.3 Note
PLAT341_ALERT_3_C Low Bond Precision on  C-C Bonds ...............     0.0095 Ang.
```

Alert level G

```
PLAT004_ALERT_5_G Polymeric Structure Found with Maximum Dimension          2 Info
PLAT005_ALERT_5_G No _iucr_refine_instructions_details  in the CIF     Please Do !
PLAT007_ALERT_5_G Number of Unrefined Donor-H Atoms ..............          8 Report
PLAT042_ALERT_1_G Calc. and Reported MoietyFormula Strings  Differ     Please Check
PLAT066_ALERT_1_G Predicted and Reported Tmin&Tmax Range Identical          ? Check
PLAT111_ALERT_2_G ADDSYM Detects (Pseudo) Centre of Symmetry .....         86 %Fit
PLAT113_ALERT_2_G ADDSYM Suggests Possible Pseudo/New Space group.       P2/n Check
PLAT200_ALERT_1_G Reported   _diffrn_ambient_temperature ..... (K)        293 Check
PLAT792_ALERT_1_G The Model has Chirality at P2      (Polar  SPGR)          S Verify
PLAT792_ALERT_1_G The Model has Chirality at P3      (Polar  SPGR)          R Verify
PLAT794_ALERT_5_G Tentative Bond Valency for Zn1     (II)    .....       2.14 Note
PLAT794_ALERT_5_G Tentative Bond Valency for Zn2     (II)    .....       2.17 Note
```

---

0 **ALERT level A** = Most likely a serious problem - resolve or explain
0 **ALERT level B** = A potentially serious problem, consider carefully
5 **ALERT level C** = Check. Ensure it is not caused by an omission or oversight
12 **ALERT level G** = General information/check it is not something unexpected

6 ALERT type 1 CIF construction/syntax error, inconsistent or missing data
3 ALERT type 2 Indicator that the structure model may be wrong or deficient
2 ALERT type 3 Indicator that the structure quality may be low
1 ALERT type 4 Improvement, methodology, query or suggestion
5 ALERT type 5 Informative message, check

It is advisable to attempt to resolve as many as possible of the alerts in all categories. Often the minor alerts point to easily fixed oversights, errors and omissions in your CIF or refinement strategy, so attention to these fine details can be worthwhile. In order to resolve some of the more serious problems it may be necessary to carry out additional measurements or structure refinements. However, the purpose of your study may justify the reported deviations and the more serious of these should normally be commented upon in the discussion or experimental section of a paper or in the "special_details" fields of the CIF. checkCIF was carefully designed to identify outliers and unusual parameters, but every test has its limitations and alerts that are not important in a particular case may appear. Conversely, the absence of alerts does not guarantee there are no aspects of the results needing attention. It is up to the individual to critically assess their own results and, if necessary, seek expert advice.

Publication of your CIF in IUCr journals

A basic structural check has been run on your CIF. These basic checks will be run on all CIFs submitted for publication in IUCr journals (*Acta Crystallographica*, *Journal of Applied Crystallography*, *Journal of Synchrotron Radiation*); however, if you intend to submit to *Acta Crystallographica Section C* or *E*, you should make sure that full publication checks are run on the final version of your CIF prior to submission.

Publication of your CIF in other journals

Please refer to the *Notes for Authors* of the relevant journal for any special instructions relating to CIF submission.

PLATON version of 21/06/2015; check.def file version of 21/06/2015

Datablock I - ellipsoid plot

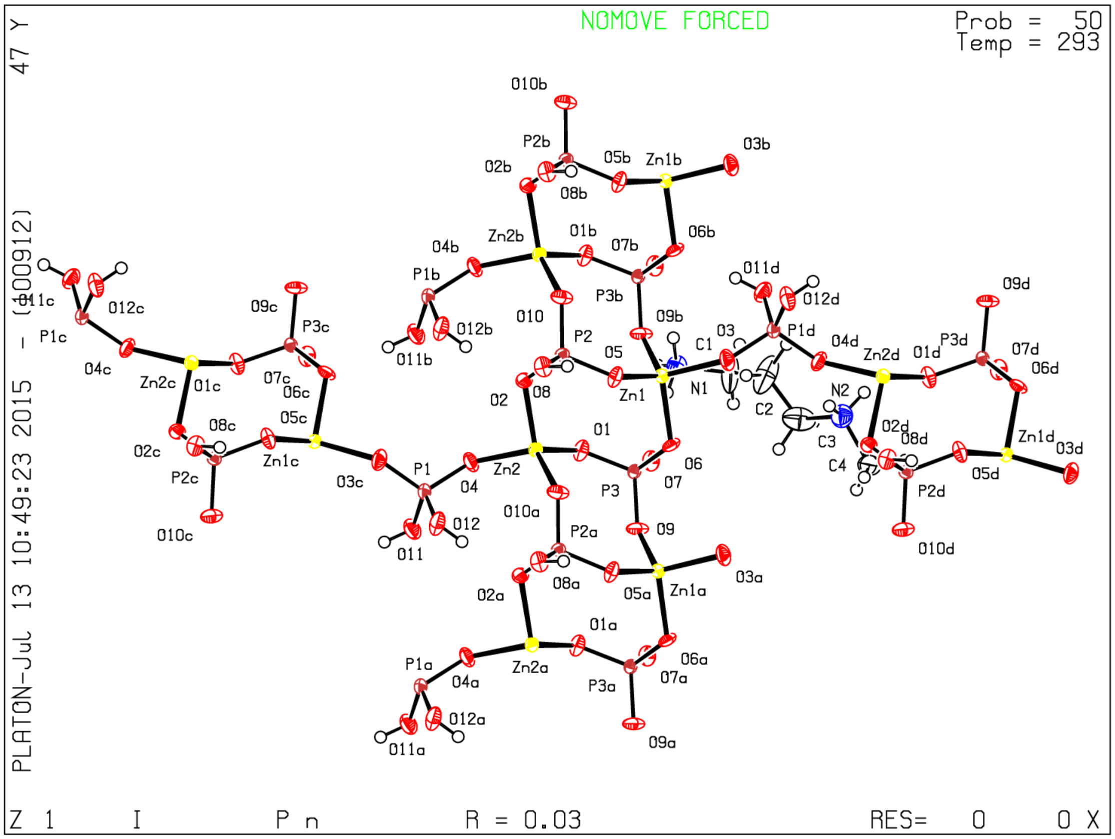

Supplement: Supplementary Material [file srep14940-s2.pdf]
